# Supplementary material for: Enzymatic carbon–fluorine bond cleavage by human gut microbes
Source: Proc Natl Acad Sci U S A. 2025 Jun 13;122(24):e2504122122. doi: 10.1073/pnas.2504122122 (PMC12184663; doi:10.1073/pnas.2504122122)
Supplement: Supplementary file 1 — Appendix 01 (PDF) [file pnas.2504122122.sapp.pdf]

Supplementary Information

Enzymatic carbon-fluorine bond cleavage by human gut microbes

Silke I. Probst[1], Florian D. Felder[1], Victoria Poltorak[1,2], Ritesh Mewalal[3], Ian K. Blaby[3], Serina L. Robinson[1*]

[1]Department of Environmental Microbiology, Swiss Federal Institute of Aquatic Science and Technology (Eawag), Dübendorf, Switzerland; [2]Department of Biology, Institute of Microbiology, Eidgenössische Technische Hochschule (ETH) Zürich, Zürich, Switzerland; [3]U.S. Department of Energy Joint Genome Institute, Lawrence Berkeley National Laboratory, Berkeley, CA, USA

*corresponding author: serina.robinson@eawag.ch

Supplementary Figures

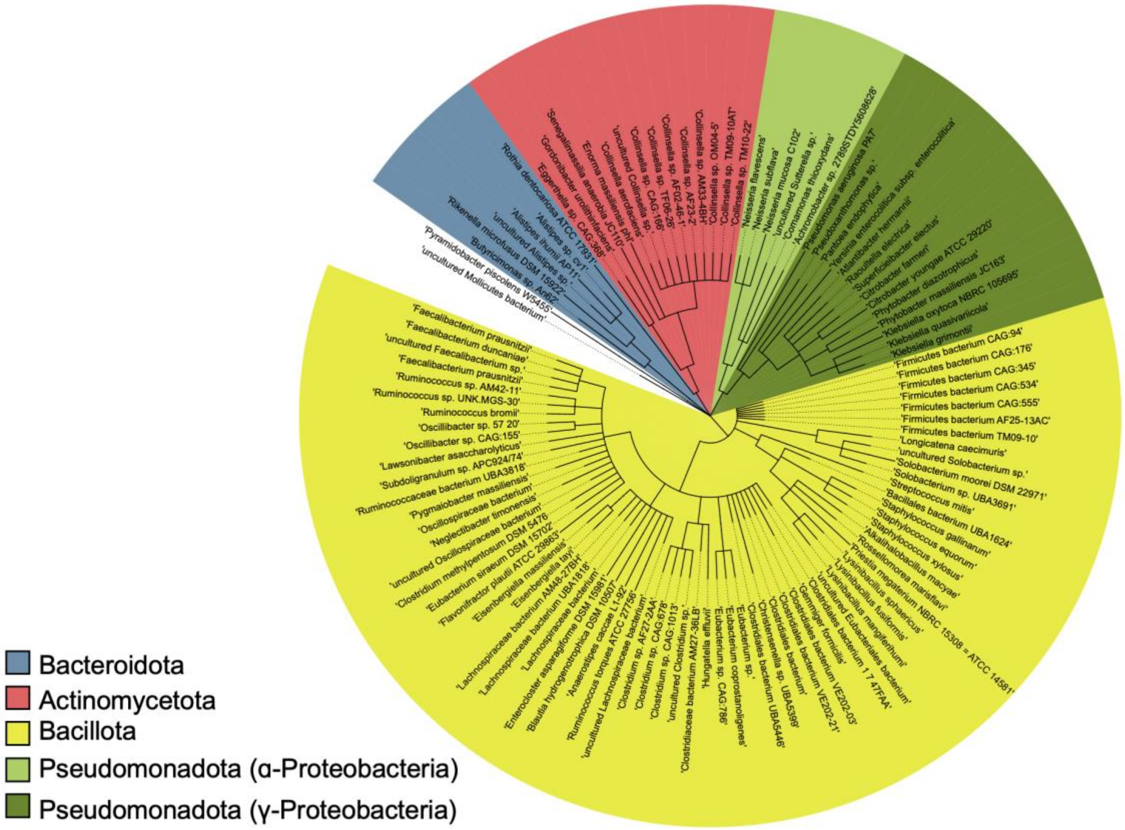

Figure S1: Phylogenetic tree of human gut microbial strains encoding homologs with a bitscore > 270 to the query haloacid dehalogenase from *Rhodococcus jostii* RHA1 (PDB ID: 3UMG).

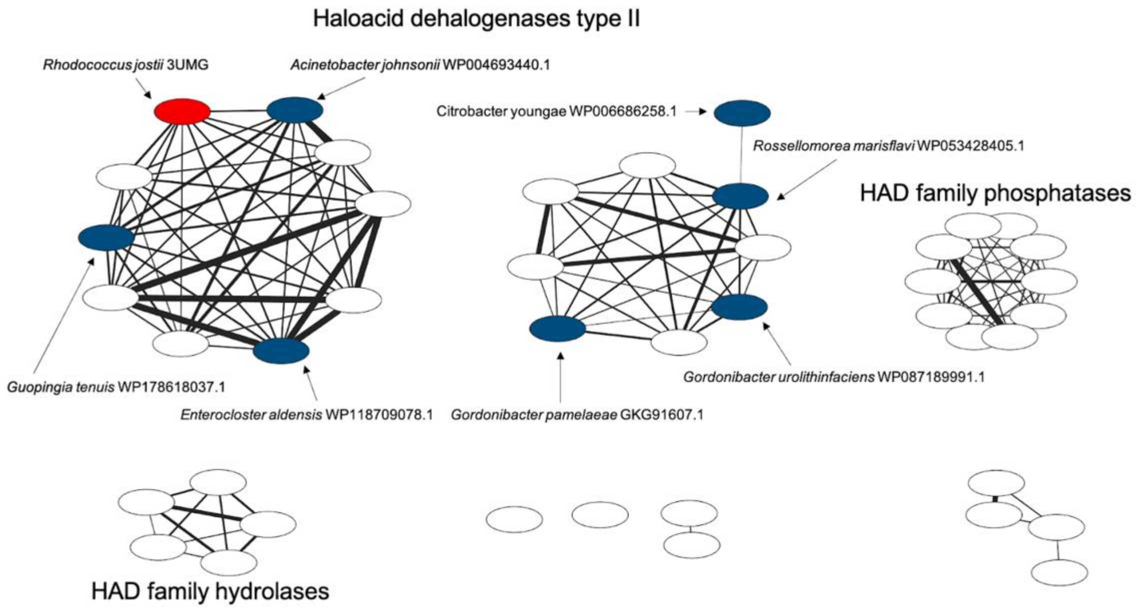

Figure S2: Protein sequence similarity network constructed from 60 protein sequences sharing homology to the 2-haloacid dehalogenases identified in the human gut microbiome. EFI-EST annotations of protein families are used. The width of interconnecting edges indicate the degree of relatedness between two nodes, with connections up to a raw distance of 0.95 retained. Red: Query protein sequence (known defluorinating haloacid dehalogenase) used for the initial homology search in the Humgut database. Blue: protein candidates picked for further in vitro experiments.

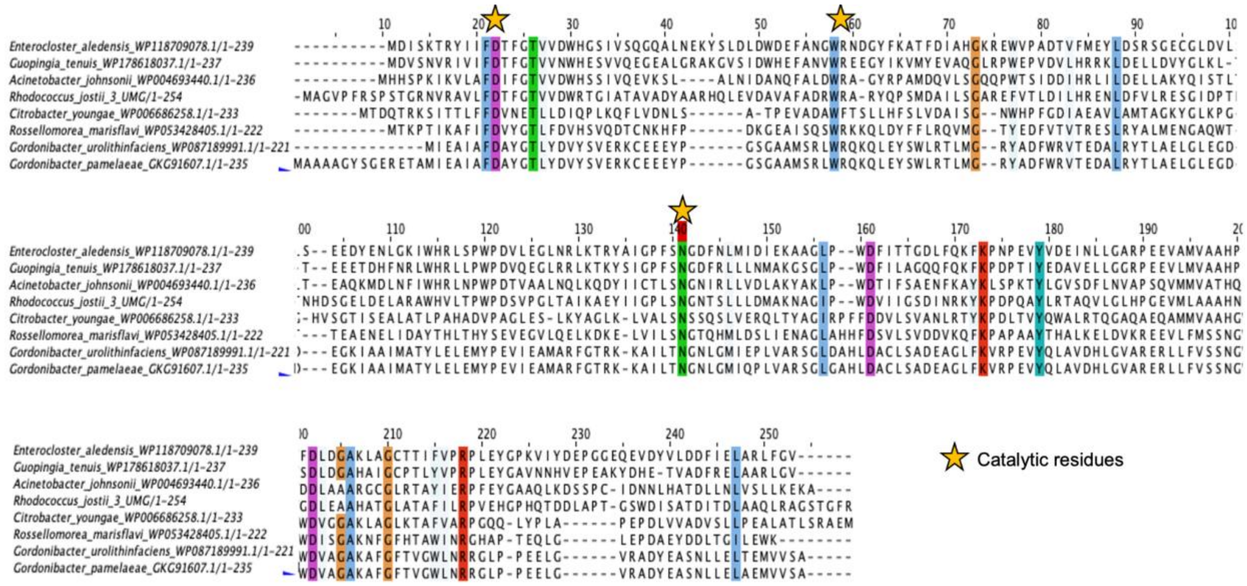

Figure S3: Multiple sequence alignment using Clustal Omega of eight proteins chosen for further in vitro experiments. Amino acid conservation of <95% are indicated by color. The three catalytic amino acids are marked with a star.

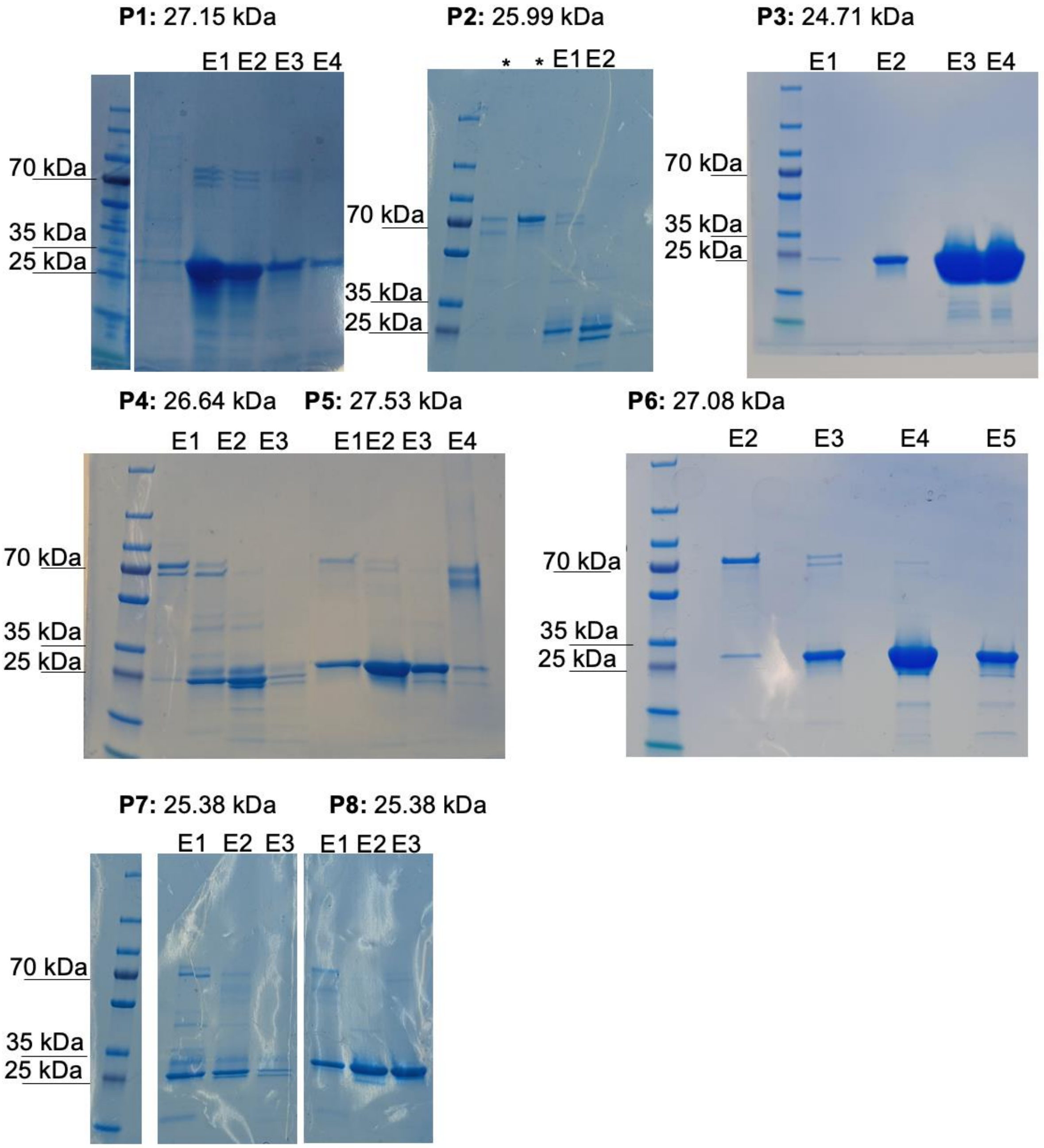

Figure S4: SDS-PAGE gels of the purified proteins P1-P8. *Asterisk indicates proteins not related to P1-P8. Expected sizes in kilodalton (kDa) for each protein are shown relative to the 180 kDa ladder (Thermo Scientific™ PageRuler™).

Protein 1: WP_118709078.1, *Enterocloster aldenensis*

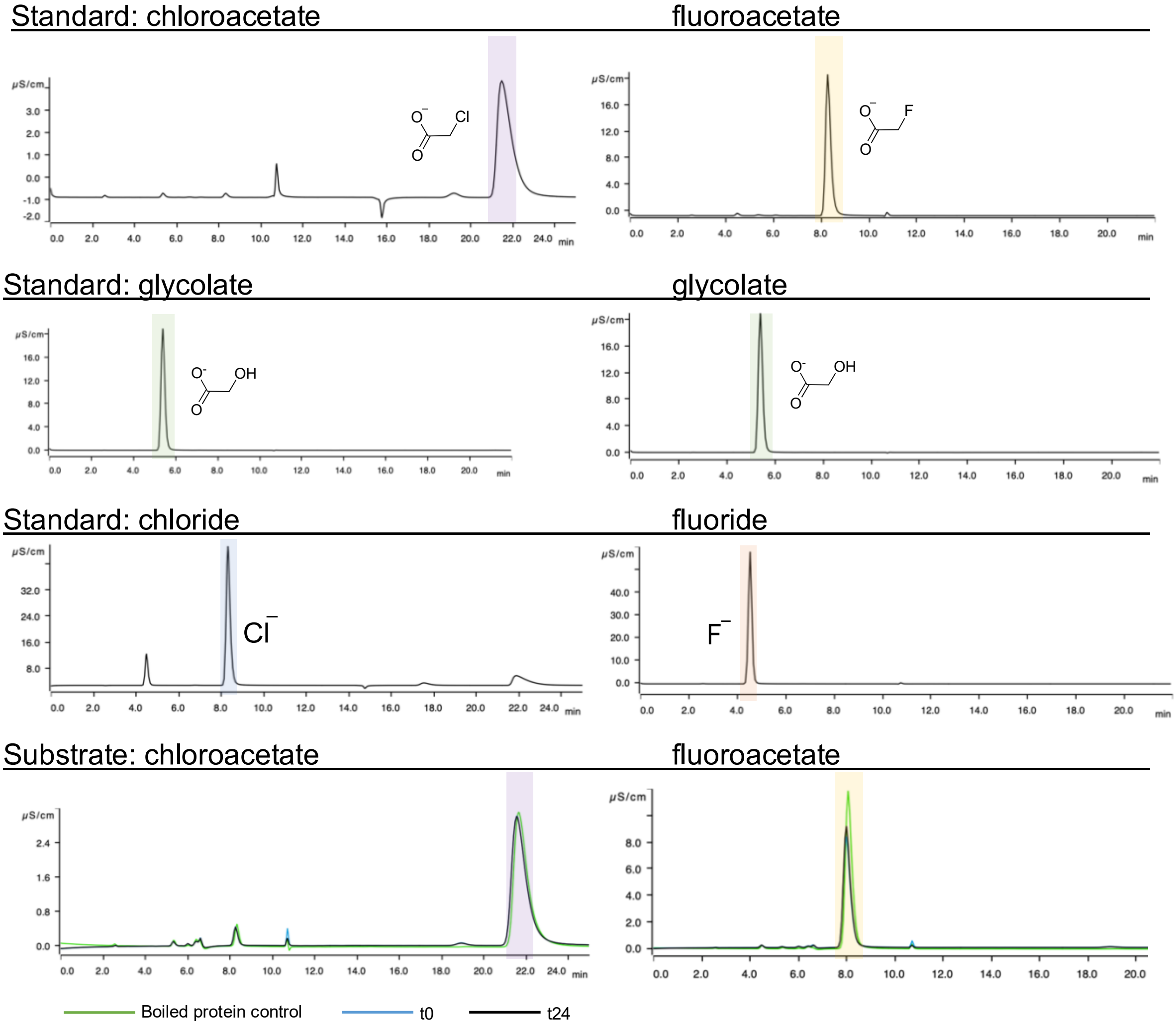

Protein	substrate	% conversion	concentration glycolate [mM]
P1	chloroacetate 2mM	<LOD	<LOD
P1	fluoroacetate 2mM	<LOD	<LOD

Figure S5: Ion chromatography traces of protein P1 (*Enterocloster aldenensis)* with two different substrates.
Green line: Boiled protein control; Blue line: t0h; Black line: t24h. The first three rows show standards. Chloroacetate shaded in purple, fluoroacetate shaded in yellow, glycolate shaded in green, chloride shaded in blue, and fluoride shaded in red. The last row represents the samples incubated with the substrates indicated. The table shows the conversion and measured glycolate concentrations. Glycolate is used for this calculation due to possible interfering chloride contaminations.

Protein 2: GKG91607.1, *Gordonibacter pamelaeae*

Standard: chloroacetate | fluoroacetate

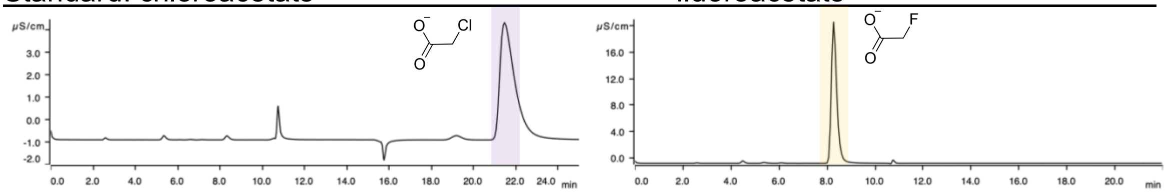

Standard: glycolate | glycolate

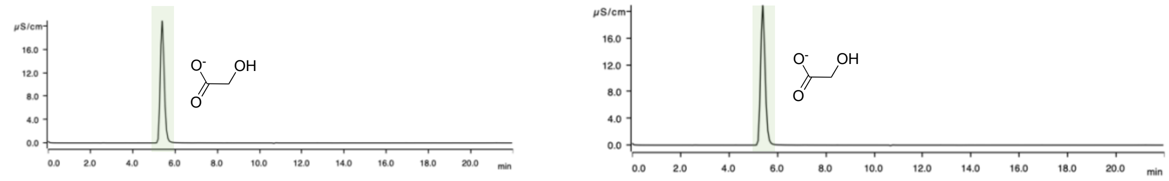

Standard: chloride | fluoride

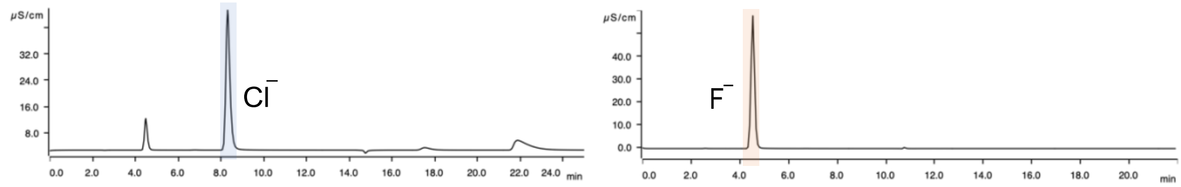

Substrate: chloroacetate | fluoroacetate

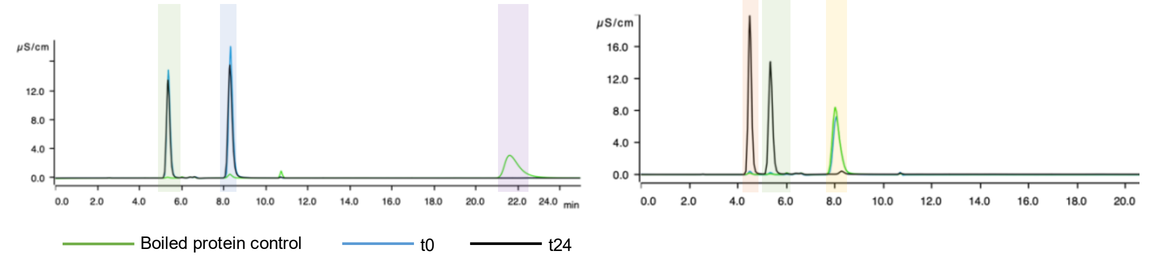

Protein	substrate	% conversion	concentration glycolate [mM]
P2	chloroacetate 2 mM	117	2.34
P2	fluoroacetate 2.18 mM	112	2.46

Substrate: 2-fluoropropionate | 2,2-difluoroacetate

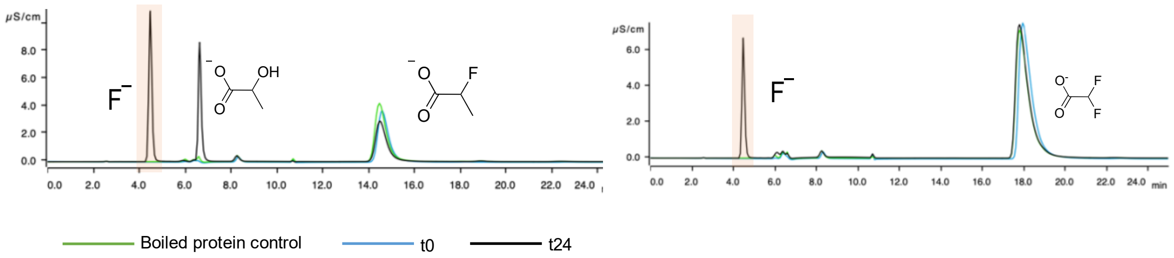

Protein	substrate	% conversion	concentration fluoride [mM]
P2	fluoropropionate 1.7 mM	68.9	1.17
P2	difluoroacetate 3.7 mM	10.85	0.7

Figure S6: Ion chromatography traces of protein P2 (*Gordonibacter pamelaeae*) with four different substrates.

Green line: Boiled protein control; Blue line: t0h; Black line: t24h. The first three rowsshow standards. Chloroacetate shaded in purple, fluoroacetate shaded in yellow, glycolate shaded in green, chloride shaded in blue, and fluoride shaded in red. The last rows represent the samples incubated with the substrates indicated. The table shows the conversion and measured glycolate concentrations. Glycolate is used for this calculation due to possible interfering chloride contaminations. Conversion values >100% are due to instrument variability and slight evaporation which could not be fully avoided.

Protein 3: WP_087189991.1, *Gordonibacter urolithinfaciens*

Standard: chloroacetate fluoroacetate

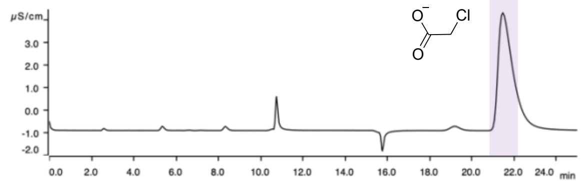

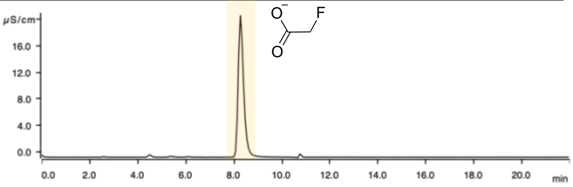

Standard: glycolate glycolate

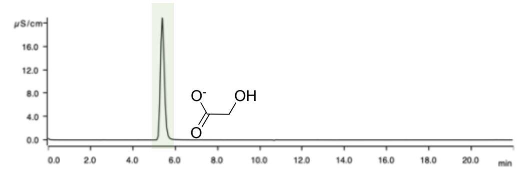

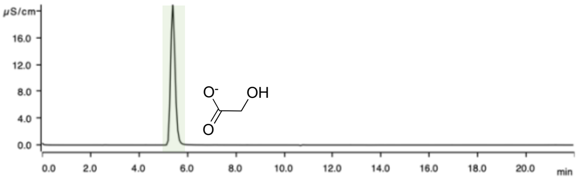

Standard: chloride fluoride

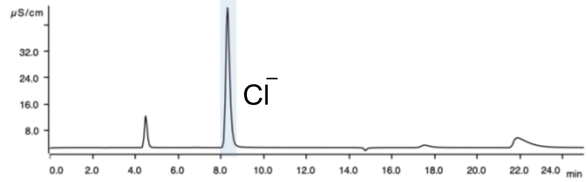

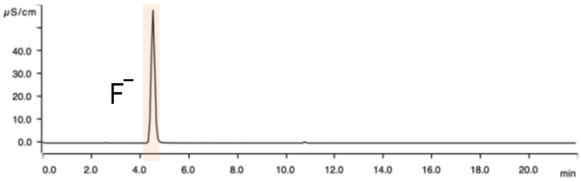

Substrate: chloroacetate fluoroacetate

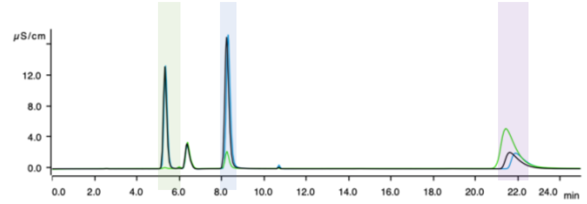

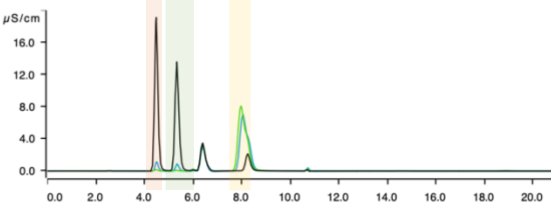

Boiled protein control t0 t24

Protein	substrate	% conversion	concentration glycolate [mM]
P3	chloroacetate 3.4 mM	69.6	2.36
P3	fluoroacetate 2.4 mM	103	2.45

Substrate: 2-fluoropropionate 2,2-difluoroacetate

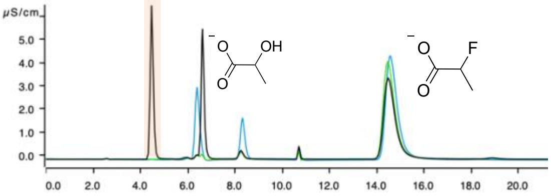

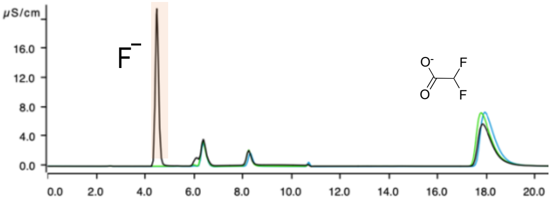

Boiled protein control t0 t24

Protein	substrate	% conversion	concentration fluoride [mM]

P3	fluoropropionate 1.7 mM	45.5	0.77
P3	difluoroacetate 3.8 mM	35	2.63

Figure S7: Ion chromatography traces of protein P3 (*Gordonibacter urolithinfaciens*) with four different substrates. Green line: Boiled protein control; Blue line: t0h; Black line: t24h. The first three rows show standards. Chloroacetate shaded in purple, fluoroacetate shaded in yellow, glycolate shaded in green, chloride shaded in blue, and fluoride shaded in red. The last rows represent the samples incubated with the substrates indicated. The tables show the conversion and measured glycolate concentrations. Glycolate is used for this calculation due to possible interfering chloride contaminations. Conversion values >100% are due to instrument variability and slight evaporation which could not be fully avoided.

Protein 4: WP_004693440.1, *Acinetobacter johnsonii*

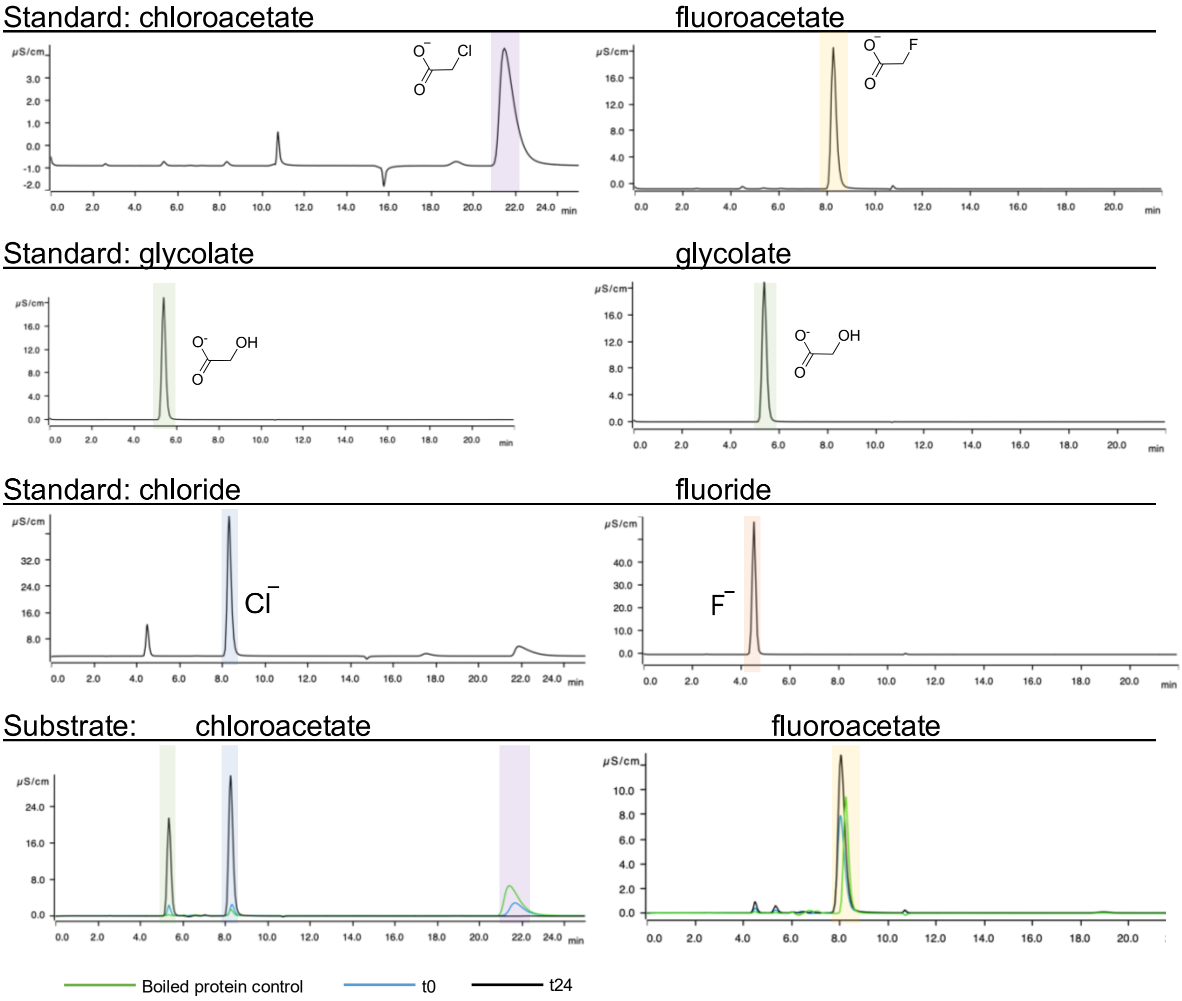

Protein	substrate	% conversion	concentration glycolate [mM]
P4	chloroacetate 4.4 mM	105	4.62
P4	fluoroacetate 2.57 mM	26.2	0.674

Figure S8: Ion chromatography traces of protein P4 (*Acinetobacter johnsonii*) with two substrates. Green line: Boiled protein control; Blue line: t0h; Black line: t24h. The first three rows show standards. Chloroacetate shaded in purple, fluoroacetate shaded in yellow, glycolate shaded in green, chloride shaded in blue, and fluoride shaded in red. The last row represents the samples incubated with the substrates indicate. The table shows the conversion and measured glycolate concentrations. Glycolate is used for this calculation due

to possible interfering chloride contaminations. Conversion values >100% are due to instrument variability and slight evaporation which could not be fully avoided.

Protein 5: 3UMG_1, *Rhodococcus jostii*

Standard: chloroacetate

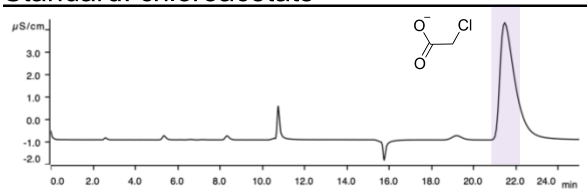

fluoroacetate

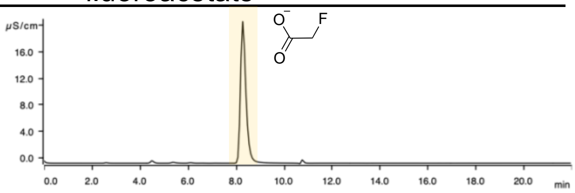

Standard: glycolate

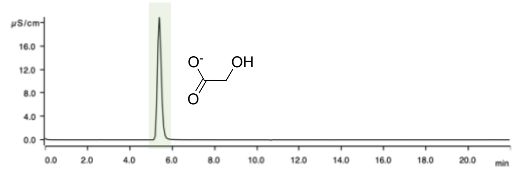

glycolate

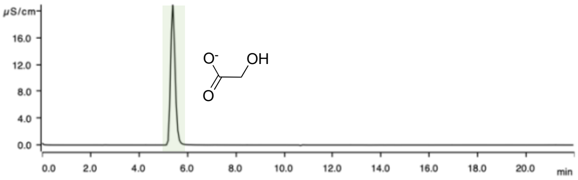

Standard: chloride

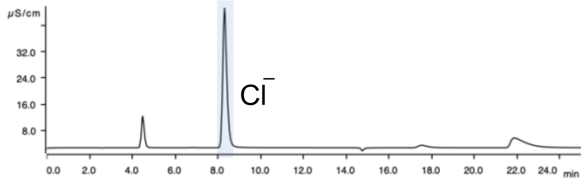

fluoride

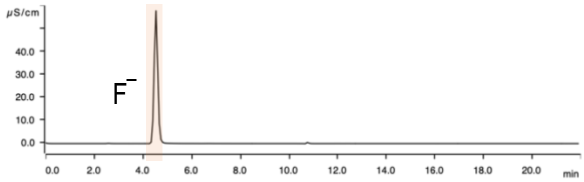

Substrate: chloroacetate

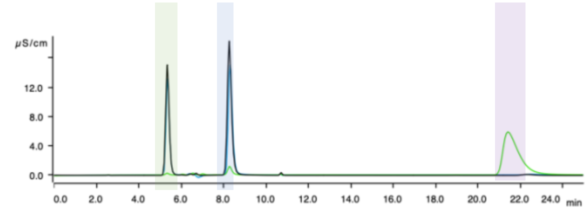

fluoroacetate

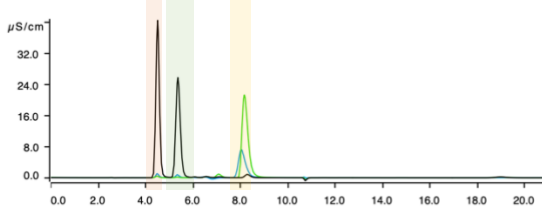

Boiled protein control — t0 — t24

Protein	substrate	% conversion	concentration glycolate [mM]
P5	chloroacetate 3.9 mM	82.7	3.22
P5	fluoroacetate 4.7 mM	79.8	3.78

Substrate: 2-fluoropropionate

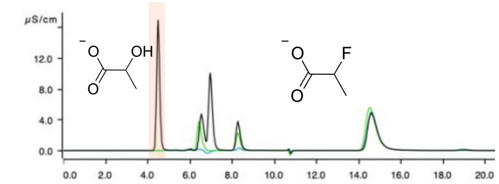

2,2-difluoroacetate

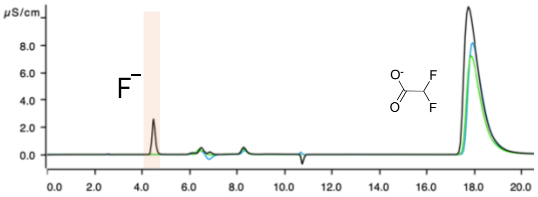

Boiled protein control — t0 — t24

Protein	substrate	% conversion	concentration fluoride [mM]
P5	fluoropropionate 2.35 mM	68.3	1.6
P5	difluoroacetate 3.75 mM	5.22	0.357

Figure S9: Ion chromatography traces of protein P5 (*Rhodococcus jostii* RHA1) with four different substrates. Green line: Boiled protein control; Blue line: t0h; Black line: t24h. The first three row show standards. Chloroacetate shaded in purple, fluoroacetate shaded in yellow, glycolate shaded in green, chloride shaded in blue, and fluoride shaded in red. The last rows represent the samples incubated with the substrates indicated. The table shows the conversion and measured glycolate concentrations. Glycolate is used for this calculation due to possible interfering chloride contaminations.

Protein 6: WP_178618037.1, *Guopingia tenuis*

Standard: chloroacetate

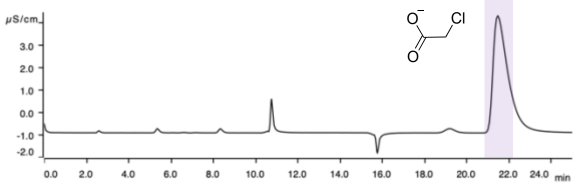

fluoroacetate

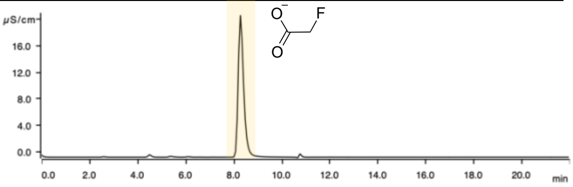

Standard: glycolate

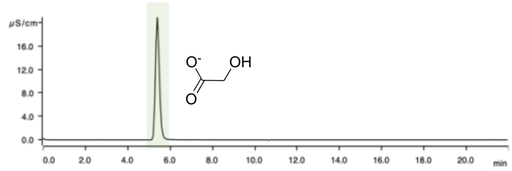

glycolate

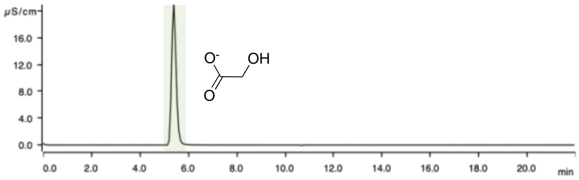

Standard: chloride

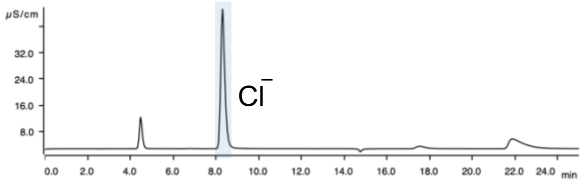

fluoride

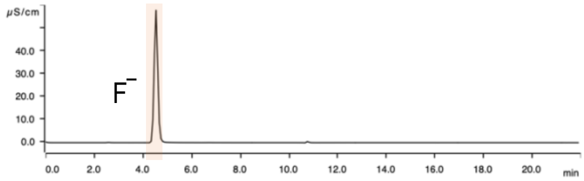

Substrate: chloroacetate

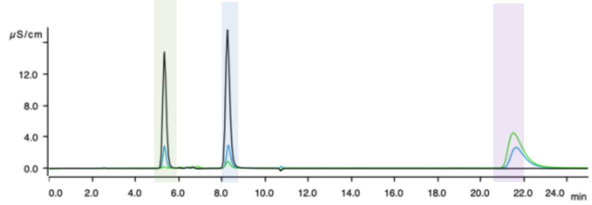

fluoroacetate

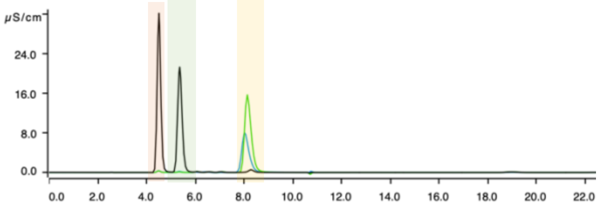

Boiled protein control t0 t24

Protein	substrate	% conversion	concentration glycolate [mM]
P6	chloroacetate 2.9 mM	121	3.35
P6	fluoroacetate 3.4 mM	101	3.70

Substrate: fluoro-beta-alanine

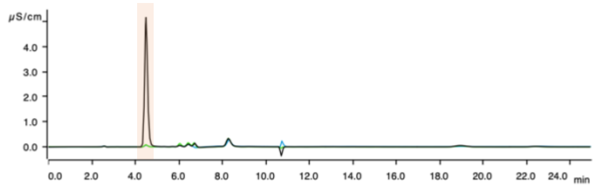

Boiled protein control t0 t24

Protein	substrate	% conversion	concentration fluoride [mM]
P6	Fluoro-beta-alanine 2 mM	36.06	0.72

Figure S10: Ion chromatography traces of protein P6 (*Guopingia tenuis)* with different three substrates. Green line: Boiled protein control; Blue line: t0h; Black line: t24h. The first three rows show standards. Chloroacetate shaded in purple, fluoroacetate shaded in yellow, glycolate shaded in green, chloride shaded in blue and fluoride shaded in red. The last rows represent the samples incubated with the substrates indicated. The tables show the conversion and measured glycolate concentrations. Glycolate is used for this calculation due to possible interfering chloride contaminations. Conversion values >100% are due to instrument variability and slight evaporation which could not be fully avoided.

Protein 7: WP_006686258.1, *Citrobacter youngae*

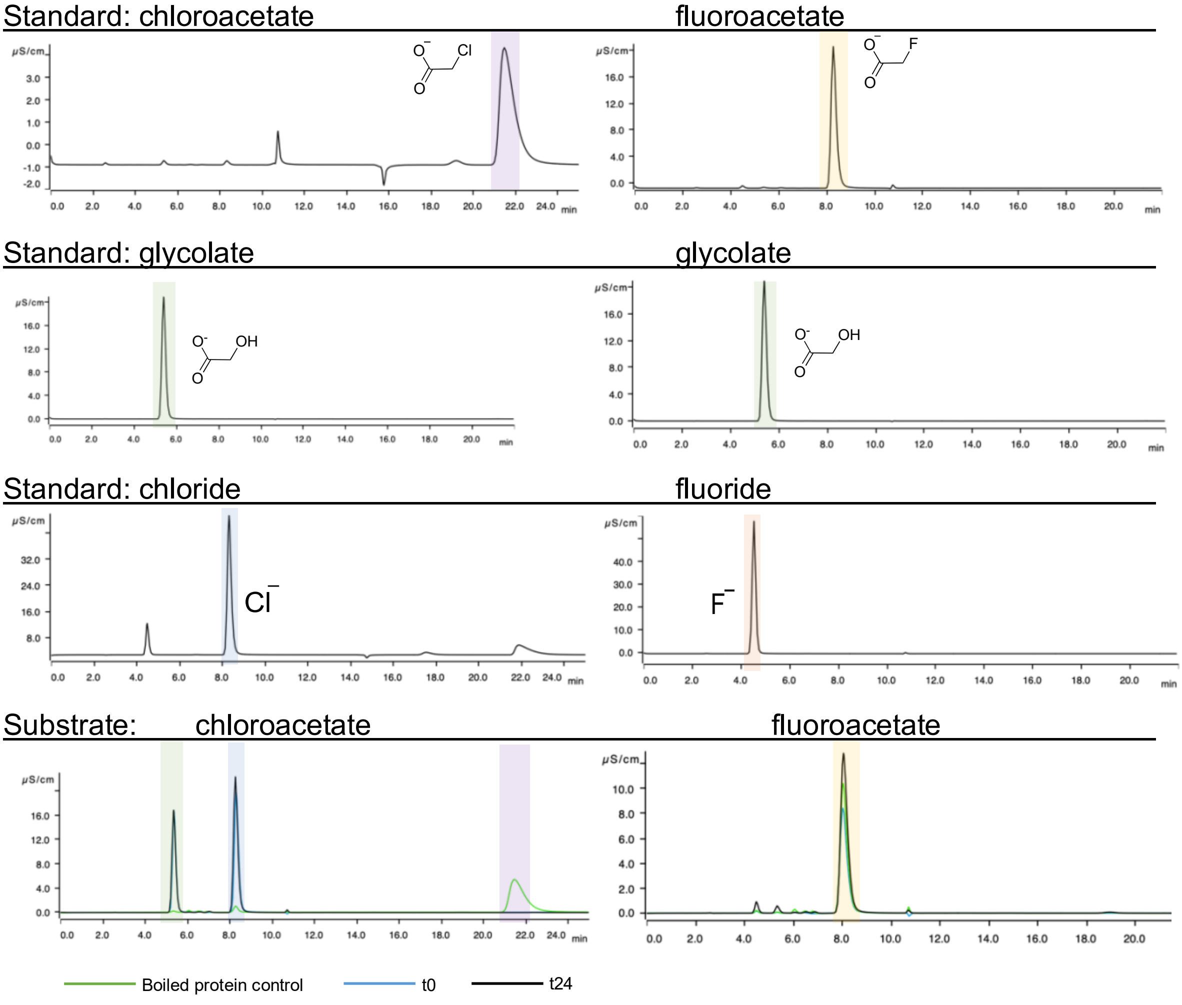

Protein	substrate	% conversion	concentration glycolate [mM]
P7	chloroacetate 3.5 mM	92.6	3.24
P7	fluoroacetate 2.7 mM	4.77	0.126

Figure S11: Ion chromatography traces of protein WP_006686258.1 (*Citrobacter youngae*) with two different substrates. Green line: Boiled protein control; Blue line: t0h; Black line: t24h. The first three rows show standards. Chloroacetate shaded in purple, fluoroacetate shaded in yellow, glycolate shaded in green, chloride shaded in blue, and fluoride shaded in red. The last row represent the samples incubated with the substrates indicated. The table shows the

conversion and measured glycolate concentrations. Glycolate is used for this calculation due to possible interfering chloride contaminations.

Protein 8: WP_053428405.1, *Rossellomorea marisflavi*

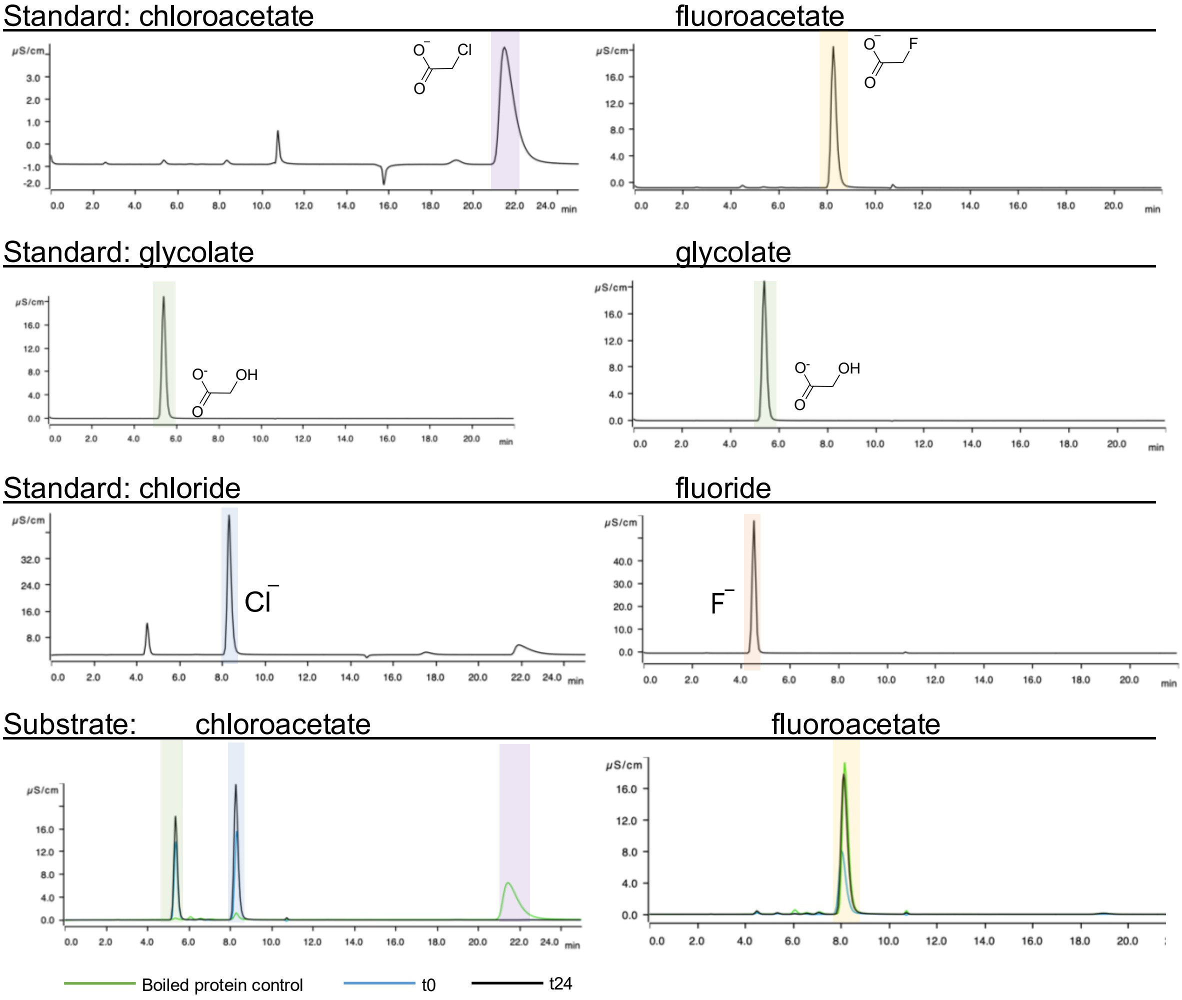

Protein	substrate	% conversion	concentration glycolate [mM]
P8	chloroacetate 4.3 mM	89.5	3.84
P8	fluoroacetate 4.3 mM	0.8	0.03

Figure S12: Ion chromatography traces of protein P8 (*Rossellomorea marisflavi)* with two different substrates. Green line: Boiled protein control; Blue line: t0h; Black line: t24h. The first three rows show standards. Chloroacetate shaded in purple, fluoroacetate shaded in yellow, glycolate shaded in green, chloride shaded in blue, and fluoride shaded in red. The last row represent the samples incubated with the substrates indicated. The table shows the conversion and measured glycolate concentrations. Glycolate is used for this calculation due to possible interfering chloride contaminations.

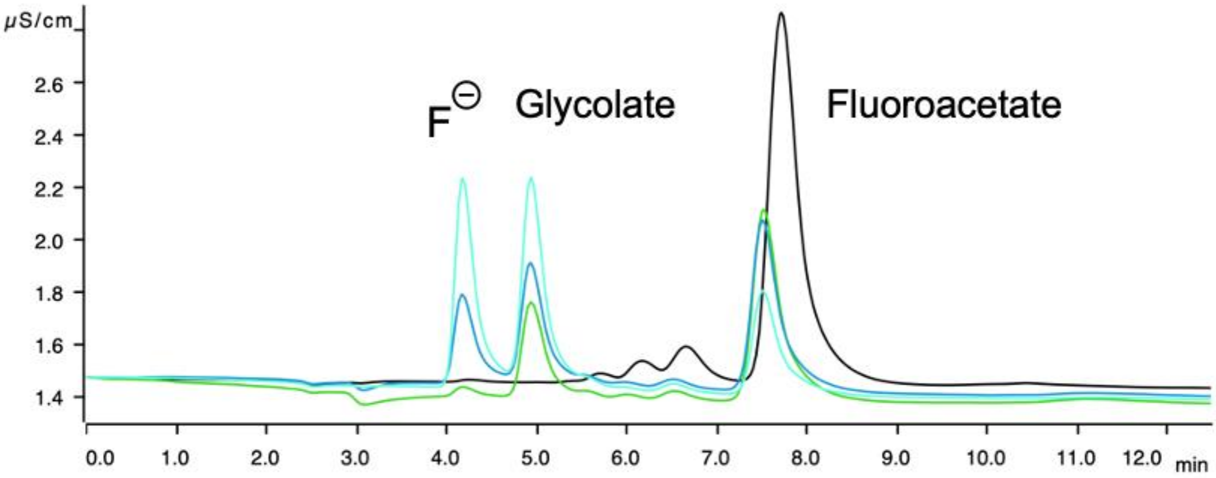

Figure S13: Ion chromatography traces of protein P3 (*Gordonibacter urolithinfaciens*) incubated anaerobically with fluoroacetate. Black: control; Green t1h; Blue: t3h; ligth blue: t20h.

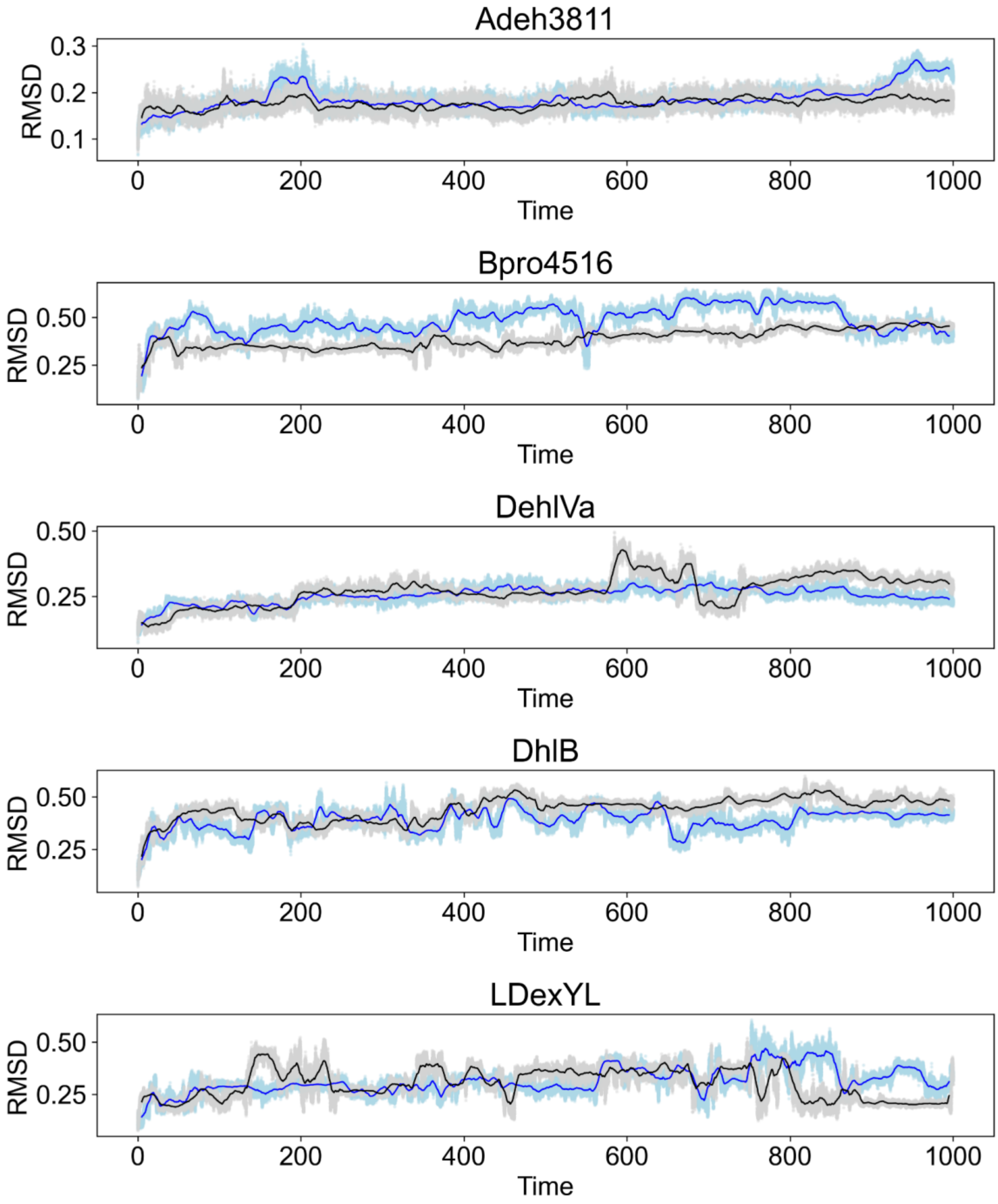
Adeh3811
RMSD
0.3
0.2
0.1
0
200
400
600
800
1000
Time
Bpro4516
RMSD
0.50
0.25
0
200
400
600
800
1000
Time
DehIVa
RMSD
0.50
0.25
0
200
400
600
800
1000
Time
DhlB
RMSD
0.50
0.25
0
200
400
600
800
1000
Time
LDexYL
RMSD
0.50
0.25
0
200
400
600
800
1000
Time

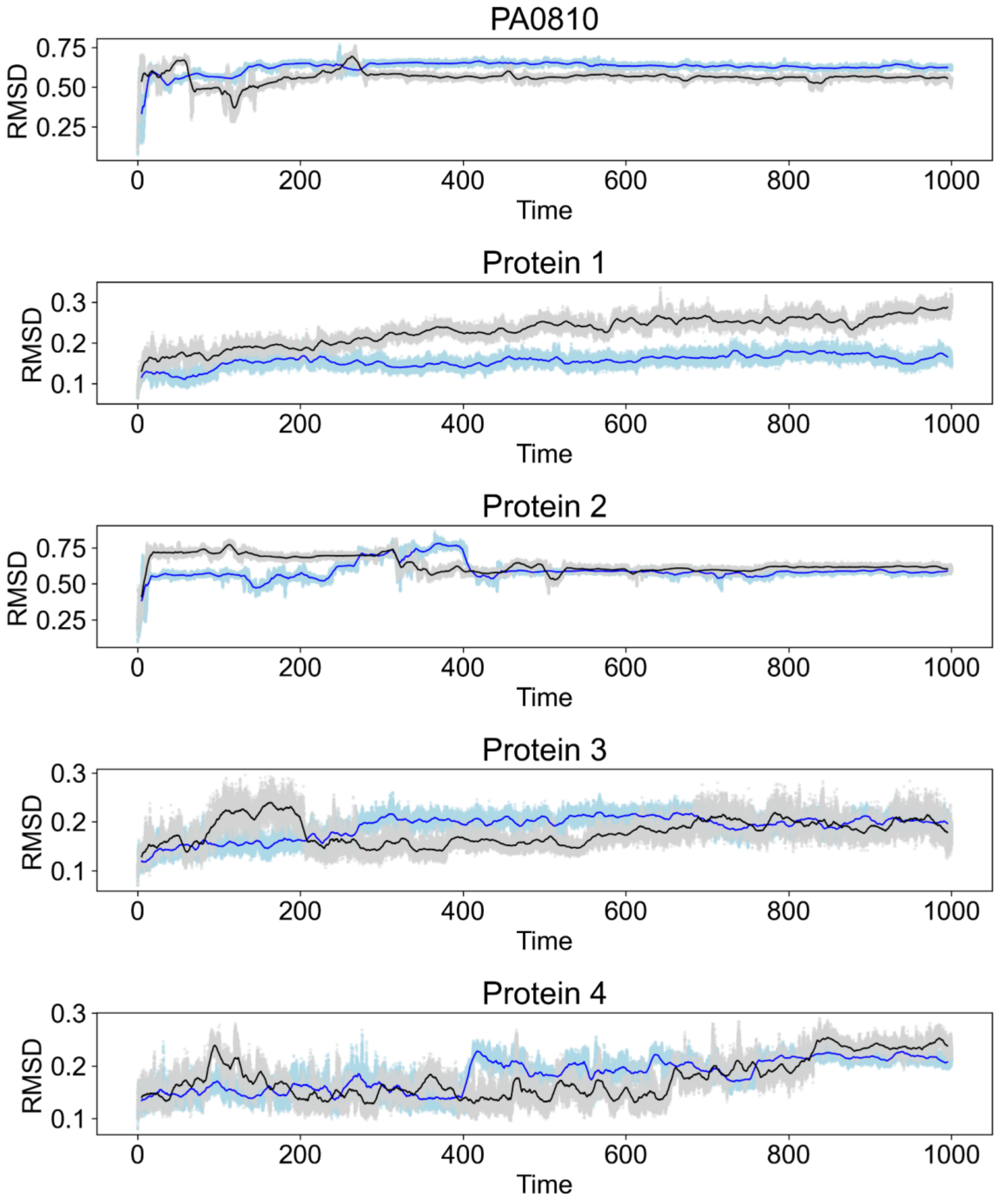
PA0810
RMSD
0.75
0.50
0.25
0
200
400
600
800
1000
Time
Protein 1
RMSD
0.3
0.2
0.1
0
200
400
600
800
1000
Time
Protein 2
RMSD
0.75
0.50
0.25
0
200
400
600
800
1000
Time
Protein 3
RMSD
0.3
0.2
0.1
0
200
400
600
800
1000
Time
Protein 4
RMSD
0.3
0.2
0.1
0
200
400
600
800
1000
Time

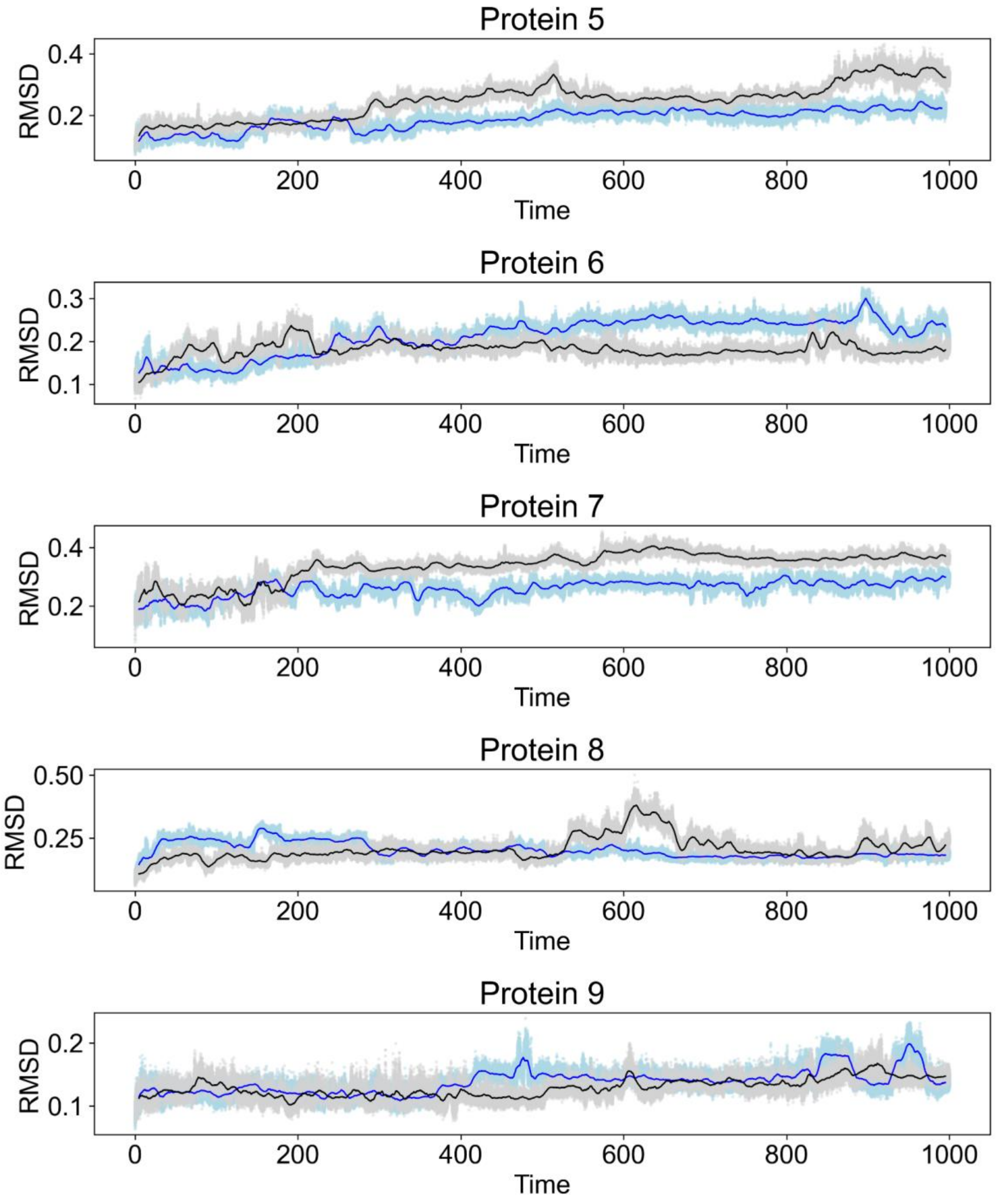

Protein 5
RMSD
0.4
0.2
0
200
400
600
800
1000
Time
Protein 6
RMSD
0.3
0.2
0.1
0
200
400
600
800
1000
Time
Protein 7
RMSD
0.4
0.2
0
200
400
600
800
1000
Time
Protein 8
RMSD
0.50
0.25
0
200
400
600
800
1000
Time
Protein 9
RMSD
0.2
0.1
0
200
400
600
800
1000
Time

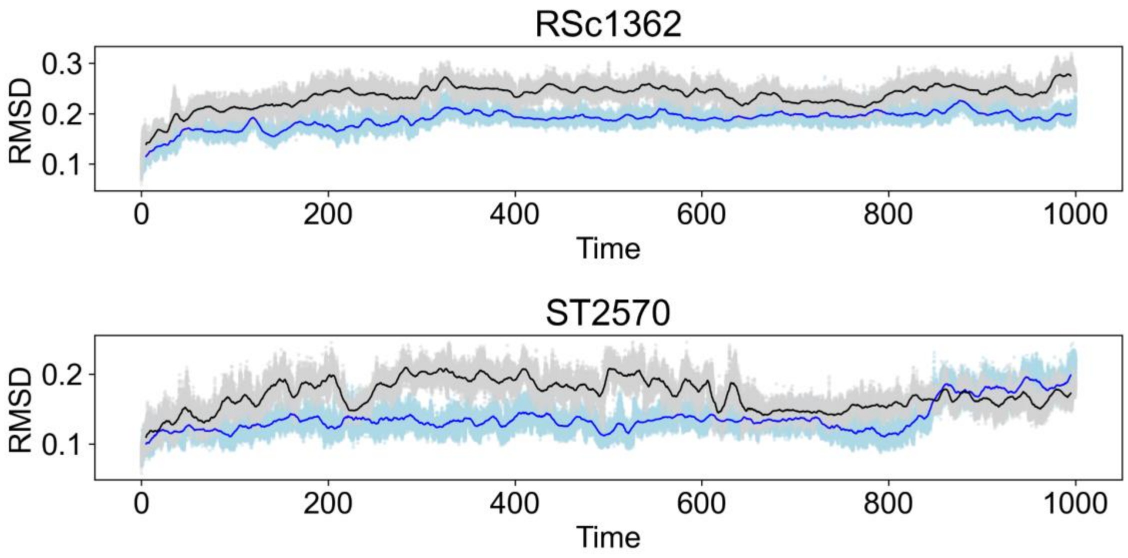

Figure S14: Root Mean Square Deviation (RMSD, in nm) files from molecular dynamic simulations. Two independent replicates with rolling averages of 1 ns are indicated as black and blue lines and standard deviations of the 1 ns window are represented by the gray/light blue shading. X-axis in ns, full simulation time 1µs. Black: replicate 1; Blue: replicate 2;

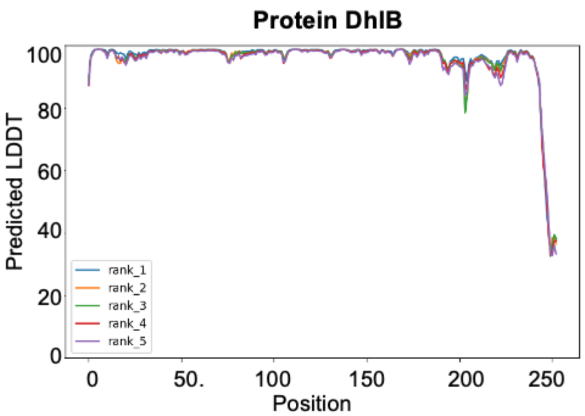
Protein DhlB
Predicted LDDT
Position
0 50. 100 150 200 250
0 20 40 60 80 100
rank_1
rank_2
rank_3
rank_4
rank_5

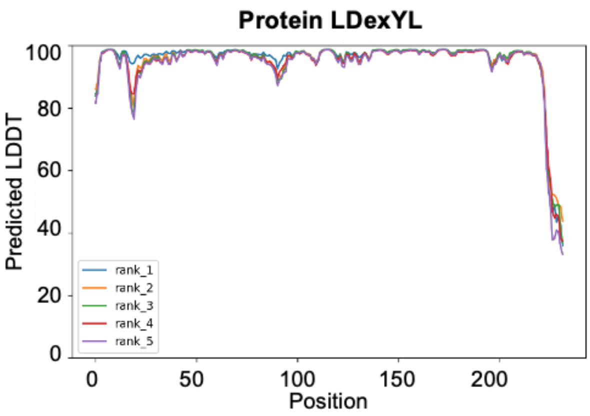
Protein LDexYL
Predicted LDDT
Position
0 50 100 150 200
0 20 40 60 80 100
rank_1
rank_2
rank_3
rank_4
rank_5

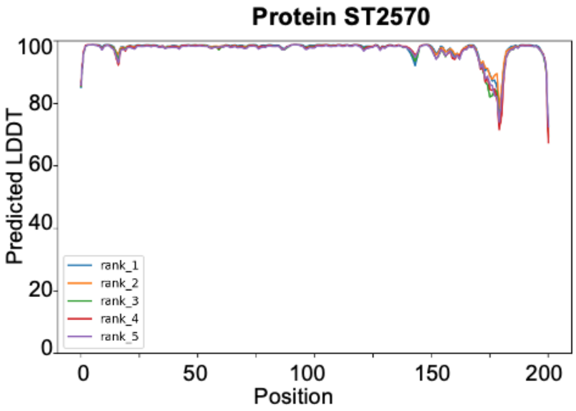
Protein ST2570
Predicted LDDT
Position
0 50 100 150 200
0 20 40 60 80 100
rank_1
rank_2
rank_3
rank_4
rank_5

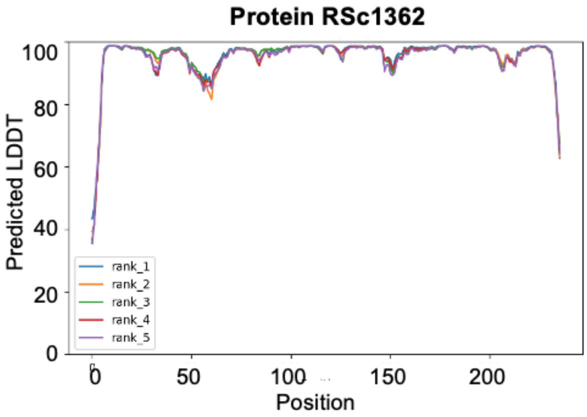
Protein RSc1362
Predicted LDDT
Position
0 50 100 150 200
0 20 40 60 80 100
rank_1
rank_2
rank_3
rank_4
rank_5

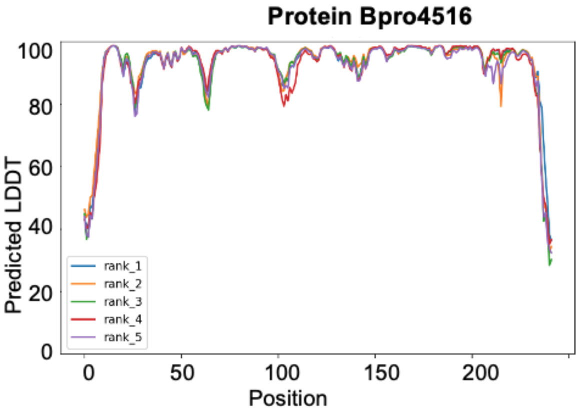
Protein Bpro4516
Predicted LDDT
Position
0 50 100 150 200
0 20 40 60 80 100
rank_1
rank_2
rank_3
rank_4
rank_5

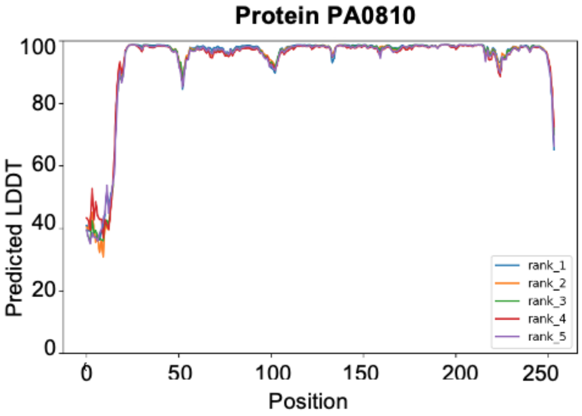
Protein PA0810
Predicted LDDT
Position
0 50 100 150 200 250
0 20 40 60 80 100
rank_1
rank_2
rank_3
rank_4
rank_5

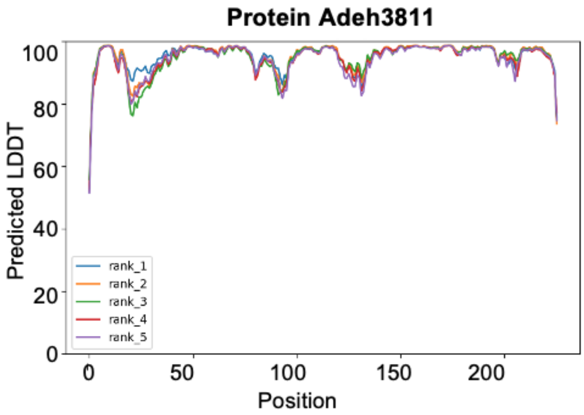
Protein Adeh3811
Predicted LDDT
Position
0 50 100 150 200
0 20 40 60 80 100
rank_1
rank_2
rank_3
rank_4
rank_5

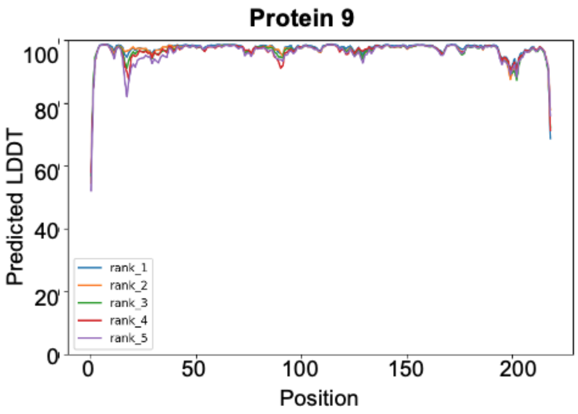
Protein 9
Predicted LDDT
Position
0 50 100 150 200
0 20 40 60 80 100
rank_1
rank_2
rank_3
rank_4
rank_5

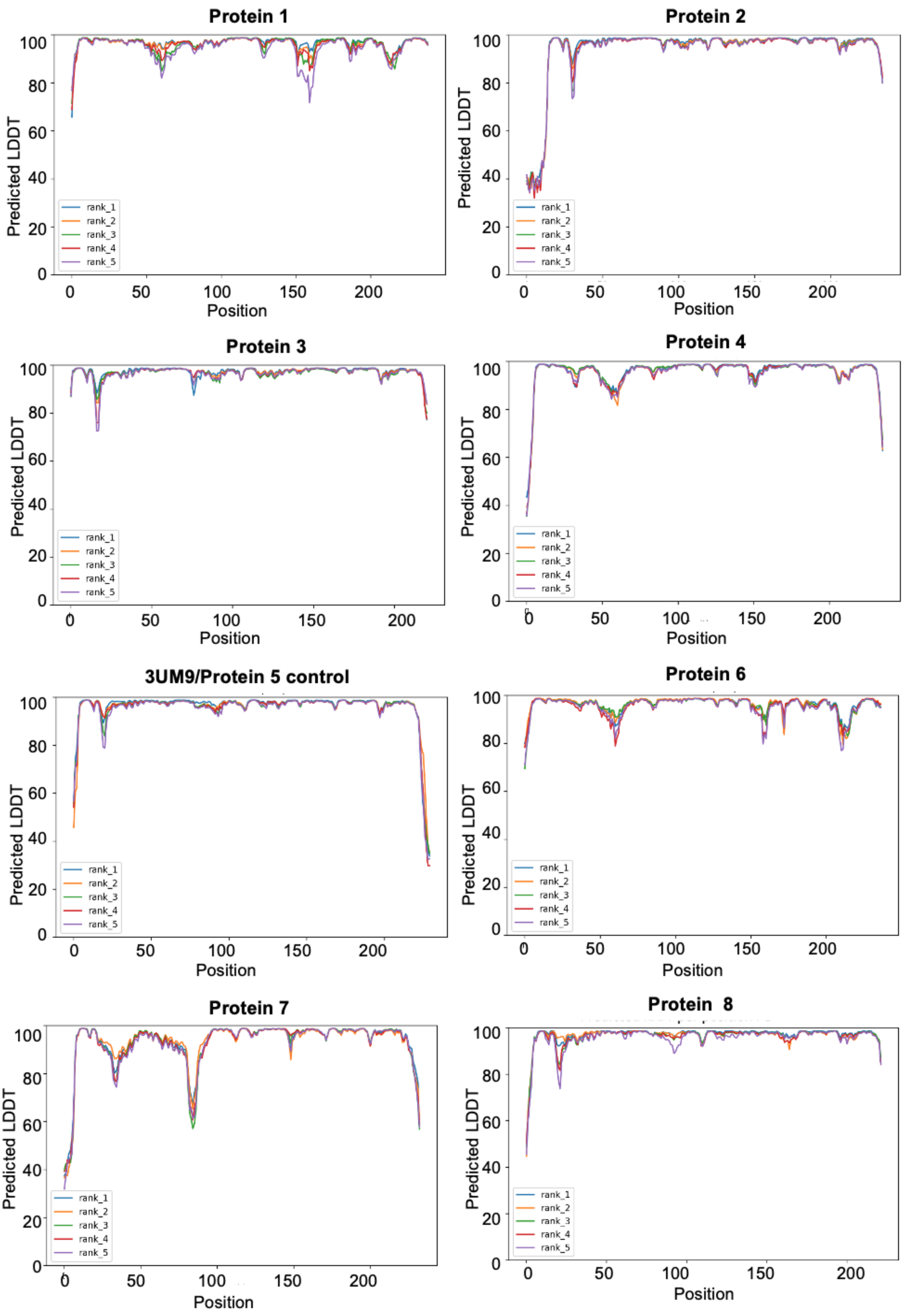

Figure S15: Predicted Local Difference Distance Test (pLDDT) of AlphaFold models used for MD simulations. A high pLDDT value corresponds to the confidence of the model at the specific amino acid position. All models are available in the Github repository: https://github.com/MSM-group/gut_microbe_defluorination_paper/

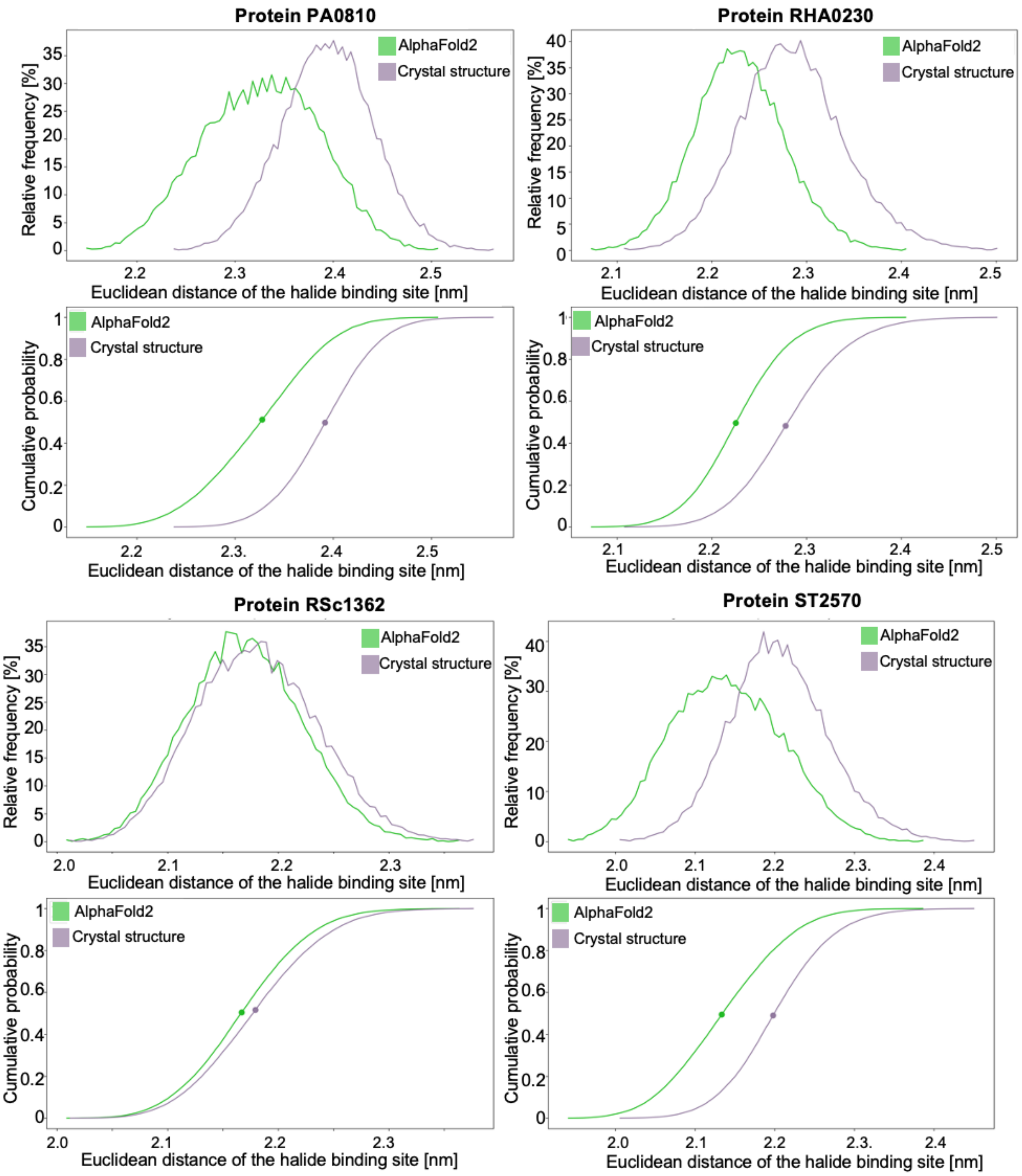

Figure S16: Molecular dynamics (MD) simulations of X-ray crystal structure compared to AlphaFold protein structures of identical proteins used in this study. The top graph of each protein represents the relative frequency of Euclidean distances in the halide binding site. The lower graph is representing the cumulative probability with the point showing the middle. Green: AlphaFold2 model; Purple: crystal structure

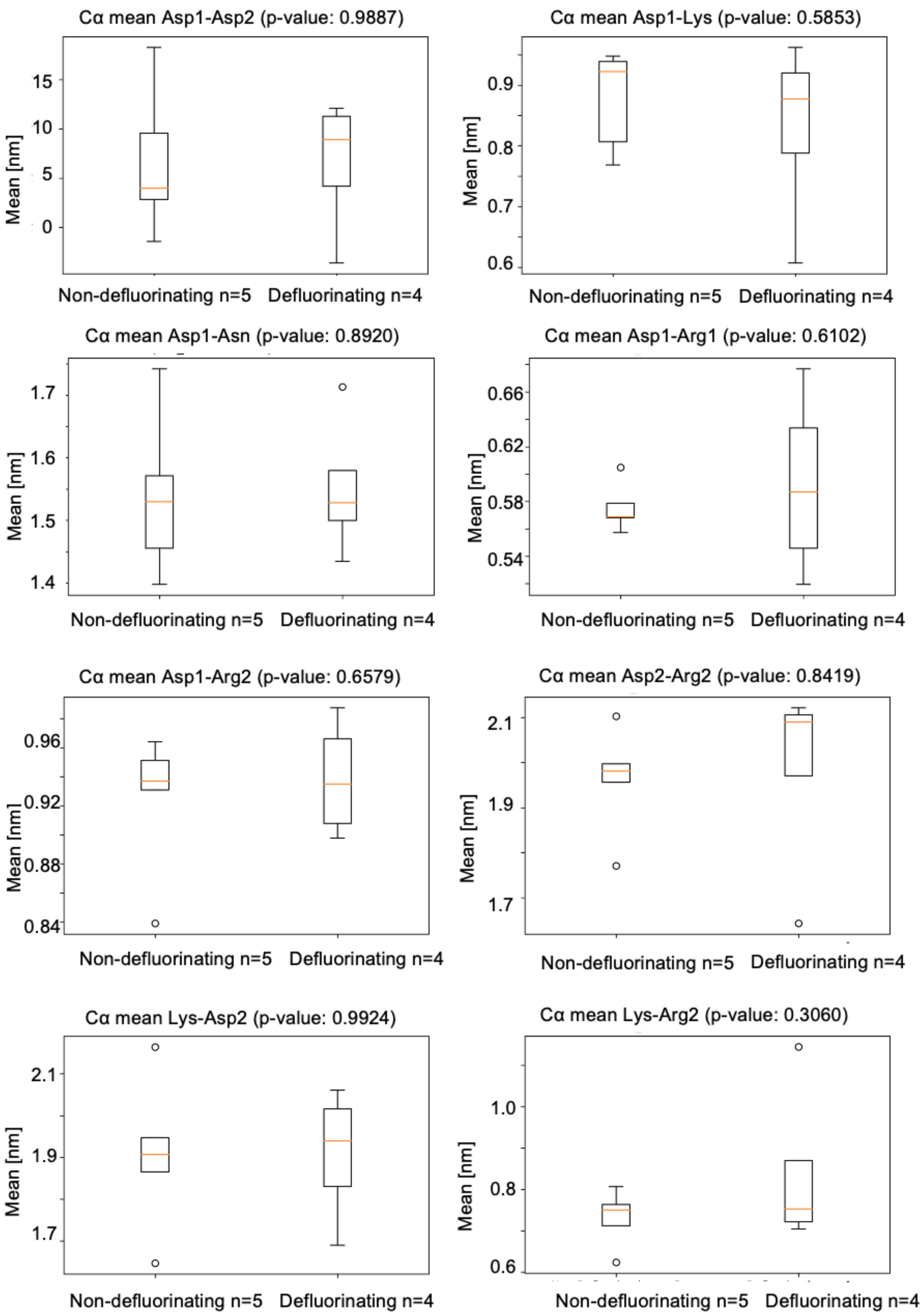
Cα mean Asp1-Asp2 (p-value: 0.9887)
Mean [nm]
15
10
5
0
Non-defluorinating n=5
Defluorinating n=4
Cα mean Asp1-Lys (p-value: 0.5853)
Mean [nm]
0.9
0.8
0.7
0.6
Non-defluorinating n=5
Defluorinating n=4
Cα mean Asp1-Asn (p-value: 0.8920)
Mean [nm]
1.7
1.6
1.5
1.4
Non-defluorinating n=5
Defluorinating n=4
Cα mean Asp1-Arg1 (p-value: 0.6102)
Mean [nm]
0.66
0.62
0.58
0.54
Non-defluorinating n=5
Defluorinating n=4
Cα mean Asp1-Arg2 (p-value: 0.6579)
Mean [nm]
0.96
0.92
0.88
0.84
Non-defluorinating n=5
Defluorinating n=4
Cα mean Asp2-Arg2 (p-value: 0.8419)
Mean [nm]
2.1
1.9
1.7
Non-defluorinating n=5
Defluorinating n=4
Cα mean Lys-Asp2 (p-value: 0.9924)
Mean [nm]
2.1
1.9
1.7
Non-defluorinating n=5
Defluorinating n=4
Cα mean Lys-Arg2 (p-value: 0.3060)
Mean [nm]
1.0
0.8
0.6
Non-defluorinating n=5
Defluorinating n=4

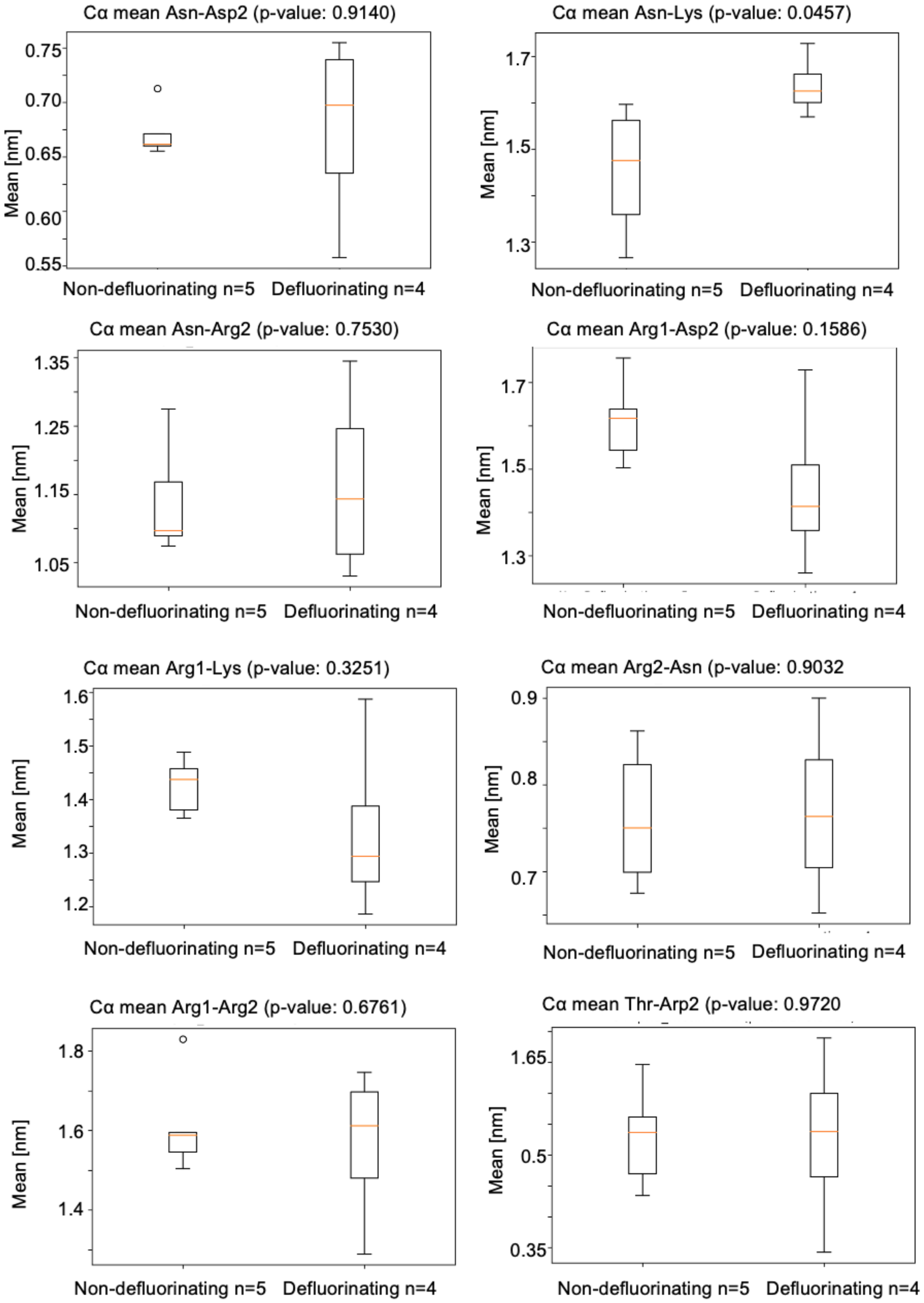
Cα mean Asn-Asp2 (p-value: 0.9140)
Mean [nm]
0.75
0.70
0.65
0.60
0.55
Non-defluorinating n=5
Defluorinating n=4
Cα mean Asn-Lys (p-value: 0.0457)
Mean [nm]
1.7
1.5
1.3
Non-defluorinating n=5
Defluorinating n=4
Cα mean Asn-Arg2 (p-value: 0.7530)
Mean [nm]
1.35
1.25
1.15
1.05
Non-defluorinating n=5
Defluorinating n=4
Cα mean Arg1-Asp2 (p-value: 0.1586)
Mean [nm]
1.7
1.5
1.3
Non-defluorinating n=5
Defluorinating n=4
Cα mean Arg1-Lys (p-value: 0.3251)
Mean [nm]
1.6
1.5
1.4
1.3
1.2
Non-defluorinating n=5
Defluorinating n=4
Cα mean Arg2-Asn (p-value: 0.9032
Mean [nm]
0.9
0.8
0.7
Non-defluorinating n=5
Defluorinating n=4
Cα mean Arg1-Arg2 (p-value: 0.6761)
Mean [nm]
1.8
1.6
1.4
Non-defluorinating n=5
Defluorinating n=4
Cα mean Thr-Arp2 (p-value: 0.9720
Mean [nm]
1.65
0.5
0.35
Non-defluorinating n=5
Defluorinating n=4

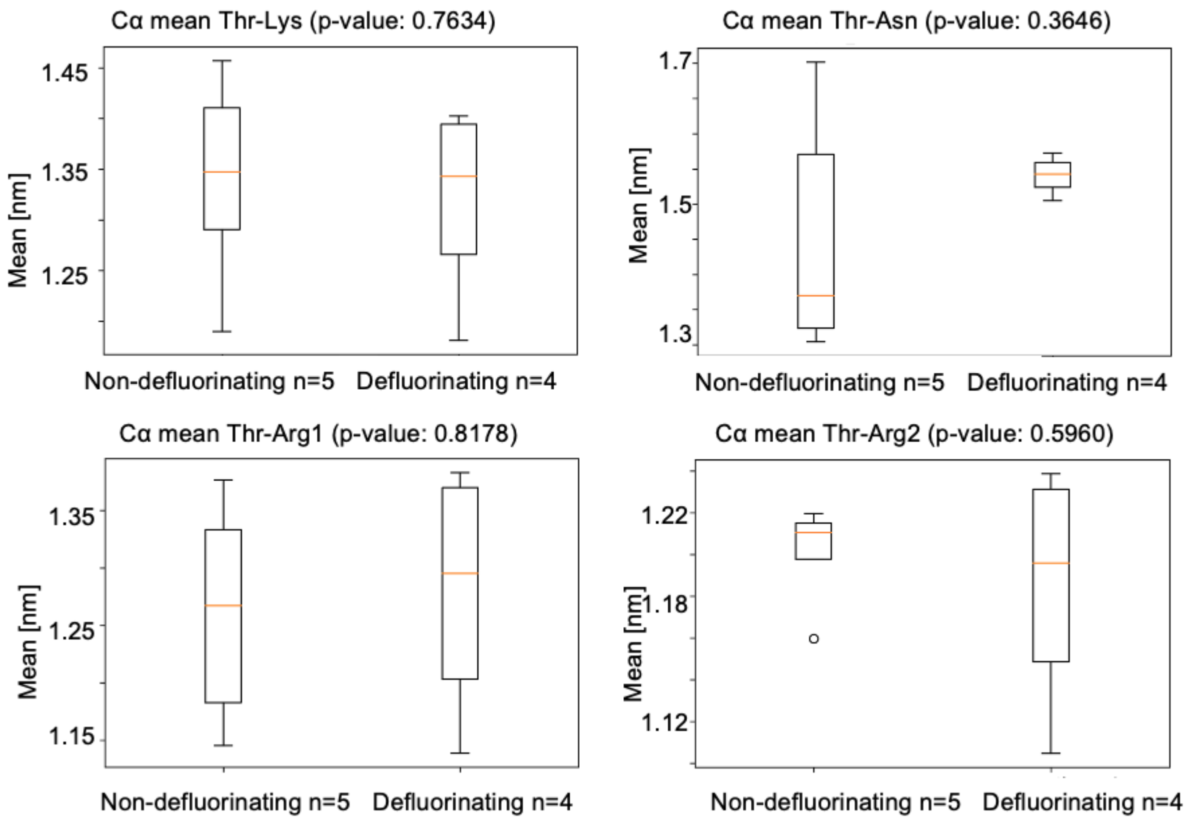

Figure S17: Boxplots of the mean distances between conserved residues during a 1 μs MD simulation and p-value of the two-sided pairwise t-tests of these distances between the defluorinating and non-defluorinating haloacid dehalogenases.

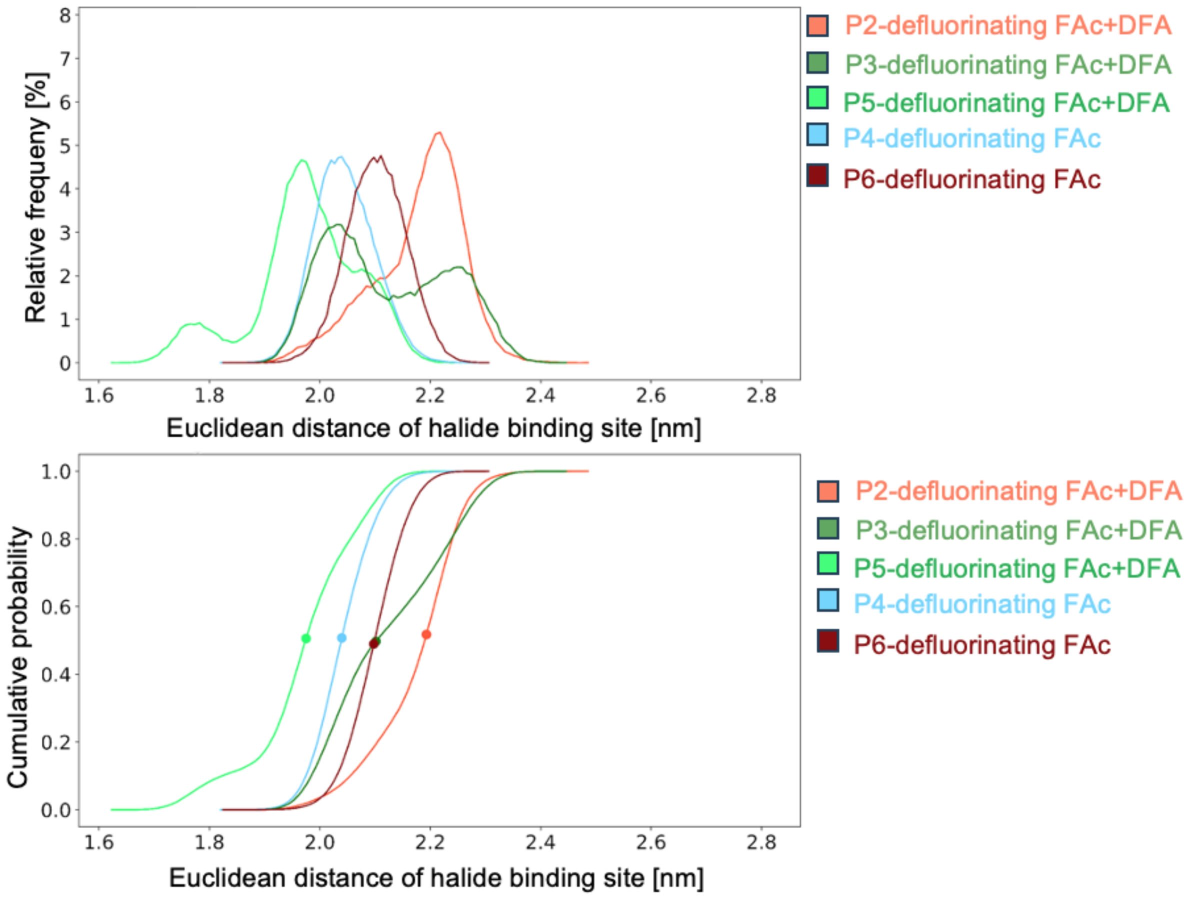

Figure S18: **Halide binding site (HBS) change during MD simulations** comparing HADs able to defluorinate fluoroacetate (FAc) and difluoroacetate (DFA) with HADs only defluorinating FAc. The top graph represents the relative frequency of Euclidean distances in the halide binding site. The lower graph is representing the cumulative probability with the point showing the midpoint.

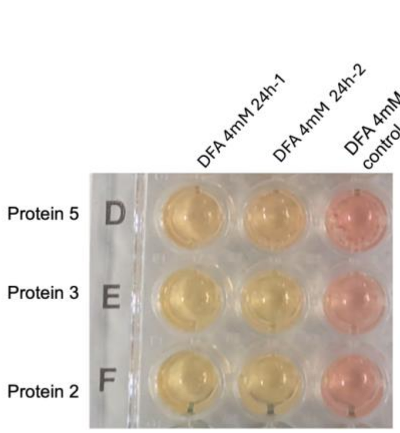

Conversion of DFA (4 mM) colorimetric assay						
	24h-1		24h-2		24h-boiled protein control	
	F^- conc.	conv.	F^- conc.	conv.	F^- conc.	conv.
Protein 5	602 μM	7.7 %	546 μM	7 %	98 μM	1.19 %
Protein 3	861 μM	10.5 %	735 μM	9.31 %	147 μM	1.82 %
Protein 2	777 μM	9.45 %	672 μM	8.4 %	126 μM	1.61 %

Figure S19: **Fluoride concentration and difluoroacetate conversion** for proteins 2,3 and 5 tested with the colorimetric assay. The difluoroacetate standard provided by the manufacturer has a purity of >98%.

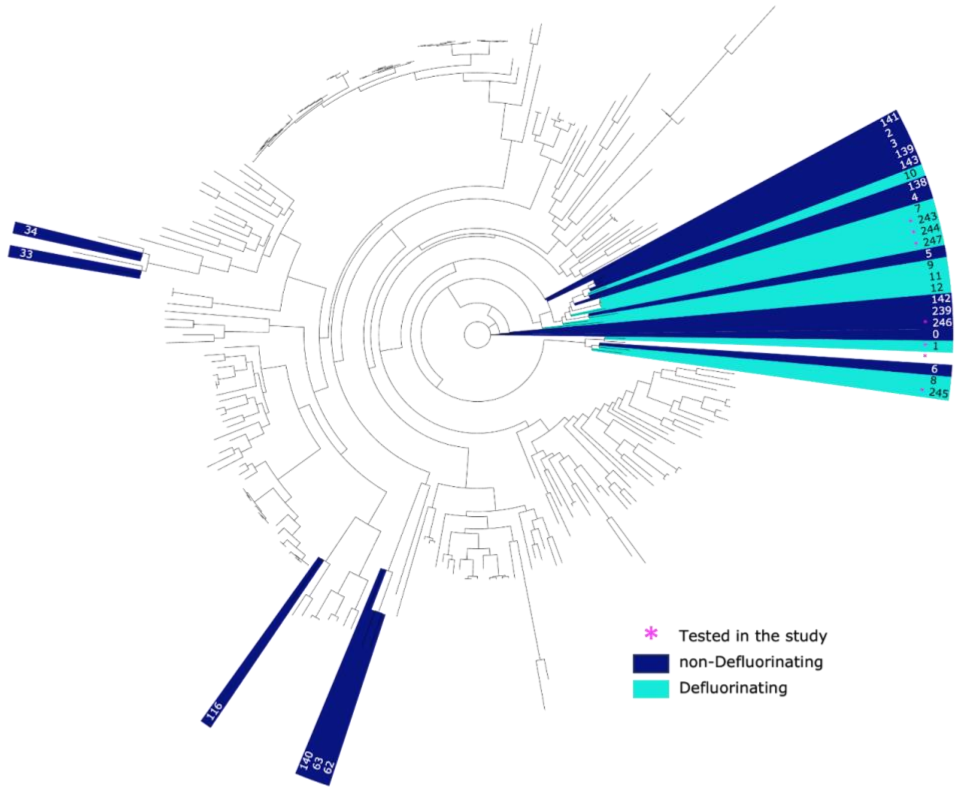

Figure S20: Maximum-likelihood phylogenetic analysis of HAD-like superfamily enzymes. Colored leaves are the subset of the experimentally confirmed dehalogenating enzymes relative to HAD-like superfamily enzymes performing other functions (e.g., phosphoryl transfer). The different colors correspond to the defluorination properties. Our data set contained 248 homologous HAD-like enzymes, 29 of which were experimentally confirmed dehalogenases. Among the 29 dehalogenases, ten were confirmed to defluorinate (four newly identified in this study, six from literature). The indices in the tree correspond to the indices in supplementary table S9 with the full metadata of the HAD-like dataset. Enzymes that have been tested in this study are marked with an asterisk.

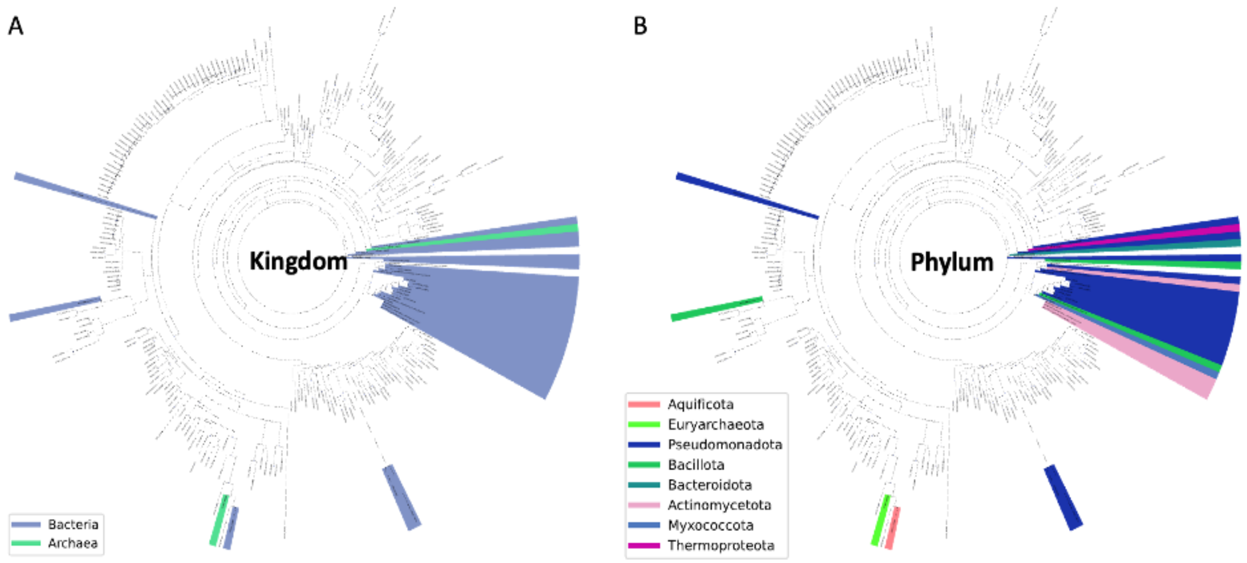

Figure S21: Dehalogenase subset location in the HAD-like family phylogenetic tree and their taxonomy. A) Colored leaves correspond to the dehalogenating enzymes subset and show that they are sourced solely from Bacteria and Archaea. B) The corresponding phyla of the dehalogenase subset is shown in A).

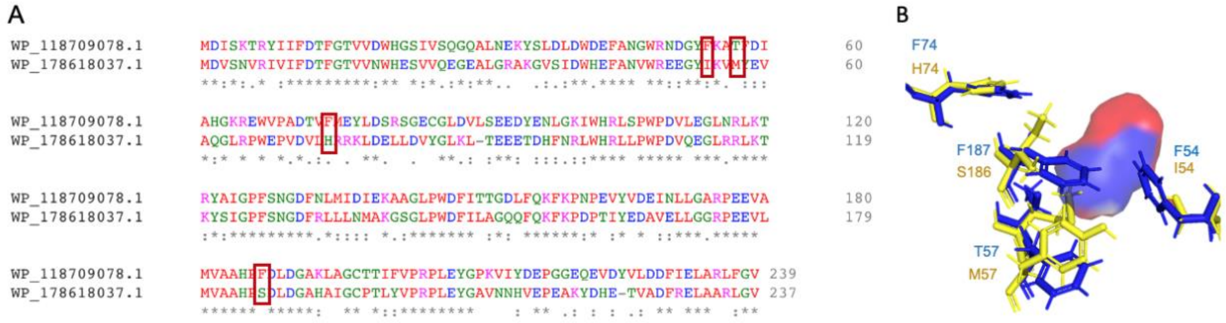

Figure S21: A) Sequence alignment of the defluorinating P6 and the non defluorinating protein P1. Red boxes: the only amino acids different and located in the active center. B) location of the marked amino acids with the active pocket. Blue (WP_118709078.1) = P1, Yellow (WP_178618037.1) = P6.

Substrate: chloroacetate fluoroacetate

P1 - F54I

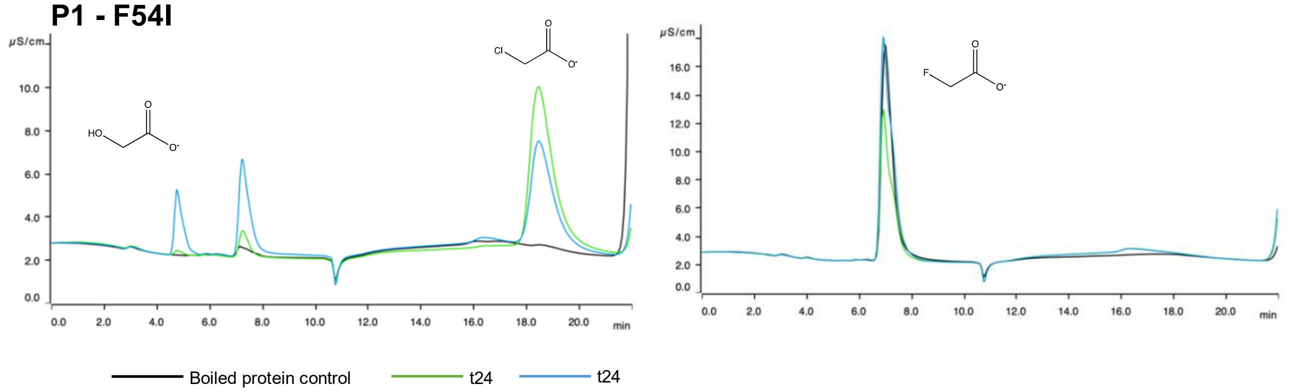

Protein variant	substrate	% conversion	concentration glycolate [mM]
P1-F54I	chloroacetate 4mM	25%	1.01
P1-F54I	fluoroacetate 4mM	0%	<LOD

P1 - T57M

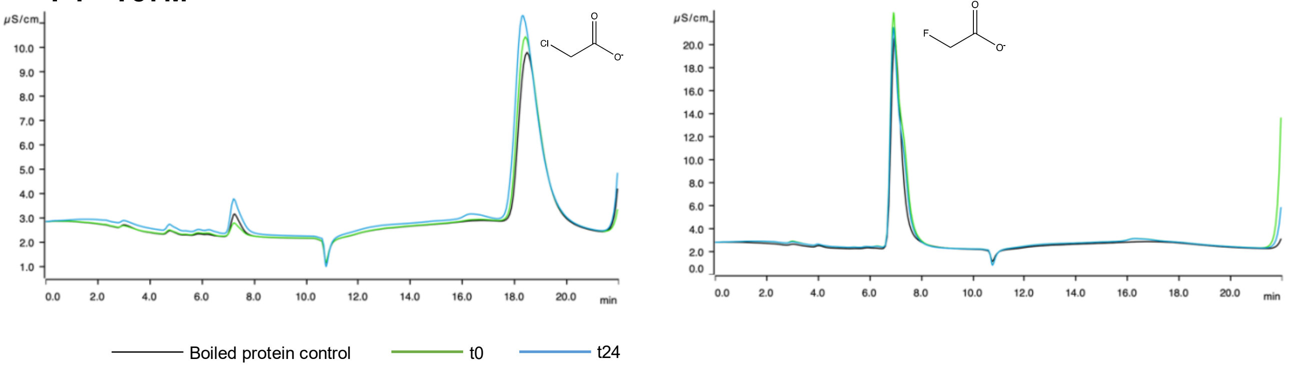

Protein variant	substrate	% conversion	concentration glycolate [mM]
P1-T57M	chloroacetate 4mM	0%	<LOD
P1-T57M	fluoroacetate 4mM	0%	<LOD

P1 - H74F

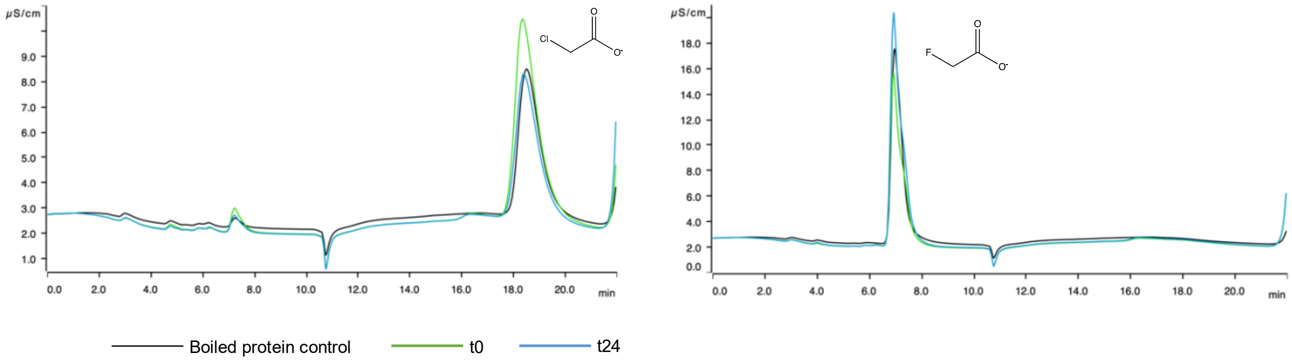

Protein variant	substrate	% conversion	concentration glycolate [mM]
P1-H74F	chloroacetate 4mM	0%	<LOD
P1-H74F	fluoroacetate 4mM	0%	<LOD

Figure S22: Ion chromatography traces of the single amino acid variants for protein P1 (*Enterocloster aldenensis*) with two different substrates. Left column: chloroacetate; Right column: fluoroacetate at different timepoints. Black: boiled protein control; Green: t0h; Blue: t24h.

The tables show the conversion and measured glycolate concentration. Glycolate is used for this calculation due to possible interfering chloride contaminations at min 7.0.

Substrate: chloroacetate fluoroacetate

P1 - F54I + T57M

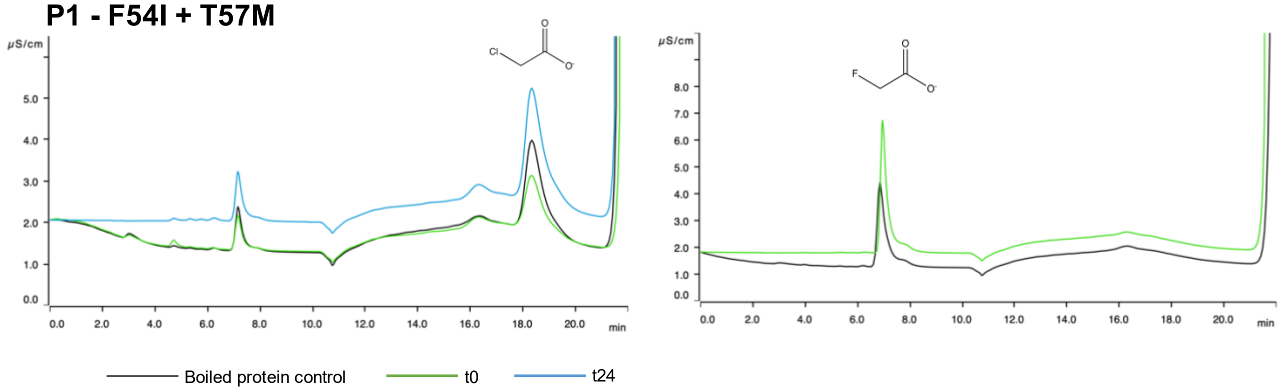

Protein variant	substrate	% conversion	concentration glycolate [mM]
P1-F54I+T57M	chloroacetate 1 mM	0%	<LOD
P1-F54I+T57M	fluoroacetate 1 mM	0%	<LOD

P1 - F54I+ H74F

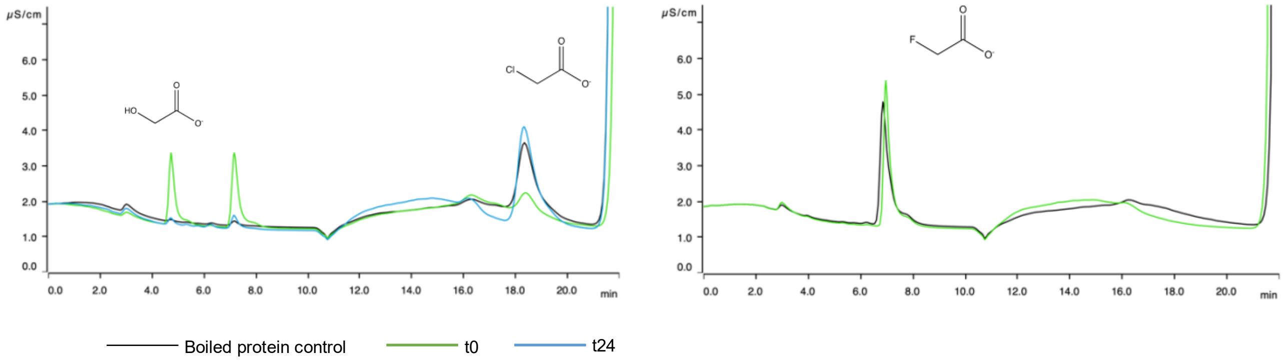

Protein variant	substrate	% conversion	concentration glycolate [mM]
P1-F54I+T57M	chloroacetate 1 mM	23%	0.326
P1-F54I+T57M	fluoroacetate 1 mM	0%	<LOD

P1 - F54I + F187S

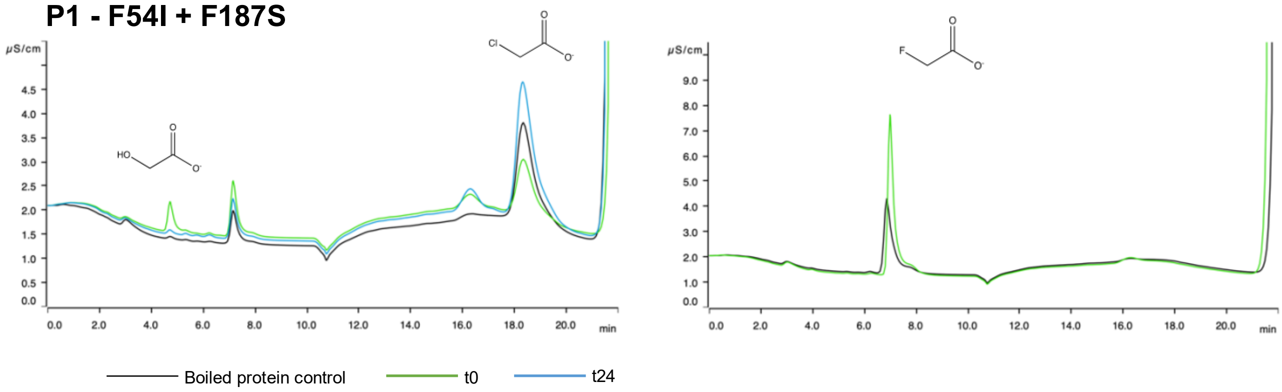

Protein variant	substrate	% conversion	concentration glycolate [mM]
P1-F54I+T57M	chloroacetate 1 mM	1.6%	0.07
P1-F54I+T57M	fluoroacetate 1 mM	0%	<LOD

Figure S23: Ion chromatography traces of the double mutants for protein P1 (*Enterocloster aldenensis*) with two different substrates. Left column: chloroacetate; Black: boiled protein control; Blue: t0h; Green: t24h. The tables show the conversion and measured glycolate concentration. Glycolate is used for this calculation due to possible interfering chloride contaminations at min 7.0.

Substrate: chloroacetate fluoroacetate

P1 - F54I + T57M + H74F

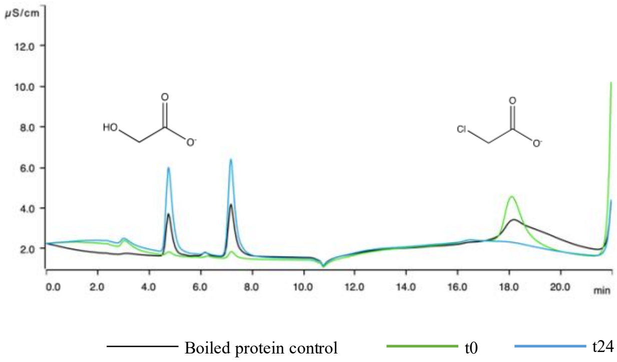

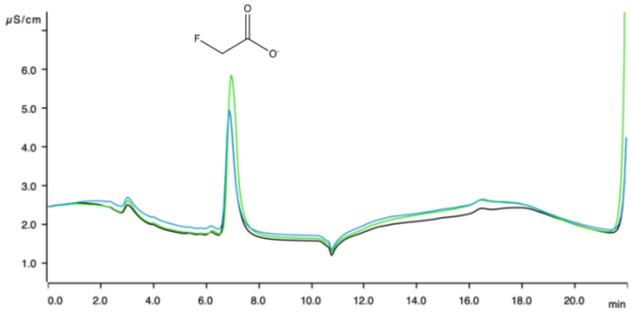

Protein variant	substrate	% conversion	concentration glycolate [mM]
P1-F54I+T57M+H74F	chloroacetate 1 mM	118%	1.184
P1-F54I+T57M+H74F	fluoroacetate 1 mM	0%	<LOD

P1 - F54I + H74F + F187S

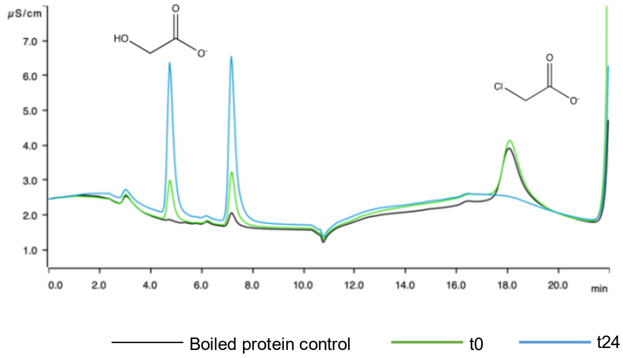

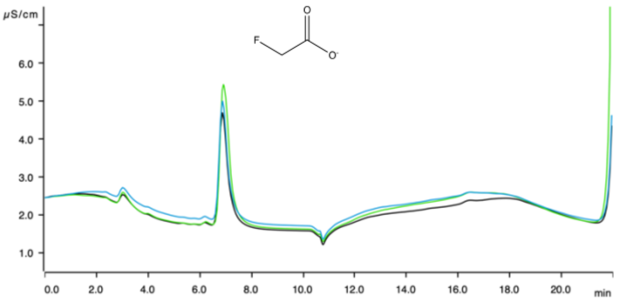

Protein variant	substrate	% conversion	concentration glycolate [mM]
P1-F54I+H74F+F187S	chloroacetate 1 mM	112%	1.128
P1-F54I+H74F+F187S	fluoroacetate 1 mM	0%	<LOD

P1 - F54I + F187S + T57M

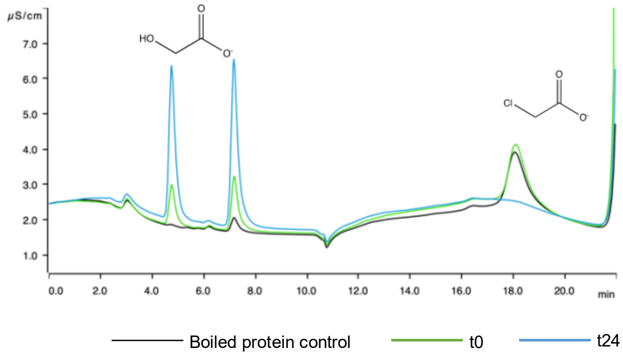

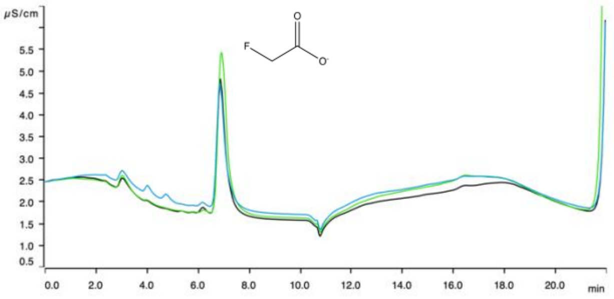

Protein variant	substrate	% conversion	concentration glycolate [mM]
P1-F54I+T57M+F187S	chloroacetate 1 mM	117%	1.172
P1-F54I+T57M+F187S	fluoroacetate 1 mM	0%	<LOD

Figure S24: Ion chromatography traces of the triple mutants for protein WP_118709078.1 (*Enterocloster aldenensis*) with two different substrates. Left column: chloroacetate Black: boiled protein control; Green: t0h; Blue: t24h. The tables show the conversion and measured glycolate concentration. Glycolate is used for this calculation due to possible interfering chloride contaminations at min 7.0. Conversion values >100% are due to instrument variability and slight evaporation which could not be fully avoided.

Substrate: chloroacetate fluoroacetate

P1 - F54I + F187S + T57M + H74F

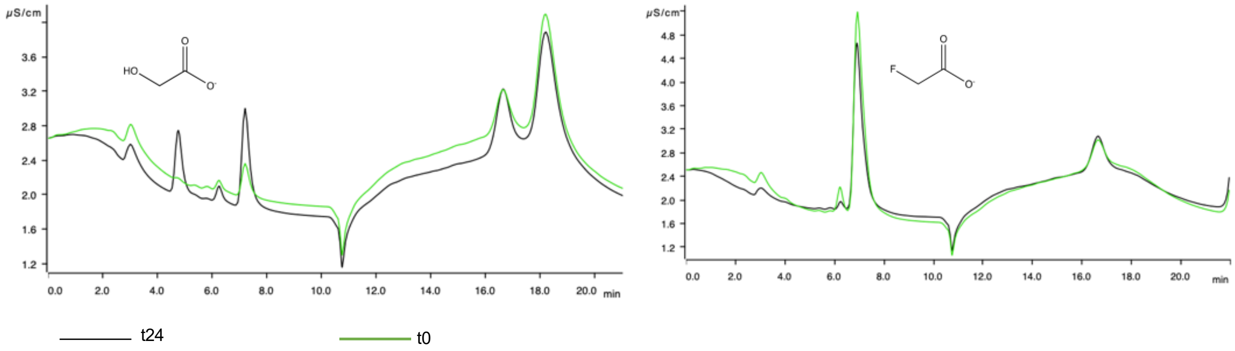

Protein variant	substrate	% conversion	concentration glycolate [mM]
P1-F54I+T57M+F187S	chloroacetate 1 mM	8.6%	0.086
P1-F54I+T57M+F187S	fluoroacetate 1 mM	0%	<LOD

Figure S25: Ion chromatography traces of the quadruple mutants for protein P1 (*Enterocloster aldenensis*) with two different substrates. Left column: chloroacetate Black: t24; Green: t0h. The table shows the conversion and measured glycolate concentration. Glycolate is used for this calculation due to possible interfering chloride contaminations at min 7.0.

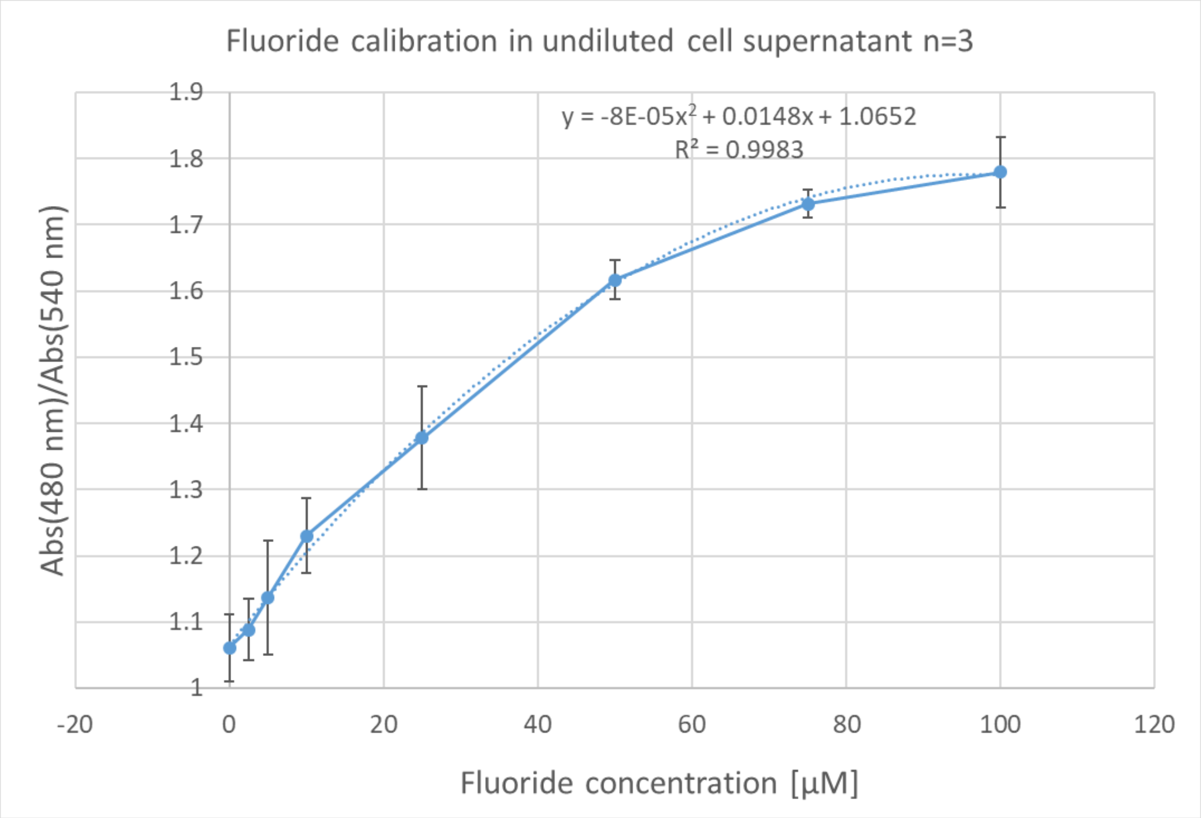

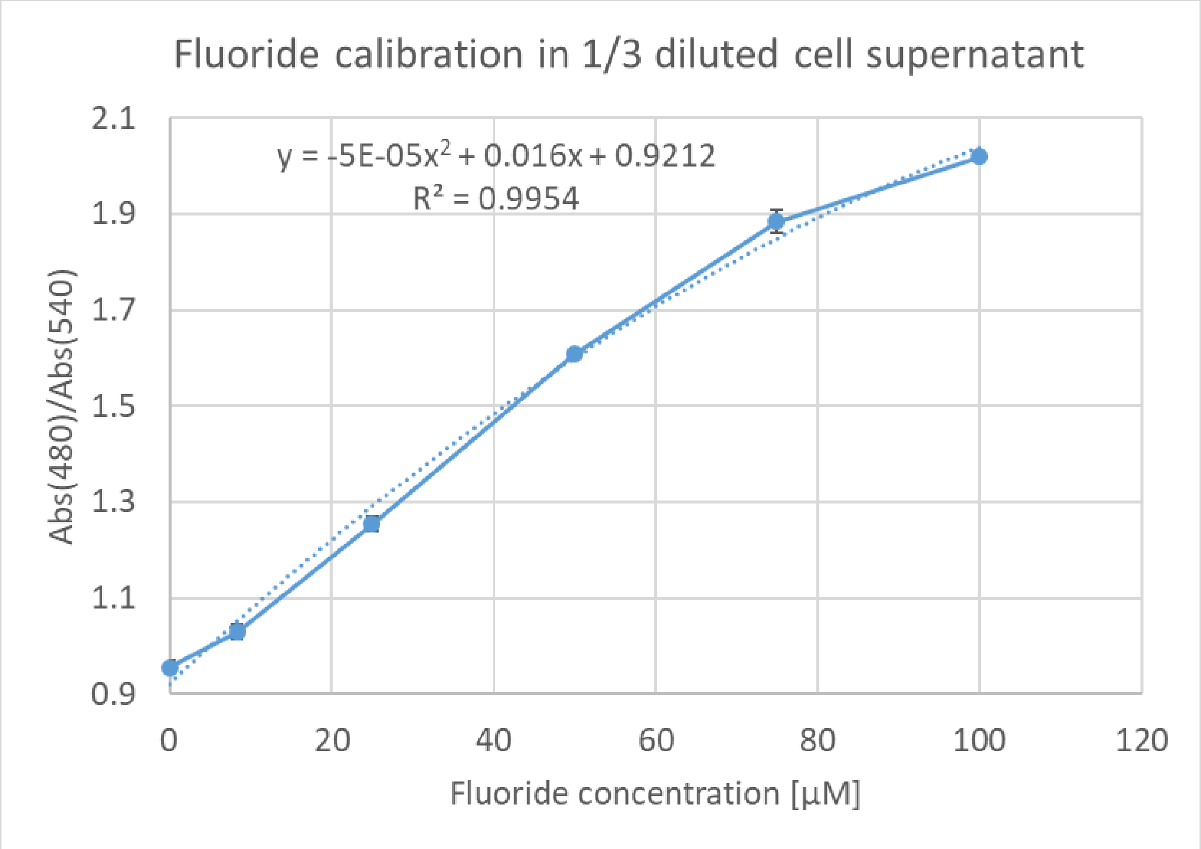

Figure S26: Miniaturized new xylenol orange colorimetric assay: Standards in undiluted and ⅓ diluted cell supernatant of *E. coli* producing inactive enzyme, which were used to calibrate the measurements of the alanine scanning library in the respective dilution.

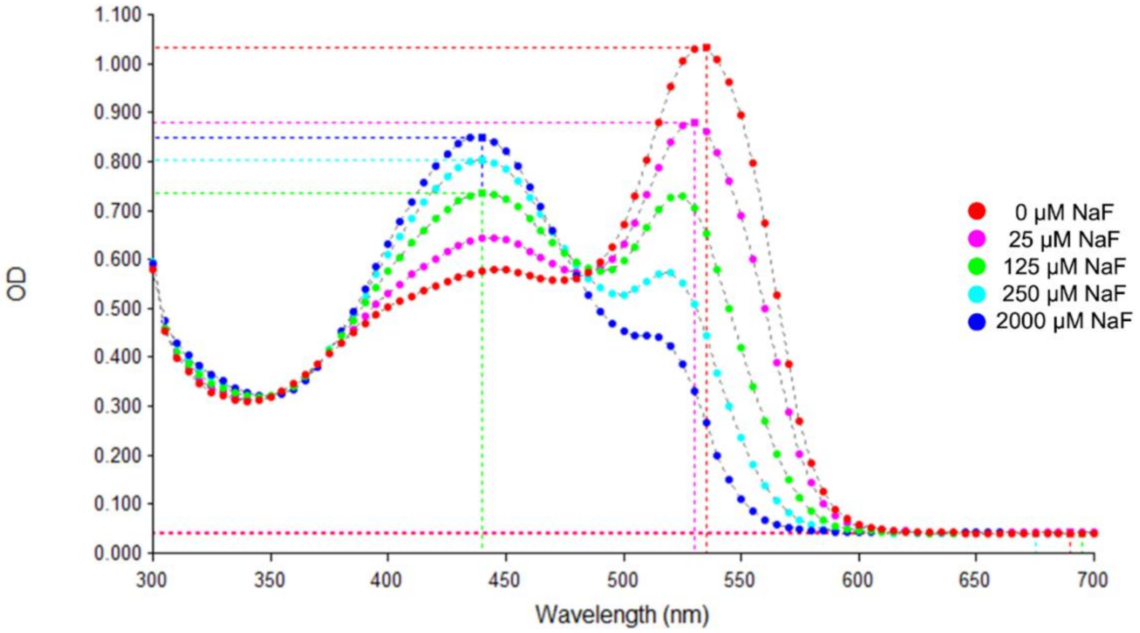

Figure S27: Isosbestic points measured using the new miniaturized xylenol orange assay. Absorbance spectra are shown for a standard curve of fluoride ion concentrations.

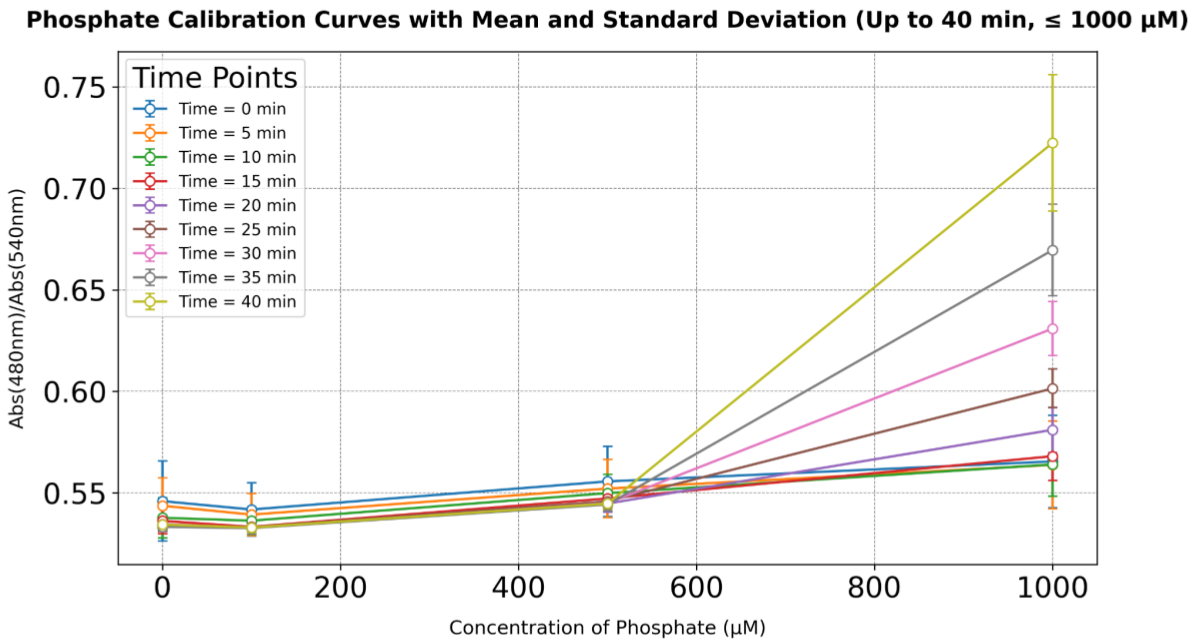

Figure S28: Interference of phosphate on the fluoride assay relative to concentration and time. 4 replicates of each phosphate concentration were measured during 40 min. No change in absorption was measured up to a concentration of phosphate of 0.5 mM. Phosphate was shown to disrupt the red complex similarly to fluoride, but at higher concentrations and more slowly.

P9 *Priestia megaterium*

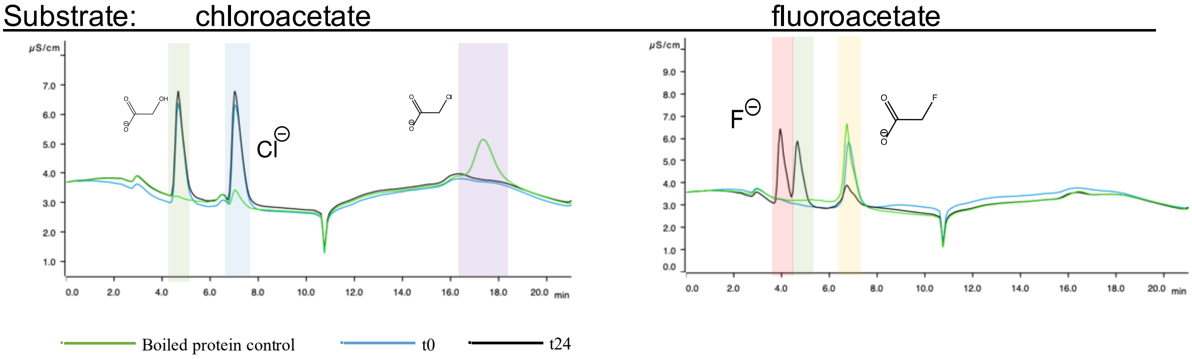

Protein	substrate	% conversion	concentration glycolate [mM]
P9	chloroacetate 1 mM	62%	0.62
P9	fluoroacetate 1 mM	50%	0.5

Figure S29: Ion chromatography traces of protein P9 (*Priestia megaterium)* with two different substrates. Green: control; Blue: t0h; Black: t24h. Chloroacetate shaded in purple, fluoroacetate shaded in yellow, glycolate shaded in green, chloride shaded in blue, and fluoride shaded in red. The table shows the conversion and measured glycolate concentration. Glycolate is used for this calculation due to possible interfering chloride contaminations.

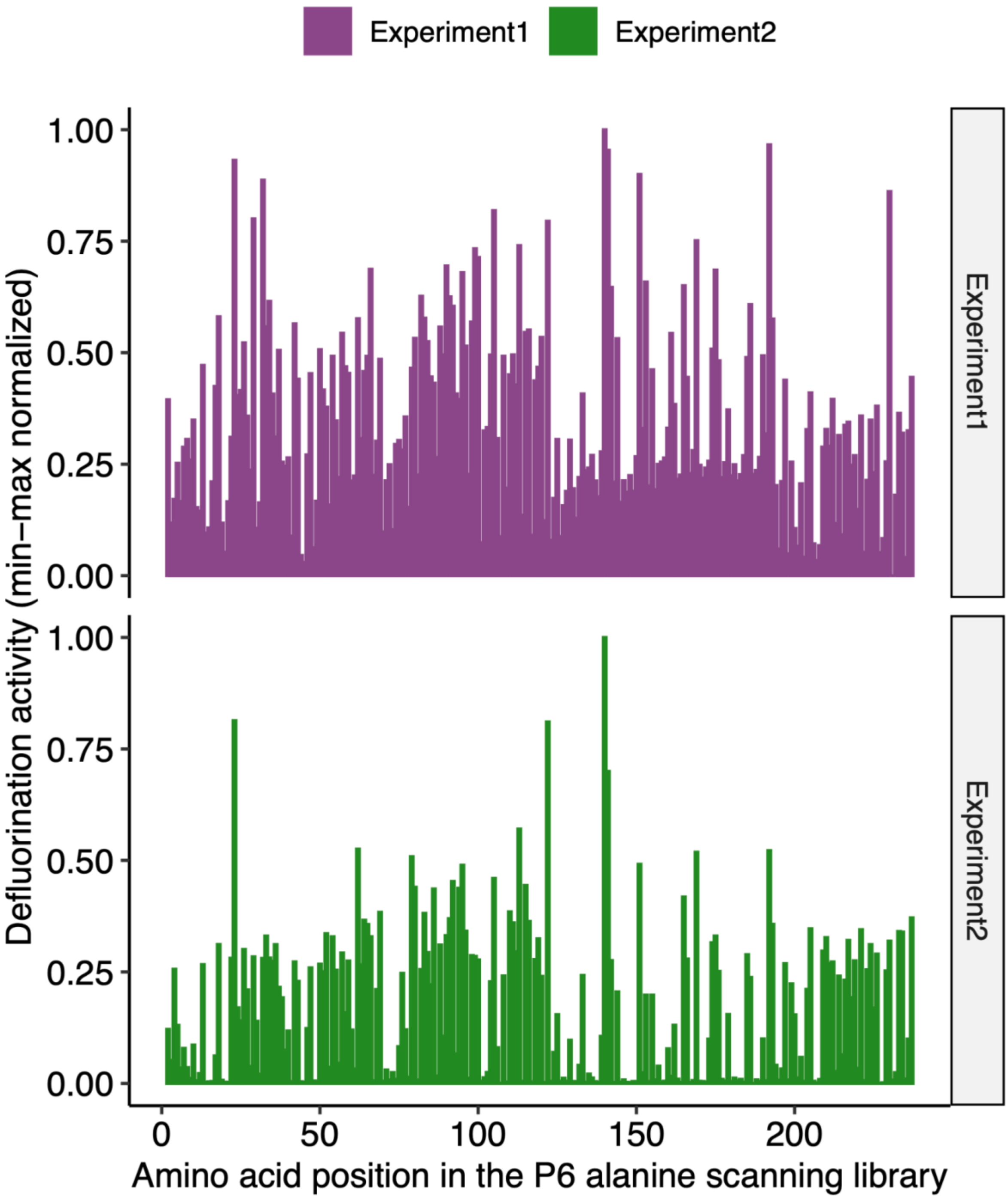

Figure S30: Comparison of Experiment 1 and Experiment 2 results from defluorination activity screening of the alanine scanning library. Overall background levels are higher in Experiment 1 due to the introduction of additional washing and dilution steps in Experiment 2 which increased the signal-to-noise ratio.

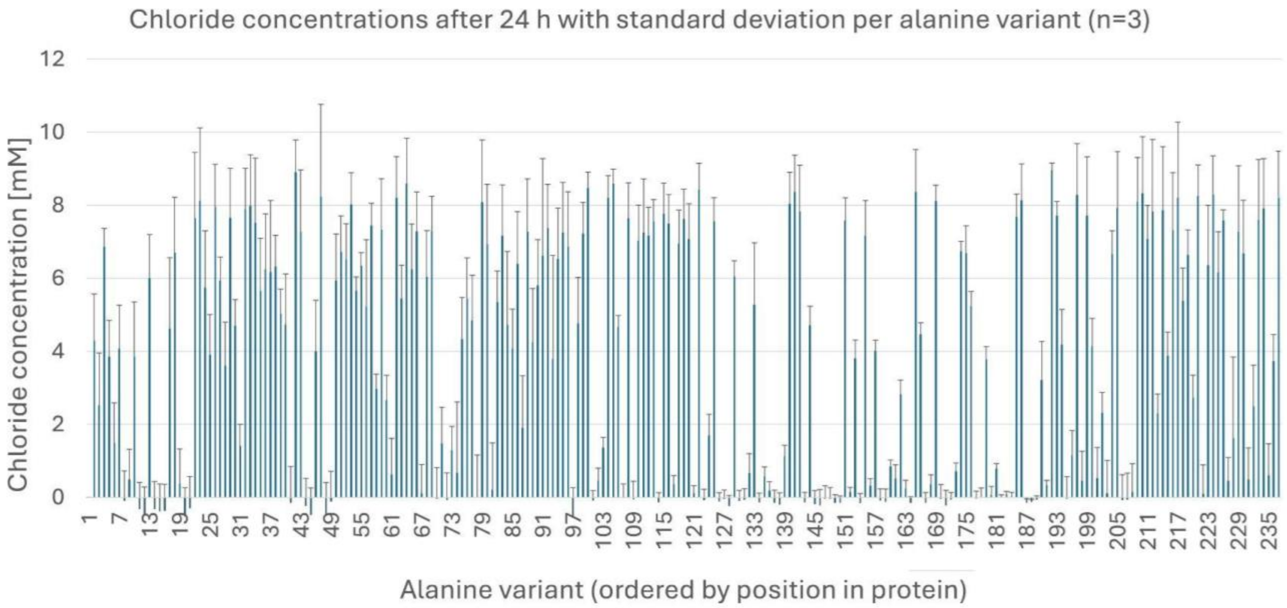

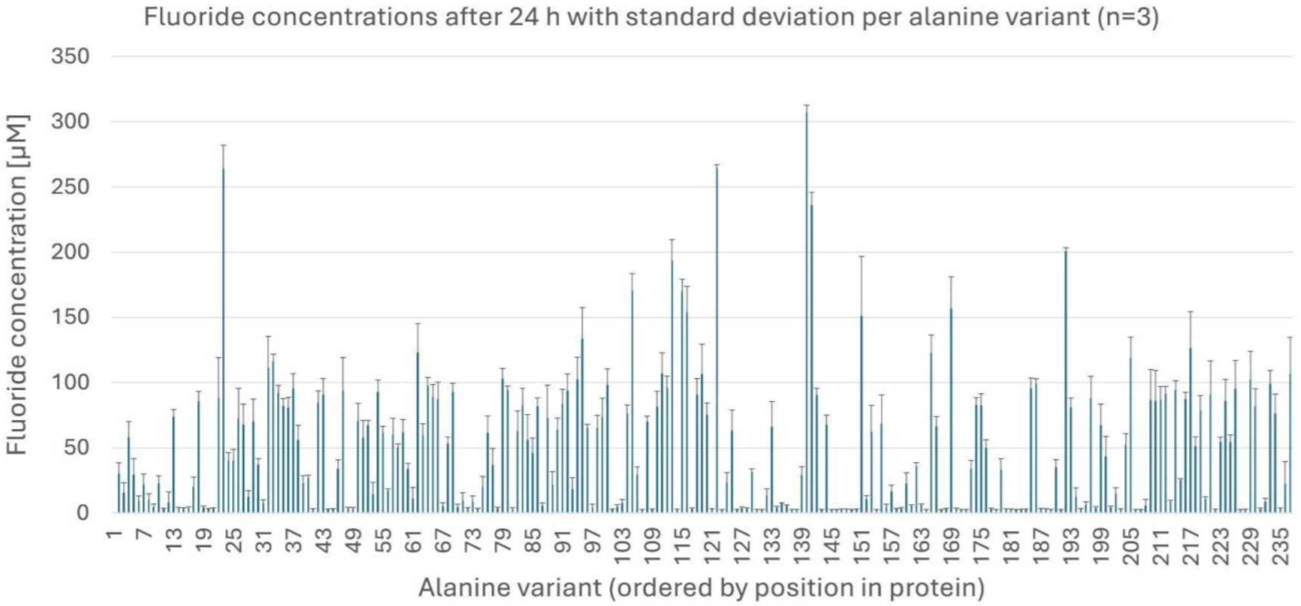

Figure S31: Mean fluoride and chloride concentrations and standard deviations of 3 independent replicates for each mutant. Fluoride and chloride concentrations were measured with colorimetric assays. The first measurement corresponds to the second amino acid mutated to alanine.

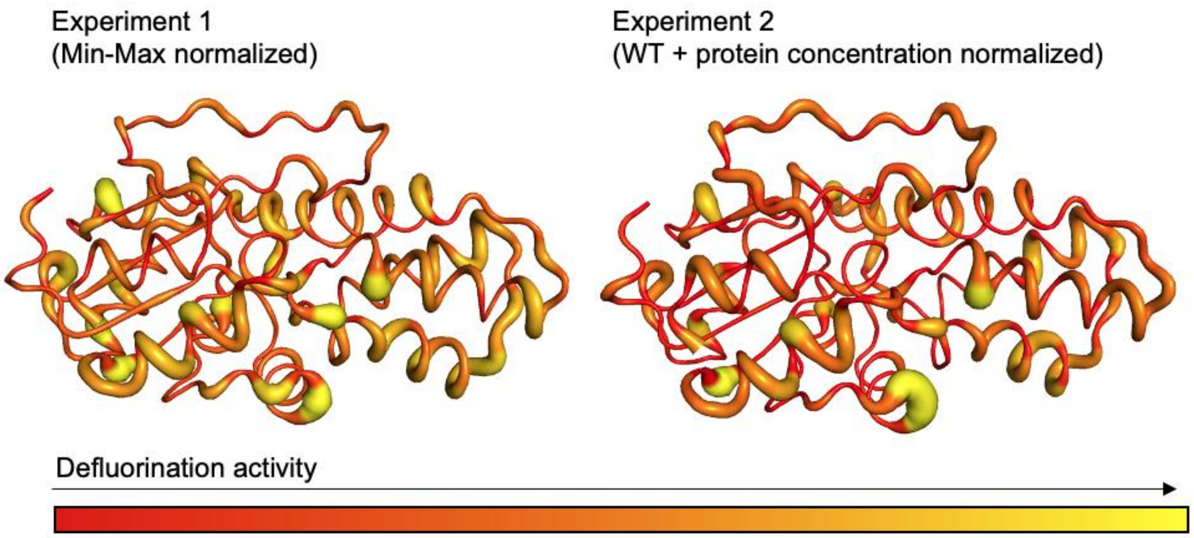

Figure S32: Defluorination activity of the alanine scanning for protein P6 for experiment 1 and 2. The defluorination activity is based on the measured fluoride concentration of our alanine scanning experiment. The representation shows the merged protein variants. The higher the measured fluoride concentration for the individual variant the thicker and brighter is the line in the representation at the location of the mutated amino acid.

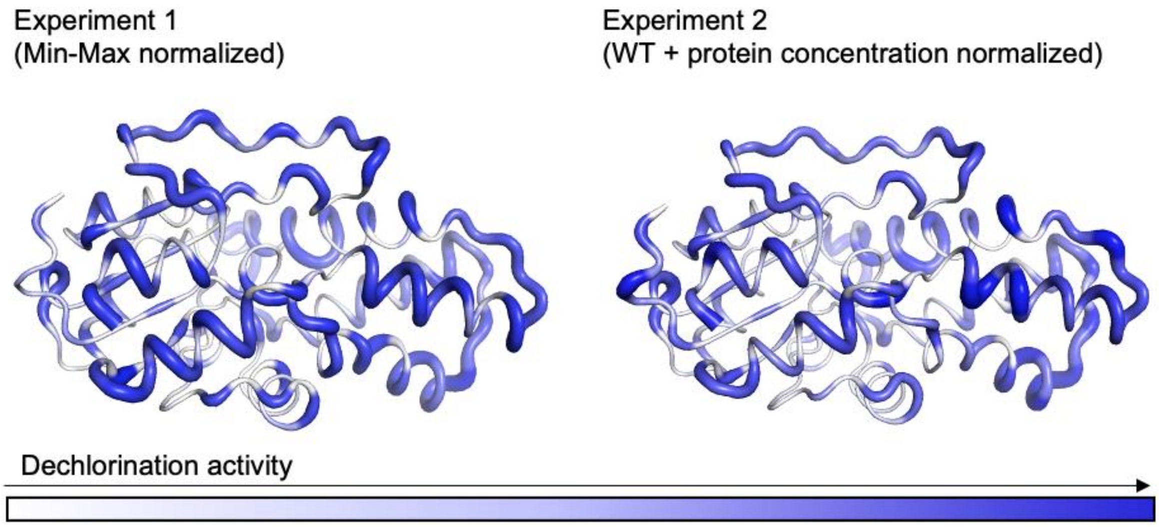

Figure S33: Dechlorination activity of the alanine scanning for protein P6 for experiment 1 and 2. The dechlorination activity is based on the measured chloride concentration of our alanine scanning experiment. The representation shows the merged protein variants. The higher the measured chloride concentration for the individual variant the thicker and bluer is the line in the representation at the location of the mutated amino acid.

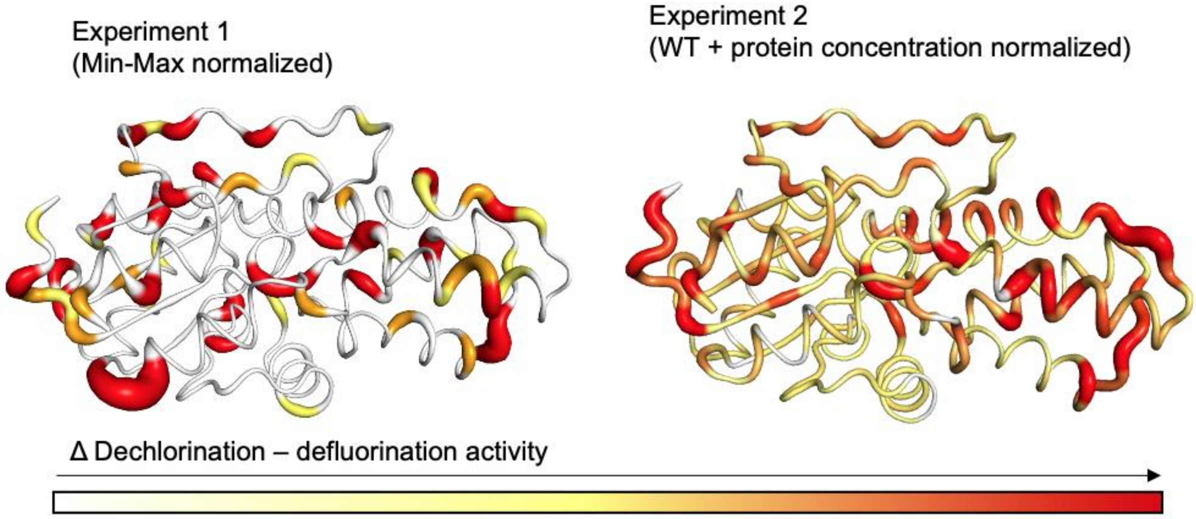

Figure S34: Defluorination influence. Defluorination activity subtracted from the dechlorination activity results of the alanine scanning for protein P6 from experiment 1 and 2. The defluorination activity and the dechlorination activity is based on the measured fluoride and chloride concentration of our alanine scanning experiments. The representation shows the merged protein variants. P6 alanine variants which lost their defluorination activity but kept their dechlorination activity influencing the defluorination. The thicker and redder the line in the representation, the greater the difference between measured chloride and fluoride concentrations for the P6 variant with the alanine mutation at that location.

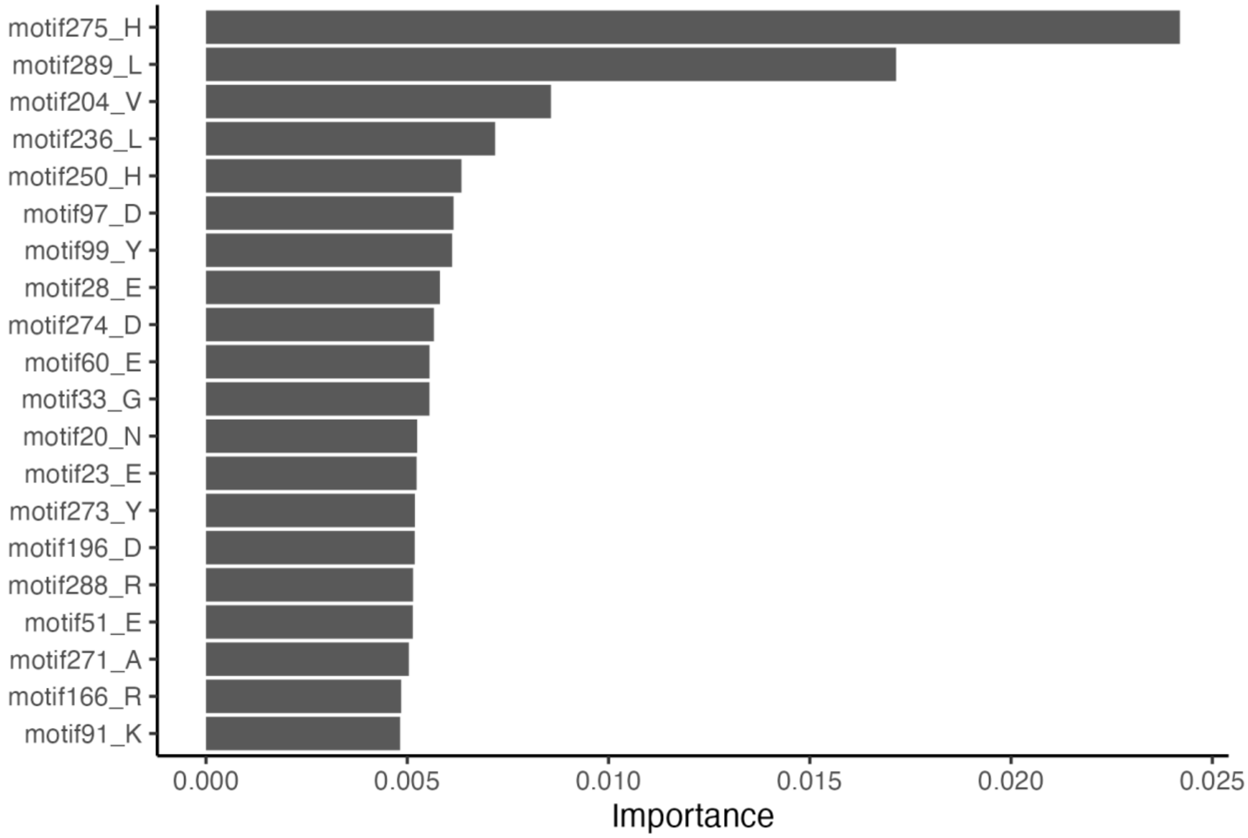

Figure S35: Experiment 1 trained random forest classification model identified the top twenty most important features to separate defluorinating from non-defluorinating proteins. Feature importance was determined by permutation.

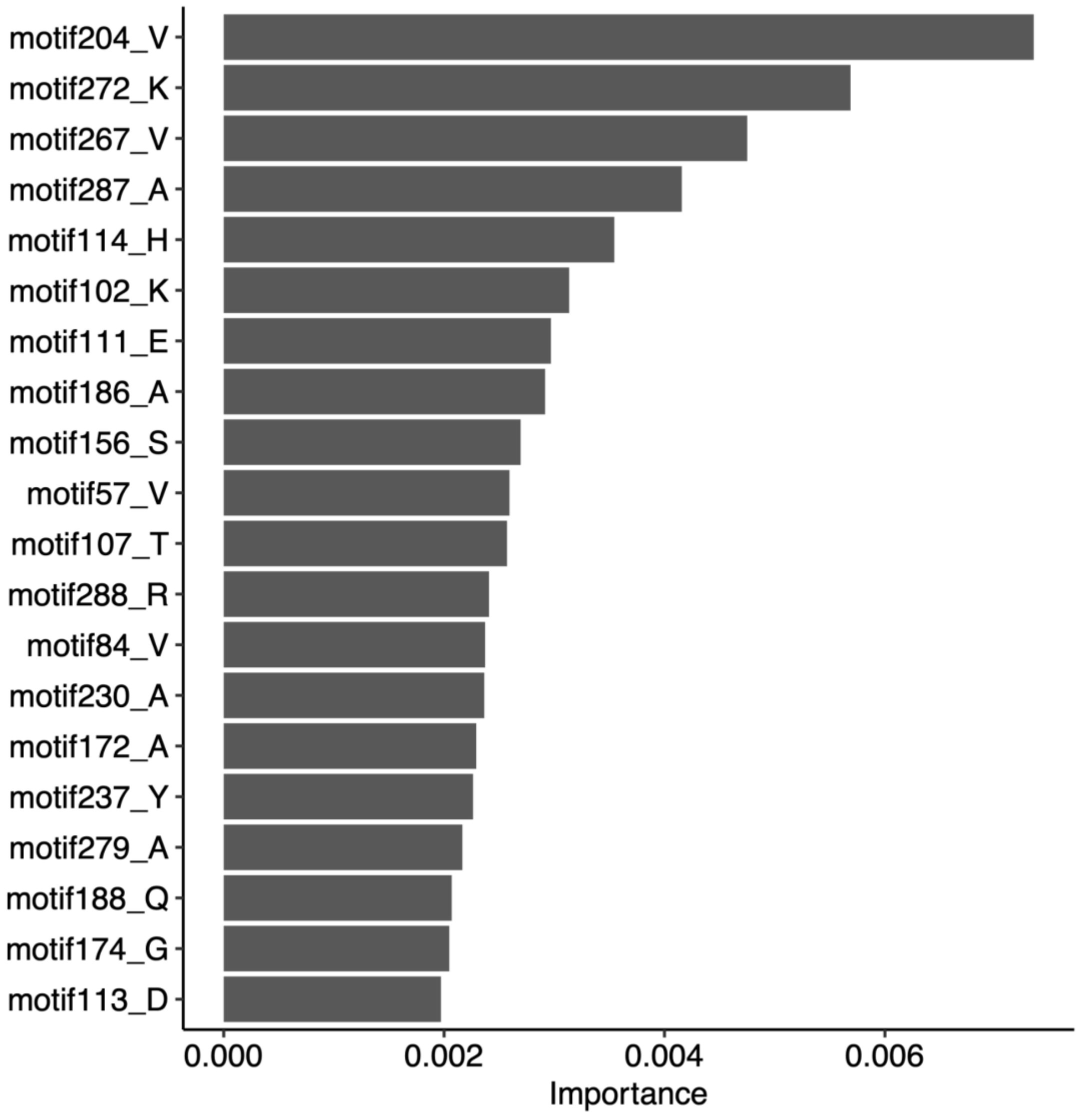

Figure S36: **Experiment 2 trained random forest classification model** identified the top twenty most important features to separate defluorinating from non-defluorinating proteins. Feature importance was determined by permutation.

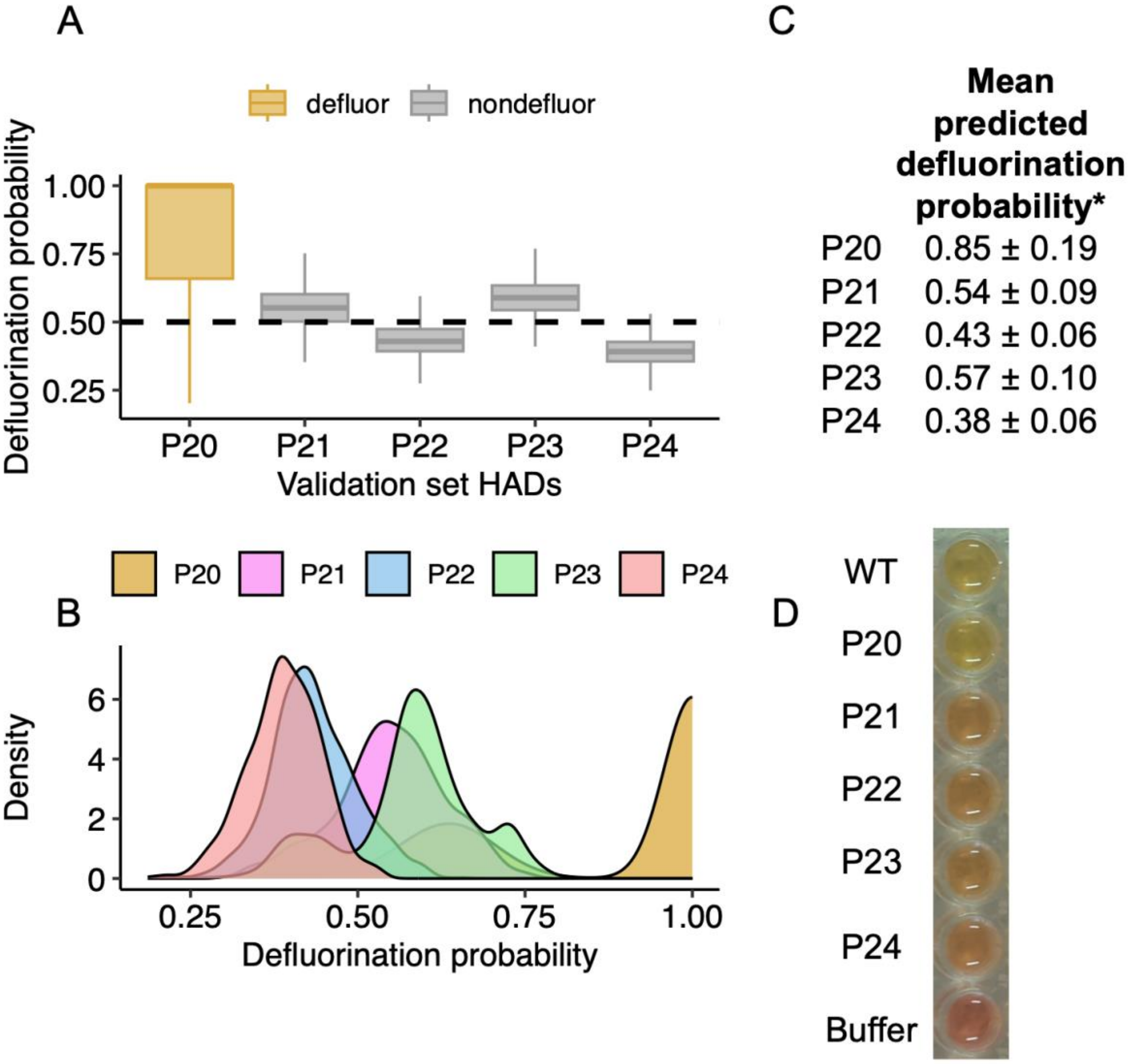

Figure S37. A) Comparison of defluorination probabilties predicted for five new validation set HADs. P20 = *Raoultibacter phocaeensis (*WP_139652913.1); P21 = *Stellaceae bacterium* (HTS94301.1); P22 = *Ca. Rokuibacteriota (*PYN88313.1)*;* P23 = *Gemmatimonadota bacterium (*MDH3205452.1); P24 = *Eubacterium sp. (*MBS6900484.1). B) Density plots of the probabilities demonstrate the higher probability distribution of P20 relative to other validation set HADs. C) Mean predicted defluorination probability scores for the validation set sequences predicted by random forest classification. D) Results from the colorimetric fluoride ion detection assay demonstrating defluorination activity of P20 comparable to wild-type (WT) while P21-P24 have no activity (slight assay discoloration is due to protein, comparable to a buffer control).

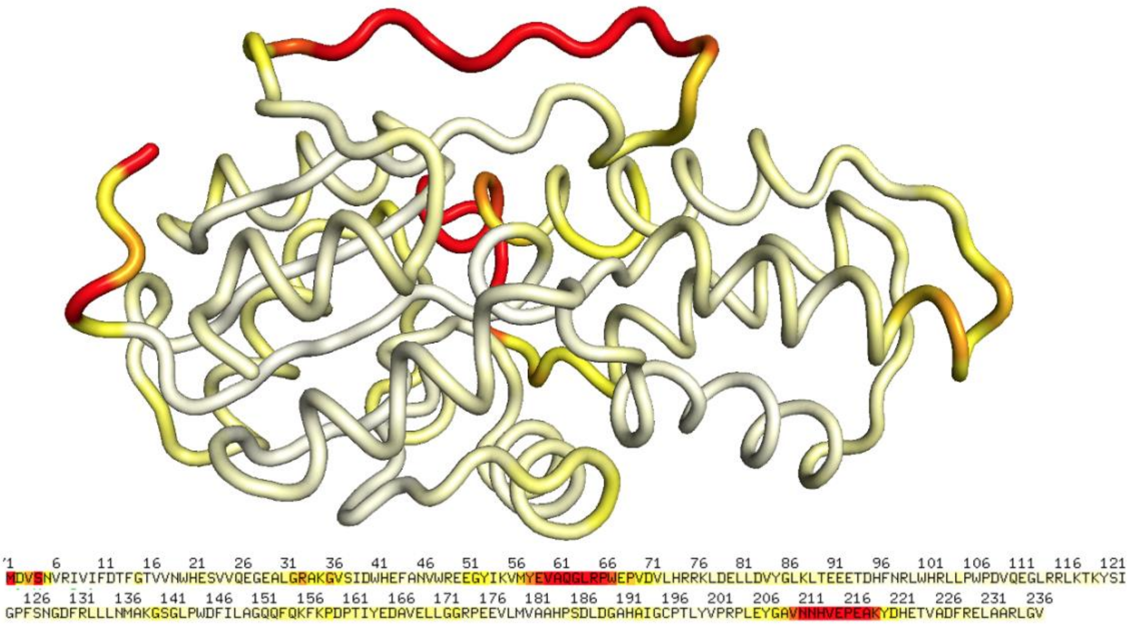

Figure S38: Visualization of the Root Mean Square Fluctuation (RMSF) of the MD simulation of P6. The RMSF represents the flexibility of the protein. The color represents the flexibility from small to high with the corresponding colors from white to yellow to red.

Chimera: Substrate: chloroacetate fluoroacetate

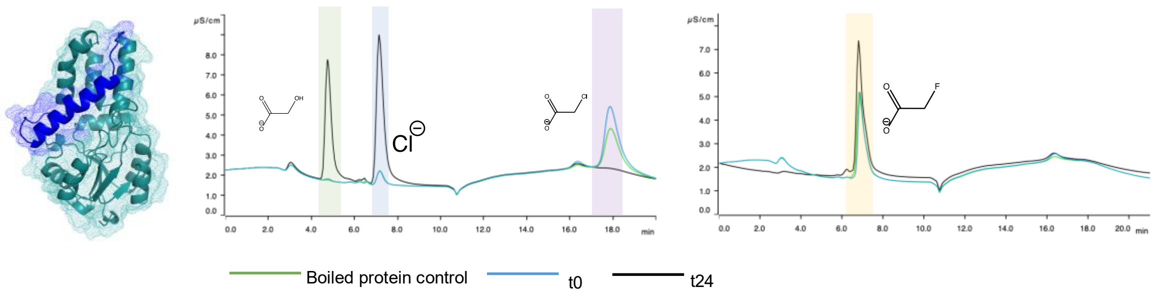

Protein	substrate	% conversion	concentration glycolate [mM]
P1-P6-blue	chloroacetate 1.3 mM	75	0.98
P1-P6-blue	fluoroacetate 1 mM	0	<LOD

Chimera: Substrate: chloroacetate fluoroacetate

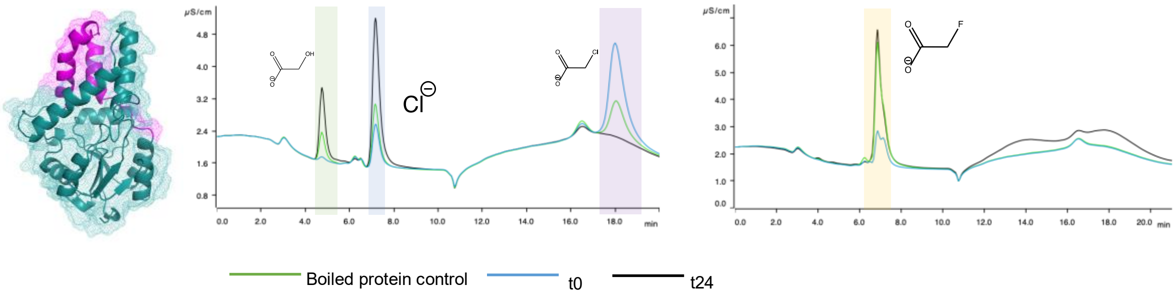

Protein	substrate	% conversion	concentration glycolate [mM]
P1-P6-pink	chloroacetate 0.6 mM	68.66	0.43
P1-P6-pink	fluoroacetate 1 mM	0	<LOD

Chimera: Substrate: chloroacetate fluoroacetate

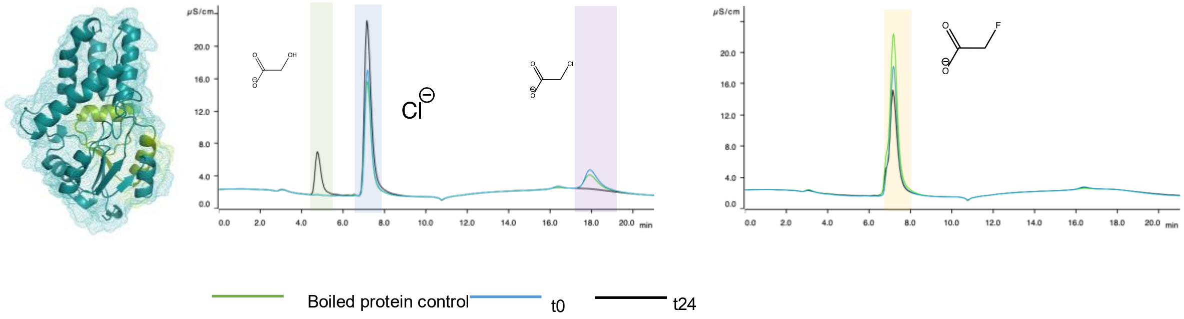

Protein	substrate	% conversion	concentration glycolate [mM]
P1-P6-green	chloroacetate 1.2 mM	72.5	0.87
P1-P6-green	fluoroacetate 1 mM	0	<LOD

Chimera: Substrate: chloroacetate fluoroacetate

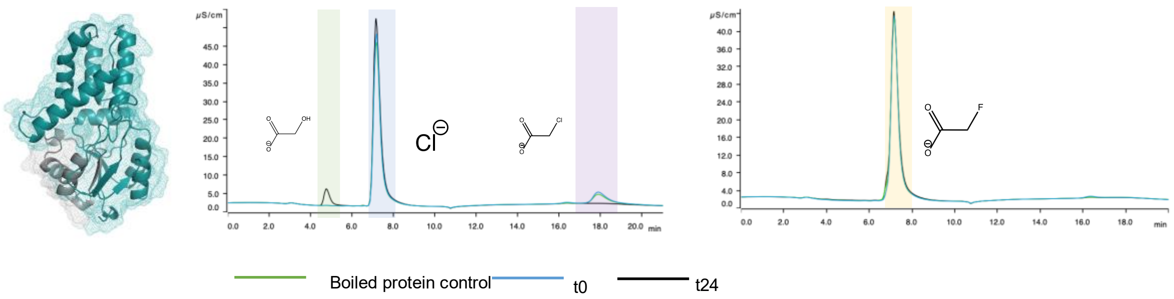

Protein	substrate	% conversion	concentration glycolate [mM]
P1-P6-green	chloroacetate 1.8 mM	75.7	1.4
P1-P6-green	fluoroacetate 1 mM	0	<LOD

Chimera: Substrate: chloroacetate fluoroacetate

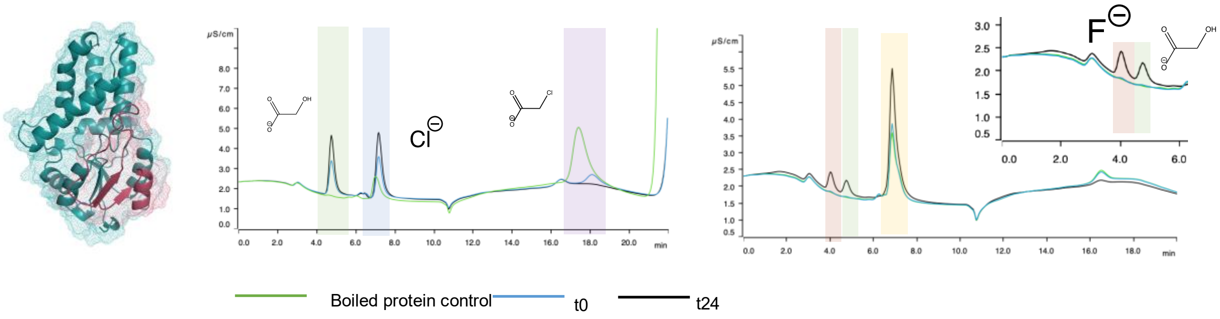

Protein	substrate	% conversion	concentration glycolate [mM]
P1-P6-red	chloroacetate 1 mM	84	0.84
P1-P6-red	fluoroacetate 1.14 mM	2.2	0.022

Figure S39: Ion chromatograms of protein in vitro assays with the different chimeric proteins. Tested substrates are fluoroacetate and chloroacetate. Blue: 0h; green: boiled protein control; black: 24h. The turquoise part of the protein structure corresponds to the non-deflourinating protein P1. The colored part of the protein structure corresponds to the homologous region in the defluorinating protein P6.
Chloroacetate shaded in purple, fluoroacetate shaded in yellow, glycolate shaded in green, chloride shaded in blue, and fluoride shaded in red. The tables show the conversion and measured glycolate concentration. Glycolate is used for this calculation due to possible interfering chloride contaminations.

Supplementary tables:

Table S1: Proteins from the human gut microbiota database (HumGut DB with similarity to the query sequence P5 (PDB ID: 3UMG).

Query	Subject	% Identity	Alignment length	e-value	Bit score
3UMG_1|Chains	WP_121129595.1	46.186	236	5.50e-74	217
3UMG_1|Chains	KUG38984.1	43.162	234	2.11e-63	191
3UMG_1|Chains	NWK50657.1	42.308	234	1.01e-61	186
3UMG_1|Chains	WP_227153894.1	41.818	55	1.59e-09	47.8
3UMG_1|Chains	WP_157004912.1	41.818	55	1.61e-09	47.8
3UMG_1|Chains	WP_087191411.1	41.818	55	1.69e-09	47.8
3UMG_1|Chains	WP_004693440.1	41.453	234	1.76e-60	183
3UMG_1|Chains	WP_151836607.1	40.773	233	3.70e-61	184
3UMG_1|Chains	WP_178618037.1	40	235	5.51e-56	171
3UMG_1|Chains	WP_118709330.1	39.344	61	1.18e-06	39.3
3UMG_1|Chains	EEQ56360.1	39.224	232	4.13e-55	169
3UMG_1|Chains	WP_118709078.1	38.462	234	1.12e-53	166
3UMG_1|Chains	WP_006686258.1	33.6	125	4.87e-09	46.2
3UMG_1|Chains	HCH98167.1	33.333	87	2.96e-09	47
3UMG_1|Chains	PWL57511.1	33.333	87	2.98e-09	47
3UMG_1|Chains	RGD90338.1	32.645	242	1.25e-42	137
3UMG_1|Chains	WP_008979552.1	30.682	88	1.67e-09	47.8
3UMG_1|Chains	WP_008689090.1	30.682	88	1.78e-09	47.4
3UMG_1|Chains	WP_132223766.1	30.682	88	1.81e-09	47.4
3UMG_1|Chains	WP_227150888.1	30.682	88	1.90e-09	47.4
3UMG_1|Chains	MBS5585750.1	30.57	193	7.32e-10	48.9
3UMG_1|Chains	HIV28856.1	30.57	193	7.53e-10	48.9
3UMG_1|Chains	ERI71420.1	30.208	96	1.37e-09	48.1
3UMG_1|Chains	WP_117543986.1	30.208	96	1.44e-09	48.1
3UMG_1|Chains	GKH53471.1	29.487	78	4.91e-08	43.5
3UMG_1|Chains	WP_156074201.1	29	200	6.46e-11	51.6
3UMG_1|Chains	WP_270479031.1	29	200	1.04e-10	51.2
3UMG_1|Chains	WP_249259624.1	27.542	236	1.53e-11	53.5
3UMG_1|Chains	WP_036120264.1	27.273	110	1.45e-07	42
3UMG_1|Chains	WP_027291866.1	27.193	228	3.13e-11	52.8
3UMG_1|Chains	WP_112144081.1	27.136	199	2.74e-10	50.1
3UMG_1|Chains	WP_053428405.1	26.432	227	6.59e-17	68.9
3UMG_1|Chains	WP_027290230.1	25.833	240	2.97e-08	43.9
3UMG_1|Chains	WP_026089469.1	25.652	230	3.75e-12	55.5
3UMG_1|Chains	WP_081745164.1	25.243	206	3.40e-14	61.2
3UMG_1|Chains	WP_069514564.1	25	208	3.81e-09	46.6
3UMG_1|Chains	WP_102290114.1	24.757	206	1.06e-13	59.7
3UMG_1|Chains	WP_066861885.1	24.309	181	4.71e-09	46.2
3UMG_1|Chains	HIW22760.1	24.257	202	5.08e-13	57.8

3UMG_1|Chains	WP_263041397.1	24.257	202	7.05e-13	57.4
3UMG_1|Chains	WP_031546286.1	24	200	5.11e-09	46.2
3UMG_1|Chains	WP_005951934.1	23.81	210	1.66e-08	44.7
3UMG_1|Chains	CDD30753.1	23.669	169	7.97e-10	48.9
3UMG_1|Chains	WP_048310185.1	23.478	230	2.06e-09	47.4
3UMG_1|Chains	WP_098579072.1	23.394	218	3.01e-10	49.7
3UMG_1|Chains	WP_050688410.1	23.394	218	5.37e-10	48.9
3UMG_1|Chains	WP_064471359.1	23.394	218	5.58e-10	48.9
3UMG_1|Chains	WP_107514404.1	22.984	248	2.37e-13	58.9
3UMG_1|Chains	CDD29996.1	22.772	202	3.46e-12	55.5
3UMG_1|Chains	WP_065338764.1	22.5	240	6.96e-14	60.5
3UMG_1|Chains	WP_034355080.1	22.326	215	5.19e-09	46.2
3UMG_1|Chains	WP_034377360.1	22.326	215	5.39e-09	46.2
3UMG_1|Chains	WP_053359908.1	22.273	220	1.38e-10	50.8
3UMG_1|Chains	WP_036125194.1	22.222	198	9.13e-10	48.5
3UMG_1|Chains	WP_187031722.1	22.176	239	5.65e-12	55.1
3UMG_1|Chains	WP_070051353.1	21.951	205	2.67e-13	58.5
3UMG_1|Chains	WP_029378231.1	21.635	208	4.55e-13	58.2
3UMG_1|Chains	WP_233640236.1	21.635	208	5.07e-13	57.8
3UMG_1|Chains	WP_009253081.1	21.296	216	2.99e-09	47
3UMG_1|Chains	WP_107597559.1	19.917	241	4.94e-10	49.3
3UMG_1|Chains	GKG91607.1	17.961	206	3.14e-04	32
3UMG_1|Chains	WP_087189991.1	17.961	206	3.27e-04	32

Table S2: Proteins selected for in vitro work. The previously characterized L-2 haloacid dehalogenase P5 (PDB ID: 3UMG) was included as a positive control.

NCBI identifier		NCBI taxonomy	HumGut DB taxonomy
WP_118709078.1	P1	*Enterocloster aldenensis*	*Faecalibacterium prausnitzii*
GKG91607.1	P2	MULTISPECIES: Gordonibacter	*Gordonibacter pamelaeae*
WP_087189991.1	P3	*Gordonibacter urolithinfaciens*	*Gordonibacter pamelaeae*
WP_004693440.1	P4	*Acinetobacter johnsonii*	*Acinetobacter johnsonii*
WP_011593529.1	P5	*Rhodococcus jostii* RHA1	Not in HumGut DB (soil bacterium)
WP_178618037.1	P6	*Guopingia tenuis*	*Catabacter* sp. UBA5399
WP_006686258.1	P7	*Citrobacter youngae*	*Citrobacter youngae*
WP_053428405.1	P8	*Rossellomorea marisflavi*	*Rossellomorea marisflavi*

Table S3: Genome neighborhood context of 55 bioinformatically-identified HADs in this study. The second most abundant PFAM is PF13419, referrs to the PFAM of the HAD queries. *Number of hits summed within 55 HAD genome neighborhoods **Frequency of the PFAM (per neighborhood) within ± 6 genes of the query HAD

ID	PFAM	Number	and descriptions of hits (for 55 HAD neighbourhoods)	Frequency*
1	-	169	NA/hypothetical proteins	1.352
2	PF13419	49	Haloacid dehalogenase-like hydrolase	0.392
3	PF00005	14	ABC transporter	0.112
4	PF12833	11	Helix-turn-helix domain	0.088
5	PF01380	9	SIS domain	0.072
6	PF00528	8	Binding-protein-dependent transport system inner membrane component	0.064
7	PF01019	8	Gamma-glutamyltranspeptidase	0.064
8	PF01261	8	Xylose isomerase-like TIM barrel	0.064
9	PF01381	8	Helix-turn-helix	0.064
10	PF00474	7	Sodium:solute symporter family	0.056
11	PF00480	6	ROK family	0.048
12	PF00583	6	Acetyltransferase (GNAT) family	0.048
13	PF00701	6	Dihydrodipicolinate synthetase family	0.048
14	PF03180	6	NlpA lipoprotein	0.048
15	PF04203	6	Sortase domain	0.048
16	PF00248	5	Aldo/keto reductase family	0.04
17	PF04131	5	Putative N-acetylmannosamine-6-phosphate epimerase	0.04
18	PF04464	5	CDP-Glycerol:Poly(glycerophosphate) glycerophosphotransferase	0.04
19	PF09587	5	Bacterial capsule synthesis protein PGA_cap	0.04
20	PF00072	4	Response regulator receiver domain	0.032
21	PF00117	4	Glutamine amidotransferase class-I	0.032
22	PF00535	4	Glycosyl transferase family 2	0.032
23	PF00664	4	ABC transporter transmembrane region	0.032
24	PF00860	4	Permease family	0.032
25	PF01022	4	Bacterial regulatory protein, arsR family	0.032

26	PF01451	4	Low molecular weight phosphotyrosine protein phosphatase	0.032
27	PF01743	4	Poly A polymerase head domain	0.032
28	PF02378	4	Phosphotransferase system, EIIC	0.032
29	PF02417	4	Chromate transporter	0.032
30	PF02436	4	Conserved carboxylase domain	0.032
31	PF02518	4	Histidine kinase-, DNA gyrase B-, and HSP90-like ATPase	0.032
32	PF02687	4	FtsX-like permease family	0.032
33	PF03358	4	NADPH-dependent FMN reductase	0.032
34	PF03466	4	LysR substrate binding domain	0.032
35	PF06962	4	Putative rRNA methylase	0.032
36	PF08028	4	Acyl-CoA dehydrogenase, C-terminal domain	0.032
37	PF08240	4	Alcohol dehydrogenase GroES-like domain	0.032
38	PF08282	4	haloacid dehalogenase-like hydrolase	0.032
39	PF08840	4	BAAT / Acyl-CoA thioester hydrolase C terminal	0.032
40	PF09359	4	VTC domain	0.032
41	PF13192	4	Thioredoxin domain	0.032
42	PF13302	4	Acetyltransferase (GNAT) domain	0.032
43	PF13338	4	Transcriptional regulator, AbiEi antitoxin	0.032
44	PF14262	4	Carbohydrate-binding domain-containing protein Cthe_2159	0.032
45	PF16316	4	Domain of unknown function (DUF4956)	0.032

Table S5: Chemicals used in this study

Chemical	Molecular structure	Abbr.	Supplier	CAS	Purity
chloroacetic acid	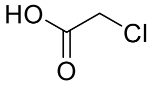	ClAc	Sigma Aldrich	3926-62-3	98 %
fluoroacetic acid	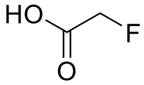	FAc	Sigma Aldrich, abcr	144-49-0	98 %
2,2-difluoroacetic acid	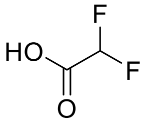	DFA	Sigma Aldrich	381-73-7	98 %
trifluoroacetic acid	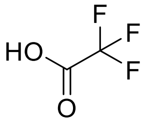	TFA	Sigma Aldrich	2923-18-4	98 %
2-fluoropropionic acid	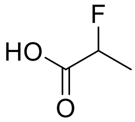	FP	Sigma Aldrich	6087-13-4	97 %
3-amino-2-fluoropropionic acid	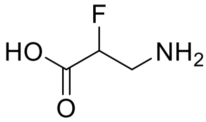	FBAL	Sigma Aldrich	3821-81-6	95 %
2-2-difluoropropionic acid	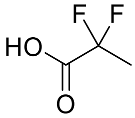	DFA	Sigma Aldrich	373-96-6	97 %
3,3,3-trifluoropropionic acid	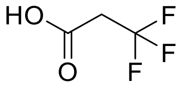	TFP	Sigma Aldrich	2516-99-6	98 %
5,5,5-trifluoropentanoic acid	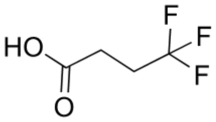	TFB	Sigma Aldrich	406-93-9	97%
heptafluorobutyric acid	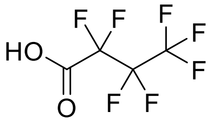	HFB	Sigma Aldrich	375-22-4	98%

Table S4: Defluorination data from *Gordonibacter pamelaeae* DSM 110924 in GMM medium. Incubated with 10 mM fluoroacetate for 24h. Std: standard, Gran linearization with slope index value -59.16. Measured with a Metrohm fluoride ion selective electrode (ISE).

Sample name	Standard added [mL]	[mV]	Gran linearization	Statistics	F^- conc [mg
G. pamelaeae in GMM-1	0	218.5	0.008103751	a: 0.121	0.24
	0.2	187.1	0.027644974	b: 0.006	
	0.2	170.4	0.053217788	r2: 0.998	
	0.2	160.6	0.078316624		
	0.2	153.6	0.10335019		
G. pamelaeae in GMM-2	0	217.1	0.008557575	a: 0.118	0.28
	0.2	186.3	0.028519298	b: 0.007	
	0.2	170.8	0.052395682	r2: 0.998	
	0.2	160.8	0.077709353		
	0.2	153.9	0.10215045		
G. pamelaeae in GMM-3	0	220.8	0.007409834	a: 0.124	0.18
	0.2	187.2	0.027537585	b: 0.004	
	0.4	161.2	0.076508902	r2: 0.999	
	0.6	143.6	0.15402154		
	0.8	131.3	0.253421984		
G. pamelaeae in GMM + 10mM FAc-1	0	171.4	0.050679478	a: 0.124	2.03
	0.2	161.6	0.074584857	b: 0.050	
	0.2	154.1	0.100364662	r2: 0.999	
	0.2	148.5	0.125424982		
	0.2	144.1	0.149586621		
G. pamelaeae in GMM + 10mM FAc-2	0	170.7	0.052079218	a: 0.128	2.03
	0.2	160.7	0.077243803	b: 0.052	
	0.2	153.5	0.102736038	r2 : 0.999	
	0.2	148	0.127889739		
	0.2	143.3	0.15431758		
G. pamelaeae in GMM + 10mM FAc-3	0	171.6	0.050286507	a: 0.122	2.00
	0.2	161.8	0.074006523	b: 0.049	
	0.4	149.9	0.11877346	r2: 0.999	
	0.6	137.4	0.196056842		
	0.8	127.5	0.293816971		
10mM FAc in GMM-1	0	169.7	0.054146173	a: 0.131	2.05
	0.2	160	0.079377235	b: 0.054	
	0.2	152.7	0.105985258	r2: 0.999	
	0.2	147.1	0.132449		
	0.2	142.6	0.158579748		
10mM FAc in GMM-2	0	171	0.05250415	a: 0.111	2.13
	0.2	165.8	0.064597328	b: 0.047	
	0.2	157.7	0.088970237	r2: 0.987	
	0.2	151.5	0.113801612		
	0.2	146.5	0.138917823		

Table S6: Kinetic parameters for the P3 haloacid dehalogenase (this study) compared to known haloacid dehalogenases DeHa2 and fluoroacetate dehalogenases DeHa4 reported by Farajollahi et al., 2024 (1) and RPA1163 reported by Mehrabi et al., 2019 (2). RPA1163 and P3 were assayed at the same pH (8.5) while DeHa2 and DeHa4 were measured at pH 7.0. The K_M and the catalytic efficiency for DeHa2 and DeHa4 is lower compared to P3 and RPA1163. FAc = fluoroacetate.

Enzyme	Substrate	pH	K_m (mM)	k_{cat} (s^{-1})	k_{cat}/K_m (s^{-1} mM^{-1})
P3 (this study)	FAc	8.5	59.8 ± 7.7	0.25 ± 0.02	4.24 ± 0.08
RPA1163	FAc	8.5	86.8 ± 13.7	1.07 ± 0.11	12.3 ± 2.3
DeHa2	FAc	7	5.47 ± 1.54	$(8.53 \pm 1.2) \times 10^{-4}$	$(1.81 \pm 0.22) \times 10^{-4}$
DeHa4	FAc	7	2.19 ± 0.37	$(2.14 \pm 0.22) \times 10^{-4}$	$(1.06 \pm 0.11) \times 10^{-4}$

Table S7: Proteins with available crystal structures used in this study for crystal structure AlphaFold comparison, comparison of MD simulations and Cα calculations.

Protein	PDB	organism	defluorination
LDexYL	1JUD	*Pseudomonas* sp. YL	No
DhlB	1AQ6	*Xanthobacter autotrophicus*	No
Rsc1362	3UMB	*Ralstonia solanacearum*	No
PA0810	3UMC	*Pseudomonas aeruginosa*	No
Rha0230	3UMG	*Rhodococcus jostii* RHA1	yes
DehIVA	2NO4	*Burkholderia cepacia*	No

Table S8: Conserved residue numbers and C_α atom indices used for the distance analysis between defluorinating and non-defluorinating HADs.

Residue number of conserved residues

Protein ID	D1	T	R1	N	K	D2	R2
DehIVa	20	24	51	129	161	190	206
DhIB	8	12	39	115	147	176	192
GKG	22	26	53	129	161	190	206
PA0810	28	32	65	143	173	202	218
Rha0230	21	25	58	139	169	198	214
RSc1362	10	14	41	123	155	184	200
WP087	8	12	39	115	147	176	192
WP118	12	16	49	129	159	188	204
WP178	12	16	49	128	158	187	203

C_α Atom Index

Protein ID	D1 Cα	T Cα	R1 Cα	N Cα	K Cα	D2 Cα	R2 Cα
DehIVa	305	355	750	2043	2531	2983	3215
DhIB	125	175	594	1800	2260	2691	2913
GKG	296	346	768	2022	2465	2940	3168
PA0810	428	483	1019	2282	2733	3184	3431
Rha0230	313	366	871	2133	2595	3023	3248
RSc1362	152	202	620	1929	2408	2831	3068
WP087	115	165	587	1841	2287	2762	2990
WP118	203	256	760	2036	2506	2951	3177
WP178	189	242	752	2102	2584	3015	3237

Table S9: Phylogenetic tree tip indices and UniProt accessions for the HAD-like superfamily enzymes included in the maximum-likelihood phylogenetic tree (Figure S20).

**Index**	**UniProt ID**	**Index**	**UniProt ID**	**Index**	**UniProt**
1	A0A099PBW3	84	P42941	167	A0A2R8Y6K4
2	A0A926DHK7 (P6)	85	P9WGJ3	168	A0A2R8YET7

3	Q51645	86	Q12A06	169	A0A2R8YGM9
4	Q53464	87	Q21YU0	170	A0A8M2B9H5
5	Q60099	88	Q5M3B3	171	A0A8M2BHD8
6	Q8XZN3	89	Q5QXU4	172	A0A8M2BHD9
7	Q9I5C9	90	Q7M7U5	173	A0A8M2BHG2
8	A0A423UMR7	91	Q9CHW3	174	A0A8M2BHU7
9	Q0SK70 (P5)	92	Q9KPM2	175	A0A8M3AP55
10	Q122Z0	93	Q9S281	176	A0A8M6YVK2
11	Q12G50	94	P60487	177	A0JME1
12	Q2IG66	95	Q3ZBF9	178	A6NCB9
13	Q96XE7	96	Q8VD52	179	B1APR7
14	Q94K71	97	Q96GD0	180	E7EQM5
15	P95649	98	P21829	181	E7ETN2
16	Q88M11	99	P27848	182	F8WB53
17	Q9HZ62	100	Q8TCD6	183	O94279
18	A4QFW4	101	Q9D9M5	184	P40081
19	Q15JF8	102	P71447	185	Q6P4T3
20	F4KFT7	103	P77366	186	Q86V88
21	Q9SU92	104	O06995	187	A0A1W2P721
22	Q8A947	105	P0A8Y3	188	A0A804HHY3
23	P53981	106	P77625	189	A0A804HL00
24	Q9P6N2	107	O58690	190	A0A8C8KDW0
25	F4JTE7	108	P76329	191	A2A953
26	P40106	109	O31156	192	B1AKW3
27	P41277	110	Q9I433	193	B7QN98
28	Q8VZP1	111	Q7ZAP3	194	E5RHZ7
29	O33194	112	D0VWZ7	195	E7ESD5

30	O07565	113	Q5XAQ1	196	E9PLN6
31	Q84VZ1	114	Q8R2H9	197	F2Z2Y1
32	A0A2K3DU55	115	Q8TCT1	198	Q8BY78
33	Q9LXR9	116	Q9FN41	199	Q8H684
34	C0QRP9	117	O31667	200	P9WPI9
35	Q12XX5	118	P0DV34	201	Q9HTR2
36	Q86ZR7	119	Q9I6F6	202	P0A8Y5
37	A0A0U5GNT1	120	P32662	203	P75792
38	P46891	121	Q9HLQ2	204	Q8VZ10
39	P0ADP0	122	O67359	205	Q9ZVJ5
40	P75809	123	P0DKC3	206	P31467
41	P42962	124	P40852	207	P77475
42	Q9LDD5	125	P40853	208	J9VX38
43	P70947	126	Q0I1W8	209	P94592
44	P96741	127	Q8GWU0	210	Q8RYE9
45	F1RIU1	128	Q6EP66	211	Q9X0Y1
46	Q498V7	129	Q84MD8	212	A0A061ACH4
47	A0A8V1ALE8	130	Q8TBE9	213	A0A061ACK7
48	C9JBI3	131	Q5M969	214	A0A061ADS3
49	E1C9F4	132	Q9CPT3	215	A0A061AKN1
50	O25367	133	P0A8Y1	216	B3DI21
51	Q8YI30	134	O32125	217	B7PHY5
52	Q8Z598	135	P0AF24	218	F1QGU0
53	V7LE91	136	P64636	219	O06480
54	Q67YC0	137	Q8DPQ3	220	O59346
55	Q9H008	138	Q9I767	221	P44447
56	Q0VD18	139	Q52087	222	P9WGJ1

57	Q9D7I5	140	P60527	223	P9WMS5
58	Q9JMQ2	141	O06652	224	Q12486
59	A0A8V1AB74	142	B2Z3V8	225	Q2QQ95
60	P77247	143	R4GRT2	226	Q2R0W7
61	P38773	144	Q01399	227	Q2UEK4
62	P38774	145	Q08623	228	Q2UEL0
63	Q52086	146	Q9D5U5	229	Q5XD45
64	Q8KLS9	147	A6NDG6	230	Q836C7
65	A0A1P8BAY5	148	D3ZDK7	231	Q8E044
66	P32626	149	Q8CHP8	232	Q8GAX4
67	Q9UHY7	150	P97767	233	Q95Q10
68	A0A8I6ADH3	151	Q99502	234	Q9U1W6
69	Q8BGB7	152	O00167	235	Q9U1W7
70	A0A1L1RQM2	153	O08575	236	Q9UTA6
71	A0A8V0XML6	154	O17670	237	Q9VYT0
72	P0C8L5	155	O82162	238	B6YTD6
73	O53289	156	O95677	239	B7Q9S3
74	O82796	157	P97480	240	G0L7V6
75	P0AGB0	158	Q05201	241	O58216
76	P78330	159	Q99504	242	Q7A484
77	A0QJI1	160	Q9D967	243	P1
78	P94512	161	A0A0G2JZ01	244	P2
79	P94526	162	A0A8I6A077	245	P3
80	Q58989	163	A0A8I6AMN9	246	P4
81	Q72H00	164	A0A8M3AZ17	247	P7
82	Q99LS3	165	Q6DBY3	248	P8
83	A7H590	166	Q9W6E8		

Table S10: Comparison betweens fluoride concentrations measured by ion chromatography and measured by colorimetric assay. The samples were taken from the alanine scanning experiment (1mM fluoroacetate, 24h incubation at 37°C).

	P6_R99A	P6_L100A	P6_W101A	P6_H102A	P6_R103A	P6_L104A	P6_P107A	P6_P108A	P6_D109A	P6_V110A
**Colorimetric μM**	62	108	116	3	5	10	34	3	108	3
**Ion Chromat. μM**	79	132	149	11	17	27	51	11	139	12
**Standard deviation**	10	24	33	8	12	17	17	8	31	9

	P6_Q111A	P6_E112A	P6_G113A	P6_K118A	P6_T119A	P6_Y121A	P6_S122A
**Colorimetric μM**	84	108	116	3	105	86	3
**Ion Chromat. μM**	108	179	120	14	122	128	14
**Standard deviation**	24	71	4	11	17	42	6

Table S11: Interfering substances and buffers for the colorimetric fluoride assay. To solutions of 0 and 100 μM fluoride the indicated concentrations of compound were added. Then it was measured if there was a change in absorption compared to the control without the compound.

**Compound and concentration**	**Interference**
13 g/liter nutrient broth	yes
0.1% yeast extract	yes
37 g/liter brain heart infusion	yes
> 500 μM sodium phosphate monobasic	yes
25% acetone	no
300 mM acetate buffer	no
20 mM HEPES	no
10% glycerol	no
100 mM sodium chloride	no
14 mM sodium bromide	no

500 µM sodium sulfate	no
10 mM sodium nitrate	no
47 µM borate (borax)	no
2 µM EDTA	no
0.1% Casamino acids	no
50 mM succinic acid	no
100 mM magnesium chloride	no
200 mM calcium chloride	no
2 mM lithium chloride	no
2% glucose	no
2% arabinose	no
2% sucrose	no
1 mM thiamine hydrochloride	no
1 mM biotin	no
0.2 M Tris	no
0.2 M MOPS	no
20 mM ferric chloride	no
20% ethanol	no
100 mM MES	no
20% DMSO	no
100 mM imidazole	no
100 mM zinc chloride	no
1 mM cupric chloride	no

10 µM sodium citrate dihydrate	no
2.5 mM ammonium chloride	no
500 mM potassium chloride	no
2% DMF	no
20% acetonitrile	no
10 mM Tricine	no
<500 µM sodium phosphate monobasic	no

Table S12: Additional HADs included in this study used for colorimetric assay development.

Protein id	NCBI identifier	Organism	Defluorination
P9	WP_050688410.1	*Priestia megaterium*	Yes
P10	WP_029378231.1	*Staphylococcus xylosus*	No
P11	WP_048310185.1	*Alkalihalobacillus macyae*	No
P12	WP_081745164.1	*Eisenbergiella massiliensis*	No
P13	WP_066861885.1	*Neglectibacter timonensis*	No
P14	WP_263041397.1	*Acutalibacter* LFL 21	No
P15	WP_187031722.1	*Oscillospiraceae*	No
P16	WP_036125194.1	*Lysinibacillus*	No
P17	WP_031546286.1	*Lacticaseibacillus rhamnosus*	No
P18	WP_053359908.1	*Clostridium butyricum*	No
P19	GKH53471	*Lachnospiraceae bacterium*	No

Table S13: P6 variants are showing reduced defluorination vs. dechlorination activity in both experiment 1 and experiment 2.

**Top P6 Protein variant influencing defluorination in both experiment 1 and 2**		
V17A	E51A	A218H
V24A	Y53A	E223A
G28A	K55A	V225A
S38A	E59A	A232H
D40A	G63A	G236A
E43A	G129A	
N46A	F157A	

Table S14: Top 48 P6 variants showing reduced defluorination activity in the alanine scanning experiment 1. The values correspond to the difference between dechlorination activity minus defluorination activity. The values were min-max normalized.

value	P6 variant	value	P6 variant	value	P6 variant	value	P6 variant
1.00	gY53A	0.70	gG236A	0.62	gT224A	0.55	gA226H
0.83	gR229A	0.69	gA233H	0.62	gG63A	0.54	gD40A
0.80	gA209H	0.69	gA232H	0.61	gK55A	0.53	gg29A
0.78	gV225A	0.68	gV210A	0.60	gP204A	0.53	gK219A
0.77	gG28A	0.67	gE22A	0.60	gK35A	0.53	gR103A
0.75	gS38A	0.66	gR234A	0.59	gL199A	0.53	gE51A
0.73	gN46A	0.65	gE223A	0.59	gP106A	0.52	gF157A
0.72	gL117A	0.65	gT94A	0.59	gV24A	0.52	gV237A
0.72	gP125A	0.63	gP202A	0.58	gg27A	0.52	gg43A
0.72	gE217A	0.63	gA218H	0.58	gG129A	0.52	gP197A
0.71	gV214A	0.63	gD221A	0.57	gG52A	0.51	gE59A
0.71	gG36A	0.63	gL133A	0.56	gN212A	0.51	gD71A

Table S15: Top 48 P6 variants showing reduced defluorination activity in the alanine screening experiment 2. The values correspond to dechlorination activity minus defluorination activity normalized with protein concentrations and to wild type protein (%).

value	P6 variant	value	P6 variant	value	P6 variant	value	P6 variant
1.314	gY53A	0.539	gQ26A	0.378	gL87A	0.304	gV37A
0.897	gV24A	0.516	gG236A	0.376	gG129A	0.296	gM57A
0.861	gS38A	0.506	gS4A	0.373	gV3A	0.291	gA218H
0.784	gV17A	0.469	gD40A	0.373	gH213A	0.274	gE215A
0.747	gR75A	0.461	gG28A	0.355	gI10A	0.269	gL104A

0.741	gI39A	0.456	gE59A	0.355	gE91A	0.253	gT90A
0.668	gL89A	0.432	gK88A	0.347	gK55A	0.251	gF157A
0.665	gD2A	0.426	gV18A	0.344	gA232H	0.251	gN46A
0.658	gE93A	0.408	gV56A	0.344	gY220A	0.242	gV6A
0.601	gI194A	0.403	gK77A	0.336	gF228A	0.240	gE50A
0.599	gR7A	0.385	gA30H	0.330	gE43A	0.228	gV25A
0.551	gH42A	0.382	gE51A	0.309	gV225A	0.220	gG63A

Table S16: Random forest classification accuracy based on 1000 distinct models with 80%/20% training test splits for full-length protein features and C-terminal features (final 41 amino acids). Experiment 1 was conducted on data without protein concentration normalization and without case weighting. Experiment 2 was conducted after dilution 1:3, with normalization for protein concentration and with case weighting. For this reason datasets were not combined however both yielded comparable results.

	**Full-length protein model**	**C-terminus only model**
**Experiment 1 (min-max normalized)**		
Training accuracy	0.948 ± 0.008	0.832 ± 0.023
Testing accuracy	0.948 ± 0.020	0.832 ± 0.078
Testing-F1 score	0.928 ± 0.028	0.728 ± 0.129
**Experiment 2**	**(diluted with protein concentration normalized)**	
	**Full-length protein model**	**C-terminus only model**
Training accuracy	0.877 ± 0.003	0.800 ± 0.021
Testing accuracy	0.863 ± 0.006	0.791 ± 0.033

Table S17: Five HADs used for machine learning validation highlighting confident defluorination prediction for proteins with high defluorination prediction probabilities (P20)

compared to non-defluorination proteins with low prediction probabilities (P21-P24). *Defluorination probability predicted by random forest (see Methods).

Protein id	NCBI identifier	Organism	Defluorination	Defluorination probability*
P20	WP_139652913.1	*Raoultibacter phocaeensis*	Yes	0.85 ± 0.19
P21	HTS94301.1	*Stellaceae bacterium*	No	0.54 ± 0.09
P22	PYN88313.1	*Ca. Rokuibacteriota*	No	0.43 ± 0.06
P23	MDH3205452.1	*Gemmatimonadota bacterium*	No	0.57 ± 0.10
P24	MBS6900484.1	*Eubacterium sp.*	No	0.38 ± 0.06

Table S18: Defluorination data from the alanine scanning library used for machine learning and Figure 4D. exp1 = Experiment 1, exp2 = Experiment 2, sd = standard deviation.

label	mean exp1	sd exp1	mean exp2	sd exp2	label	mean exp1	sd exp1	mean exp2	sd exp2
gD2A	0.4136	0.2258	0.1213	0.016	gK120A	0.5491	0.1361	0.2402	0.056
gV3A	0.1457	0.0639	0.0515	0.0324	gY121A	0.1511	0.1288	0.0021	0.0014
gS4A	0.1973	0.1272	0.2561	0.0631	gS122A	0.8013	0.0668	0.8107	0.1928
gN5A	0.2757	0.1397	0.1303	0.0359	gI123A	0.1079	0.0932	0.0048	0.003
gV6A	0.1922	0.0691	0.0346	0.0186	gG124A	0.1994	0.0819	0.0698	0.0314
gR7A	0.3105	0.0559	0.0788	0.0227	gP125A	0.3274	0.0329	0.1544	0.0486
gI8A	0.3276	0.1364	0.0355	0.0168	gF126A	0.1163	0.1645	0.0049	0.0032
gV9A	0.2828	0.1080	0.0114	0.0041	gS127A	0.1837	0.0787	0.0118	0.0076
gI10A	0.3694	0.1784	0.0862	0.0221	gN128A	0.2146	0.1137	0.0051	0.0012
gF11A	0.1792	0.0538	0.0063	0.0022	gG129A	0.3259	0.1466	0.097	0.006
gD12A	0.1713	0.1767	0.0222	0.0393	gD130A	0.2204	0.0153	0.0043	0.0036
gT13A	0.4883	0.3034	0.2666	0.0355	gF131A	0.1563	0.0305	0.0042	0.0048
gF14A	0.1239	0.0056	0.0026	0.0013	gR132A	0.2448	0.0534	0.0409	0.028
gG15A	0.1352	0.0208	0.0037	0.0022	gL133A	0.4262	0.0738	0.2423	0.1028
gT16A	0.2355	0.1875	0.0045	0.0029	gL134A	0.2597	0.0877	0.0027	0.0062
gV17A	0.4426	0.1063	0.0619	0.0051	gL135A	0.2652	0.0096	0.0211	0.0099
gV18A	0.5939	0.0323	0.3115	0.1565	gN136A	0.2927	0.0818	0.0124	0.0076
gN19A	0.1456	0.0108	0.0073	0.002	gM137A	0.2372	0.1135	0.0042	0.0056
gW20A	0.0821	0.0799	0.0029	0.0033	gA138H	0.2028	0.0312	0.0028	0.0033
gH21A	0.1920	0.0810	0.0023	0.0021	gK139A	0.3005	0.0589	0.1056	0.0386
gE22A	0.3321	0.1690	0.2808	0.0648	gG140A	1.0000	0.0000	1	0.069
gS23A	0.9337	0.0067	0.8136	0.2154	gS141A	0.9555	0.0000	0.6997	0.1393
gV24A	0.4228	0.0680	0.1697	0.0277	gG142A	0.6578	0.1714	0.2756	0.0375
gV25A	0.4338	0.0811	0.1404	0.0247	gL143A	0.2345	0.0521	0.0022	0.0021
gQ26A	0.5371	0.0273	0.3006	0.1335	gP144A	0.5462	0.1130	0.2055	0.0604
gE27A	0.3779	0.0339	0.21	0.0988	gW145A	0.1908	0.0948	0.003	0.0044
gG28A	0.2606	0.0716	0.0381	0.0041	gD146A	0.2377	0.0593	0.0081	0.0088
gE29A	0.8064	0.0000	0.2841	0.1177	gF147A	0.2124	0.0449	0.0025	0.0028
gA30H	0.1342	0.1485	0.1395	0.046	gI148A	0.2489	0.0631	0.002	0.0029
gL31A	0.1897	0.0142	0.0209	0.0009	gL149A	0.2147	0.0561	0.0044	0.0047
gG32A	0.8905	0.0000	0.2804	0.106	gA150H	0.2900	0.1177	0.0042	0.0042

gR33A	0.5721	0.1228	0.3307	0.0789	gG151A	0.9029	0.0240	0.4914	0.1592
gA34H	0.6273	0.0330	0.2813	0.1306	gQ152A	0.3513	0.1585	0.0242	0.0072
gK35A	0.4260	0.0418	0.2739	0.099	gQ153A	0.6693	0.0000	0.1983	0.0618
gG36A	0.3326	0.0699	0.3114	0.1285	gF154A	0.2262	0.0373	0.0043	0.0039
gV37A	0.5209	0.2900	0.2155	0.0322	gQ155A	0.4786	0.0862	0.1982	0.0906
gS38A	0.2779	0.0682	0.1922	0.0544	gK156A	0.2182	0.0210	0.008	0.0062
gI39A	0.2691	0.0499	0.0747	0.0057	gF157A	0.2733	0.0545	0.039	0.0238
gD40A	0.2873	0.0402	0.1178	0.0535	gK158A	0.2783	0.0679	0.0039	0.0048
gW41A	0.1173	0.0723	0.0042	0.0077	gP159A	0.2871	0.0266	0.0051	0.0027
gH42A	0.5786	0.1522	0.2726	0.1467	gD160A	0.3520	0.1605	0.0778	0.0206
gE43A	0.4584	0.0607	0.2291	0.0581	gP161A	0.5578	0.0000	0.0161	0.0067
gF44A	0.0754	0.0794	0.0039	0.0045	gT162A	0.4035	0.0104	0.1306	0.0331
gA45H	0.0601	0.0778	0.0026	0.0022	gI163A	0.2406	0.0261	0.0097	0.005
gN46A	0.2937	0.0613	0.1233	0.0301	gY164A	0.2500	0.0822	0.0023	0.0054
gV47A	0.4704	0.1406	0.2593	0.0668	gE165A	0.6613	0.0151	0.4182	0.0631
gW48A	0.0917	0.0805	0.0058	0.0064	gD166A	0.4622	0.2062	0.279	0.0799
gR49A	0.1931	0.0673	0.0054	0.0055	gA167H	0.2530	0.0436	0.0021	0.0067
gE50A	0.5223	0.0531	0.2676	0.1031	gV168A	0.3033	0.0612	0.0066	0.0069
gE51A	0.4349	0.0651	0.2505	0.0925	gE169A	0.7590	0.1002	0.5186	0.171
gG52A	0.3975	0.0503	0.3359	0.2261	gL170A	0.2713	0.0004	0.005	0.0033
gY53A	0.1848	0.0578	0.036	0.0188	gL171A	0.2433	0.0017	0.0044	0.0049
gI54A	0.5080	0.1128	0.329	0.1864	gG172A	0.2652	0.1067	0.003	0.0036
gK55A	0.3678	0.0505	0.2541	0.128	gG173A	0.2802	0.0894	0.0995	0.037
gV56A	0.2450	0.0666	0.0455	0.0201	gR174A	0.5235	0.1751	0.3157	0.0719
gM57A	0.5581	0.1231	0.2928	0.0608	gP175A	0.6953	0.0745	0.3307	0.0978
gY58A	0.4852	0.0452	0.1589	0.0702	gE176A	0.4983	0.0719	0.2514	0.1028
gE59A	0.4709	0.0504	0.2746	0.0514	gE177A	0.2446	0.1074	0.0092	0.0077
gV60A	0.2370	0.0310	0.1199	0.0404	gV178A	0.2768	0.0101	0.0058	0.0047
gA61H	0.2478	0.1728	0.0327	0.0225	gL179A	0.3916	0.0470	0.1547	0.0477
gQ62A	0.5896	0.0067	0.5254	0.2317	gM180A	0.2483	0.0757	0.0044	0.0013
gG63A	0.3292	0.0172	0.2661	0.0979	gV181A	0.2725	0.0413	0.0121	0.0099
gL64A	0.4749	0.0270	0.366	0.2342	gA182H	0.2373	0.1206	0.0063	0.0063
gR65A	0.5078	0.0515	0.3566	0.2015	gA183H	0.2509	0.0629	0.0093	0.0114
gP66A	0.6970	0.0617	0.329	0.16	gH184A	0.2921	0.0666	0.0027	0.0045

gW67A	0.2493	0.2401	0.0061	0.009	gP185A	0.5053	0.2037	0.2888	0.0741
gE68A	0.3236	0.2124	0.2107	0.0719	gS186A	0.6204	0.0540	0.2379	0.0088
gP69A	0.5015	0.2081	0.3838	0.1305	gD187A	0.2515	0.0242	0	0.0014
gV70A	0.1262	0.0059	0.0051	0.0066	gL188A	0.2600	0.1512	0.0082	0.0089
gD71A	0.2376	0.0675	0.0303	0.0183	gD189A	0.2884	0.0460	0.0046	0.0045
gV72A	0.2725	0.1621	0.008	0.0065	gG190A	0.5091	0.0852	0.1	0.0378
gL73A	0.1313	0.0161	0.0245	0.0129	gA191H	0.3394	0.1135	0.0063	0.0056
gH74A	0.3164	0.0074	0.0096	0.0148	gH192A	0.9673	0.0000	0.5221	0.1045
gR75A	0.3252	0.0015	0.0824	0.005	gA193H	0.5890	0.1499	0.3569	0.0832
gR76A	0.3043	0.0319	0.247	0.0861	gI194A	0.2272	0.0416	0.0409	0.0079
gK77A	0.3762	0.1182	0.1204	0.0269	gG195A	0.0892	0.0091	0.0075	0.0139
gL78A	0.1798	NA	0.0139	0.0188	gC196A	0.2361	0.0976	0.0327	0.0467
gD79A	0.4822	0.0604	0.5085	0.3439	gP197A	0.4561	0.0539	0.2688	0.124
gE80A	0.5469	0.0384	0.44	0.3203	gT198A	0.0788	0.0475	0.0078	0.0072
gL81A	0.2500	0.0174	0.0077	0.0095	gL199A	0.2781	0.0214	0.2234	0.0848
gL82A	0.6384	0.0547	0.2554	0.0728	gY200A	0.1339	0.1153	0.1539	0.0697
gD83A	0.5906	0.1441	0.3814	0.1799	gV201A	0.0952	0.0561	0.0122	0.0053
gV84A	0.5399	0.0607	0.2937	0.0837	gP202A	0.2310	0.0667	0.0586	0.0252
gY85A	0.4632	0.1722	0.2214	0.1356	gR203A	0.0714	0.0254	0.0047	0.0065
gG86A	0.4495	0.0882	0.4361	0.3551	gP204A	0.3489	0.0212	0.2112	0.0217
gL87A	0.2879	0.0689	0.0167	0.0096	gL205A	0.4286	0.0004	0.347	0.1157
gK88A	0.5715	0.0559	0.3104	0.1286	gE206A	0.1005	0.0161	0.005	0.008
gL89A	0.5111	0.0517	0.106	0.0398	gY207A	0.0644	0.0231	0.0012	0.0019
gT90A	0.7042	0.0915	0.3317	0.2088	gG208A	0.0968	0.0158	0.009	0.0138
gE91A	0.6371	0.0472	0.3696	0.198	gA209H	0.3107	0.0465	0.2968	0.1262
gE92A	0.6167	0.1010	0.4529	0.3318	gV210A	0.3492	0.0398	0.327	0.1268
gE93A	0.4263	0.1783	0.066	0.009	gN211A	0.3123	0.1716	0.269	0.0808
gT94A	0.4140	0.2512	0.4376	0.2855	gN212A	0.4144	0.0669	0.2726	0.0739
gD95A	0.6896	0.0104	0.4895	0.208	gH213A	0.1448	0.0490	0.0267	0.0058
gH96A	0.5300	0.0482	0.3414	0.0839	gV214A	0.3361	0.0061	0.2408	0.0085
gF97A	0.2515	0.0386	0.0118	0.0089	gE215A	0.1204	0.0215	0.0644	0.0229
gN98A	0.5823	0.0104	0.2869	0.0369	gP216A	0.3582	0.0325	0.2317	0.0922
gR99A	0.7416	0.0000	0.2851	0.009	gE217A	0.3649	0.0522	0.3208	0.1874
gL100A	0.7226	0.0000	0.277	0.0627	gA218H	0.2716	0.0761	0.1923	0.0739

gW101A	0.1031	0.0675	0.0052	0.0027	gK219A	0.2922	0.0481	0.2751	0.0909
gH102A	0.3459	0.1577	0.0128	0.0045	gY220A	0.1752	0.0176	0.0339	0.0088
gR103A	0.3532	0.0166	0.0248	0.0179	gD221A	0.3783	0.0992	0.3449	0.136
gL104A	0.5109	0.1236	0.2282	0.0208	gH222A	0.0822	0.0700	0.0064	0.0037
gL105A	0.8244	0.1746	0.4598	0.0579	gE223A	0.2384	0.0176	0.2549	0.0697
gP106A	0.3295	0.0338	0.0803	0.012	gT224A	0.3693	0.0012	0.3114	0.0795
gW107A	0.1160	0.1174	0.0006	0.0014	gV225A	0.2552	0.0614	0.169	0.0392
gP108A	0.5080	0.0603	0.2411	0.0653	gA226H	0.3996	0.1008	0.2902	0.0724
gD109A	0.2215	0.0000	0.0032	0.004	gD227A	0.0815	0.0107	0.0007	0.001
gV110A	0.4678	0.2630	0.3849	0.2342	gF228A	0.1122	0.0185	0.0016	0.0022
gQ111A	0.5112	0.0189	0.3602	0.1944	gR229A	0.2787	0.1243	0.2526	0.095
gE112A	0.4456	0.1612	0.2952	0.0103	gE230A	0.8656	0.0000	0.3191	0.1007
gG113A	0.7484	0.1985	0.5705	0.0881	gL231A	0.0313	0.0443	0.0077	0.006
gL114A	0.1624	0.0138	0.0047	0.0039	gA232H	0.2064	0.0488	0.0243	0.0154
gR115A	0.5597	0.1369	0.4441	0.0455	gA233H	0.3840	0.0031	0.3409	0.0939
gR116A	0.5653	0.0367	0.3634	0.0763	gR234A	0.3410	0.0257	0.3396	0.1373
gL117A	0.1191	0.0117	0.0037	0.004	gL235A	0.0708	0.0419	0.01	0.0091
gK118A	0.4547	0.2647	0.2779	0.0274	gG236A	0.3461	0.0036	0.0996	0.0735
gT119A	0.4841	0.0391	0.3245	0.0993	gV237A	0.4622	0.0193	0.3715	0.1677

Table S19: Organisms used in this study

**Strains**	**Genotype and specifications**	**Origin**
E. coli DH5α	*fhuA2Δ(argF-lacZ)U169 phoA glnV44 Φ80Δ(lacZ)M15 gyrA96 recA1 relA1 endA1 thi-1 hsdR17*	New England BioLabs
T7 Express *E. coli*	*fhuA2 lacZ::T7 gene1 [lon] ompT gal sulA11 R(mcr-73::miniTn10--*Tet^S*)2 [dcm] R(zgb-210::Tn10--*Tet^S*) endA1 Δ(mcrC-mrr)114::IS10*	New England BioLabs

Gordonibacter pamelaeae DSM 110924	WT, isolated from human feces	DSMZ

Table S20: Plasmids generated in this study

Plasmid name	Parent plasmid	Description	Inducer
pCDFDuet-1-WP118709078 (P1)	pCDFDuet-1	*spec*^{R}, *wp118709078.1*	IPTG
pCDFDuet-1-WP087189991 (P2)	pCDFDuet-1	*spec*^{R}, *wp087189991.1*	IPTG
pCDFDuet-1-GKG91607 (P3)	pCDFDuet-1	*spec*^{R}, *gkg91607*	IPTG
pCDFDuet-1-WP004693440 (P4)	pCDFDuet-1	*spec*^{R}, *wp004693440.1*	IPTG
pCDFDuet-1-3UMG (P5-control)	pCDFDuet-1	*spec*^{R}, *3UMG-1*	IPTG
pCDFDuet-1-WP178618037 (P6)	pCDFDuet-1	*spec*^{R}, *wp178618037.1*	IPTG
pCDFDuet-1-WP006686258 (P7)	pCDFDuet-1	*spec*^{R}, *wp006686258.1*	IPTG
pCDFDuet-1-WP053428405 (P8)	pCDFDuet-1	*spec*^{R}, *wp053428405.1*	IPTG
pCDFDuet-1-WP050688410 (P9)	pCDFDuet-1	*spec*^{R}, *wp*050688410.*1*	IPTG
pCDFDuet-1-WP029378231 (P10)	pCDFDuet-1	*spec*^{R}, *wp*029378231.*1*	IPTG
pCDFDuet-1-WP048310185 (P11)	pCDFDuet-1	*spec*^{R}, *wp048310185.1*	IPTG
pCDFDuet-1-WP081745164 (P12)	pCDFDuet-1	*spec*^{R}, *wp*081745164.*1*	IPTG
pCDFDuet-1-WP066861885 (P13)	pCDFDuet-1	*spec*^{R}, *wp066861885.1*	IPTG
pCDFDuet-1-WP263041397 (P14)	pCDFDuet-1	*spec*^{R}, *wp*263041397.*1*	IPTG
pCDFDuet-1-WP187031722 (P15)	pCDFDuet-1	*spec*^{R}, *wp*187031722.*1*	IPTG
pCDFDuet-1-WP036125194 (P16)	pCDFDuet-1	*spec*^{R}, *wp036125194.1*	IPTG
pCDFDuet-1-WP031546286 (P17)	pCDFDuet-1	*spec*^{R}, *wp031546286.1*	IPTG
pCDFDuet-1-WP053359908 (P18)	pCDFDuet-1	*spec*^{R}, *wp053359908.1*	IPTG
pCDFDuet-1-GKH53471 (P19)	pCDFDuet-1	*spec*^{R}, *gkh53471*	IPTG

pCDFDuet-1-WP13943301 (P20)	pCDFDuet-1	*spec^R, wp13943301.1*	IPTG
pCDFDuet-1-HTS9340 (P21)	pCDFDuet-1	*spec^R, hts9340*	IPTG
pCDFDuet-1-PYN88313 (P22)	pCDFDuet-1	*spec^R, pyn88313*	IPTG
pCDFDuet-1-MDH3205452 (P23)	pCDFDuet-1	*spec^R, mdh3205452*	IPTG
pCDFDuet-1-MBS6900484 (P24)	pCDFDuet-1	*spec^R, mbs6900484*	IPTG
pCDFDuet-1-WP118709078-F54I	pCDFDuet-1	*spec^R*, WP118709078-F54I	IPTG
pCDFDuet-1-WP118709078-T57M	pCDFDuet-1	*spec^R*, WP118709078-T57M	IPTG
pCDFDuet-1-WP118709078-F74H	pCDFDuet-1	*spec^R*, WP118709078-F74H	IPTG
pCDFDuet-1-WP118709078-F187S	pCDFDuet-1	*spec^R*, WP118709078-F187S	IPTG
pCDFDuet-1-WP118709078-F54I-T57M	pCDFDuet-1	*spec^R*, WP118709078-F54I-T57M	IPTG
pCDFDuet-1-WP118709078-F54I-F74H	pCDFDuet-1	*spec^R*, WP118709078-F54I-F74H	IPTG
pCDFDuet-1-WP118709078-F54I-F187S	pCDFDuet-1	*spec^R*, WP118709078-F54I-F187S	IPTG
pCDFDuet-1-WP118709078-F54I-T57M-F74H	pCDFDuet-1	*spec^R*, WP118709078-F54I-T57M-F74H	IPTG
pCDFDuet-1-WP118709078-F54I-T57M-F187S	pCDFDuet-1	*spec^R*, WP118709078-F54I-T57M-F187S	IPTG
pCDFDuet-1-WP118709078-F54I-F74H-F187S	pCDFDuet-1	*spec^R*, WP118709078-F54I-F74H-F187S	IPTG
pCDFDuet-1-WP118709078-F54I-T57M-F74H-F187S	pCDFDuet-1	*spec^R*, WP118709078-F54I-T57M-F74H-F187S	IPTG
pCDFDuet-1-WP1187090781-F54I-T57M-F74H-F187S-1-35-WP1786180371	pCDFDuet-1	*spec^R*, pCDFDuet-1-WP1187090781-F54I-T57M-F74H-F187S AA substitutes 1-35 with WP1786180371	IPTG
pCDFDuet-1-WP-1187090781-F54I-T57M-F74H-F187S-36-69-WP1786180371	pCDFDuet-1	*spec^R*, pCDFDuet-1-WP1187090781-F54I-T57M-F74H-F187S AA substitutes 36-69 with WP1786180371	IPTG
pCDFDuet-1-WP-1187090781-F54I-T57M-F74H-F187S-70-109-WP1786180371	pCDFDuet-1	*spec^R*, pCDFDuet-1-WP1187090781-F54I-T57M-F74H-F187S AA substitutes 70-109 with WP1786180371	IPTG

pCDFDuet-1-WP-1187090781-F54I-T57M-F74H-F187S-110-150-WP1786180371	pCDFDuet-1	$spec^R$, pCDFDuet-1-WP1187090781-F54I-T57M-F74H-F187S AA substitutes 110-150 with WP1786180371	IPTG
pCDFDuet-1-WP-1187090781-F54I-T57M-F74H-F187S-150-196-WP1786180371	pCDFDuet-1	$spec^R$, pCDFDuet-1-WP1187090781-F54I-T57M-F74H-F187S AA substitutes 150-196 with WP1786180371	IPTG
pCDFDuet-1-WP-1187090781-F54I-T57M-F74H-F187S-197-end-WP1786180371	pCDFDuet-1	$spec^R$, pCDFDuet-1-WP1187090781-F54I-T57M-F74H-F187S AA substitutes 197-238 with WP1786180371	IPTG

Table S21: Primers used in this study

**Primer name**	**Sequence**
**Site directed mutagenesis P1**	
FW-WP118709078-F54I	CGCAATGATGGCTATattAAGGCAACC
RV-WP118709078-F54I	CCATCCGTTGGCAAATTCATCCCAATC
FW-WP118709078-T57M	GCAatgTTTGATATCGCCCATGGTAAGC
RV-WP118709078-T57M	CTTAAAATAGCCATCATTGCGCCATCCG
FW-WP118709078-F54T-T57M	CTATattAAGGCAatgTTTGATATCG
RV-WP118709078-F54T-T57M	CCATCATTGCGCCATCCG
FW-WP118709078-F74H	CTGATACTGTGcatATGGAATACC
RV-WP118709078-F74H	CAGGCACCCATTCACGC
FW-WP118709078-F187S	CTCATCCTagtGACCTTGAC
RV-WP118709078-F187S	CGGCAACCATTGCAACCTC
**Chimeric protein P1/P6 segment AA1-36**	
FW-WP118709078-1-36	GTGCCAAATCGTTAGATTTAGATTGGGATGAATTTGC
RV-WP118709078-1-36	GCTTACATCCATGTATATCTCCTTATTAAAGTTAAACAAAATTATTTC
FW-WP1786180371-1-35	GGAGATATACATGGATGTAAGCAATGTGCGTATTG

RV-WP1786180371-1-35	CTAAATCTAACGATTTGGCACGCCCTAATGCTTC

Chimeric protein P1/P6 segment AA 36-69

FW-WP1786180371-36-69	CGAAAAATACGGGGTTAGTATTGACTGGCAC
RV-WP1786180371-36-69	GTATCAGCAGGTTCCCAAGGGCGC
RV-WP118709078-36-69	CAATACTAACCCCGTATTTTTCGTTTAACGCTTGAC
FW-WP118709078-36-69	CCTGCTGATACTGTGcatATGGAATACCTTG

Chimeric protein P1/P6 segment AA 70-109

FW-WP1786180371-70-109	GTGCCTGTTGACGTGTTGCATCGTCGC
RV-WP1786180371-70-109	CAGGACGTCCGGCCATGGAAGCAG
FW-WP118709078-70-109	CCGGACGTCCTGGAGGGGCTGAATCG
RV-WP118709078-70-109	CACGTCAACAGGCACCCATTCACGCTTACC

Chimeric protein P1/P6 segment AA 110-150

FW-WP1786180371-110-150	CCGGATGTGCAGGAGGGGCTGCG
RV-WP1786180371-110-150	GAATAAGTCCCCTGCTAAAATGAAGTCCCACGGTAAG
FW-WP118709078-110-150	CATTTTAGCAGGGGACTTATTCCAGAAGTTC
RV-WP118709078-110-150	CTGCACATCCGGCCACGGTGAAAG

Chimeric protein P1/P6 segment AA 151-196

FW-WP1786180371-151-196	CGACCGGCCAGCAGTTCCAGAAATTTAAG
RV-WP1786180371-151-196	GTGGTACATCCGATTGCGTGCGCG
FW-WP118709078-151-196	CAATCGGATGTACCACCATCTTCGTGCCGC
RV-WP118709078-151-196	GCTGGCCGGTCGTAATAAAATCCCACGGC

Chimeric protein P1/P6 segment AA 197-238

FW-WP1786180371-197-238	GGCAGGGTGCCCAACCTTATATGTACCTCGC
RV-WP1786180371-197-238	CAGATTCTCGACTCCAAGGCGAGCGG

FW-WP118709078-197-238	CCTTGGAGTCGAGAATCTGTATTTTCAGAGCCATCACC
RV-WP118709078-197-238	CATATAAGGTTGGGCACCCTGCCAATTTAGCTCCGTC

References

1. S. Farajollahi, *et al.*, Defluorination of Organofluorine Compounds Using Dehalogenase Enzymes from *Delftia acidovorans* (D4B). *ACS Omega* (2024). https://doi.org/10.1021/acsomega.4c02517.

2. P. Mehrabi, *et al.*, Substrate-Based Allosteric Regulation of a Homodimeric Enzyme. *Journal of the American Chemical Society* (2019). https://doi.org/10.1021/jacs.9b03703.
